# Synthesis of a Coumarin-Based Analogue of Schweinfurthin F

**DOI:** 10.1021/acs.joc.1c02046

**Published:** 2021-10-29

**Authors:** Chloe M. Schroeder, Patrick N. Dey, John A. Beutler, David F. Wiemer

**Affiliations:** † Department of Chemistry, University of Iowa, Iowa City, Iowa 52242-1294, United States; ‡ Molecular Targets Program, Center for Cancer Research, NCI-Frederick, Frederick, Maryland 21702, United States

## Abstract

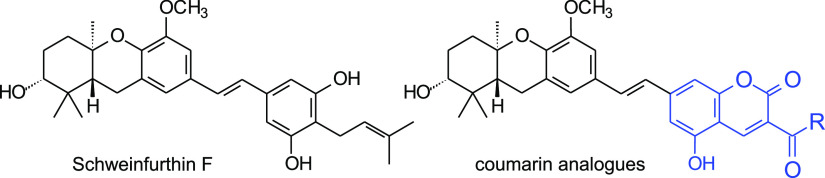

The natural schweinfurthins are stilbenes with significant antiproliferative activity and an uncertain mechanism of action. To obtain a fluorescent analogue with minimal deviation from the natural structure, a coumarin ring system was annulated to the D-ring, creating a new analogue of schweinfurthin F. This stilbene was prepared through a convergent synthesis, with a Horner–Wadsworth–Emmons condensation employed to form the central stilbene olefin. After preparation of a tricyclic phosphonate via a recent and more efficient modification of the classic Arbuzov reaction, condensation was attempted with an appropriately substituted bicyclic aldehyde but the coumarin system did not survive the reaction conditions. When olefin formation preceded generation of the coumarin, the stilbene formation proceeded smoothly and ultimately allowed access to the targeted coumarin-based schweinfurthin analogue. This analogue displayed the desired fluorescence properties along with significant biological activity in the National Cancer Institute’s 60-cell line bioassay, and the pattern of this biological activity mirrored that of the natural product schweinfurthin F. This approach gives facile access to new fluorescent analogues of the natural schweinfurthins and should be applicable to other natural stilbenes as well.

## Introduction

**Figure 1:**
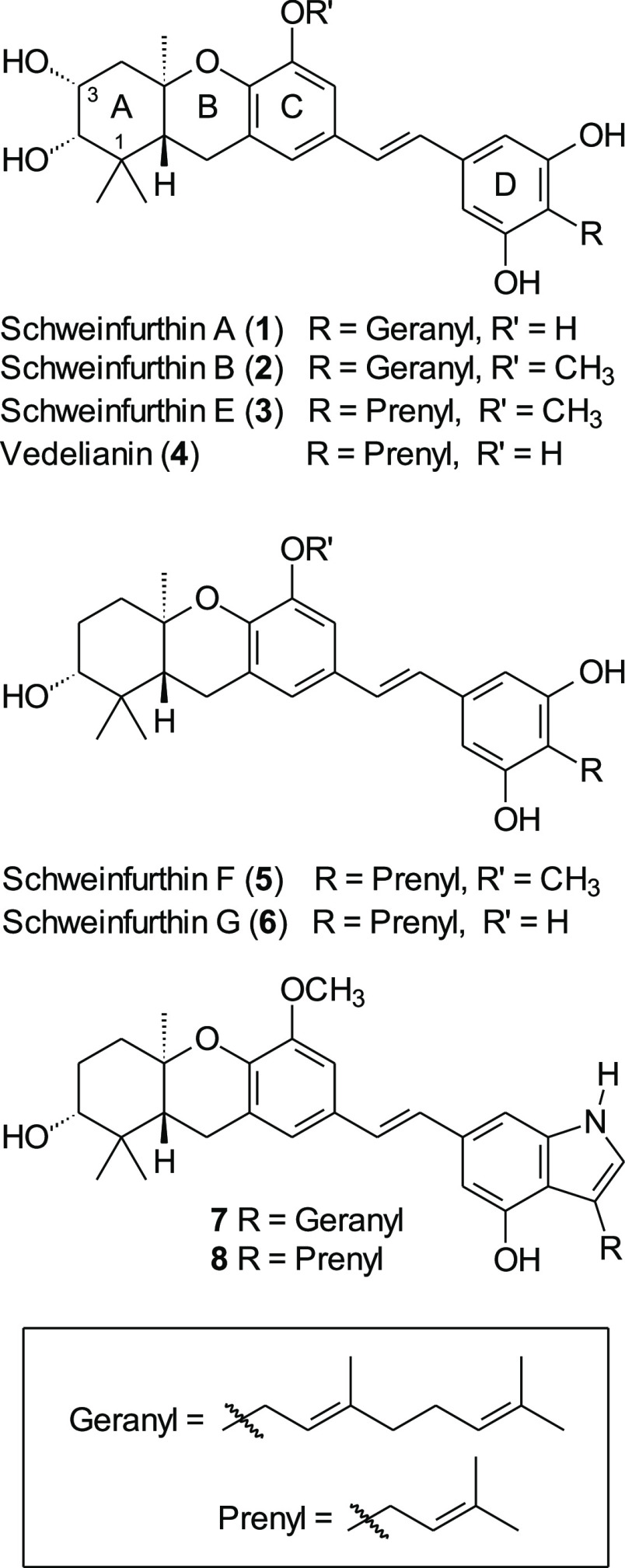
Relevant natural schweinfurthins and two indole analogues.

The schweinfurthins (Figure 1) are a small group of natural products isolated, at least thus far, from plants of the genus *Macaranga* (Euphorbiaceae) directly^1,2^ or indirectly from propolis produced by bees visiting *Macaranga* plants.^3^ The combination of an unusual pattern of differential activity in the National Cancer Institute’s (NCI’s) 60-cell line screen^4,5^ and isolation efforts that have resulted in limited and sometimes poorly reproducible quantities^6^ has encouraged us to pursue efforts to synthesize these compounds and a variety of analogues. To date, we have reported the total synthesis of several natural schweinfurthins that include the hexahydroxanthene system [A (**1**),^7^ B (**2**),^8^ E (**3**),^8^ F (**5**),^9^ G (**6**),^10^ and vedelianin (**4**)],^11^ including one as both enantiomers to allow determination of the absolute stereochemistry of the natural products (Figure 1). We also have prepared approximately 90 analogues that have been evaluated in the NCI’s 60-cell line screen for structure activity studies.^7−9,12,13^ Results of these studies indicate that the A/B/C ring system and a stilbene in the *trans* orientation are essential to the selective antiproliferative activity of these compounds, while modifications of the D-ring are generally better tolerated. Of special significance, studies of structure–activity relationships and chemical stability have revealed that the D-ring resorcinol may limit the schweinfurthins’ stability. Thus, optimum placement of a coumarin system might preserve the biological activity and simultaneously improve the chemical stability. Methylation of one of the symmetric D-ring phenolic groups has been shown to significantly increase chemical stability and testing in the NCI-60 assay revealed that there is little or no loss in activity relative to the corresponding non-methylated compounds. Furthermore, the indole analogues **7** and **8** also have shown significant activity, suggesting a tolerance for substitution at a single phenolic position.^14^

**Figure 2:**
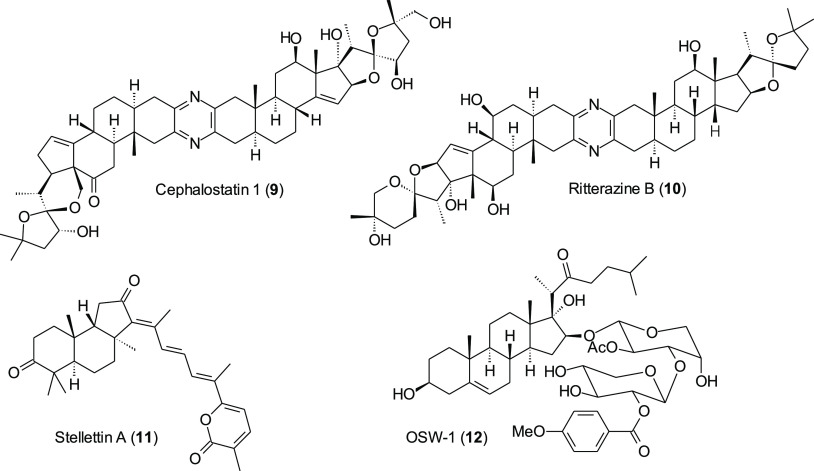
Natural products that display biological activity similar to that of schweinfurthins, based on COMPARE analyses.

The NCI created the COMPARE algorithm to associate bioactivity data from each candidate in the NCI-60 bioassay with those from other compounds that have also been through the screening process and function by a similar mechanism of action rather than by structural similarity.^15^ In the COMPARE analysis, the schweinfurthin family did not pose biological resemblance to that of any chemotherapeutic agent currently in use, but rather, the activity of the schweinfurthin family most closely resembles that of the cephalostatins (e.g. **9**), the ritterazines (e.g. **10**), the stellettins (e.g. **11**), and OSW-1 (**12**, Figure 2).^13^ Deeper investigation into the mechanism of action of the schweinfurthins by several groups has not yet provided a complete and clear mode of action. Studies have suggested interactions between several targets including oxysterol binding proteins,^16−18^ trans-Golgi-network trafficking,^19^ and the production and export of cholesterol^20^ and other products of isoprenoid biosynthesis.^21^

**Figure 3:**
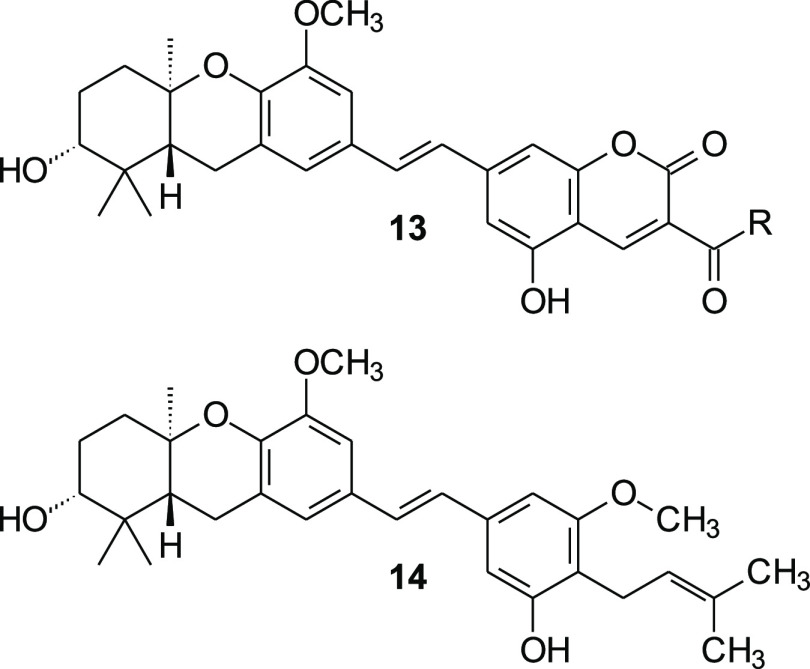
Comparison of a coumarin-containing schweinfurthin (**13**) with a mono-methylated schweinfurthin F (**14**).

To increase understanding of the mechanism of action for the schweinfurthins, it might be useful to prepare a fluorescent analogue, as long as that analogue displays significant biological activity. To maximize the possibility of activity, it appeared prudent to anneal a coumarin ring to the D-ring, as suggested in structure **13** (Figure 3). Compound **13** would preserve the complete hexahydroxanthene system of a mono-methylated schweinfurthin F (**14**), the *trans*-stilbene, and one free phenol in the D-ring. Thus, preparation of the schweinfurthin analogues in the form of structure **13** became our goal.

## Results and Discussion

**Scheme 1:**
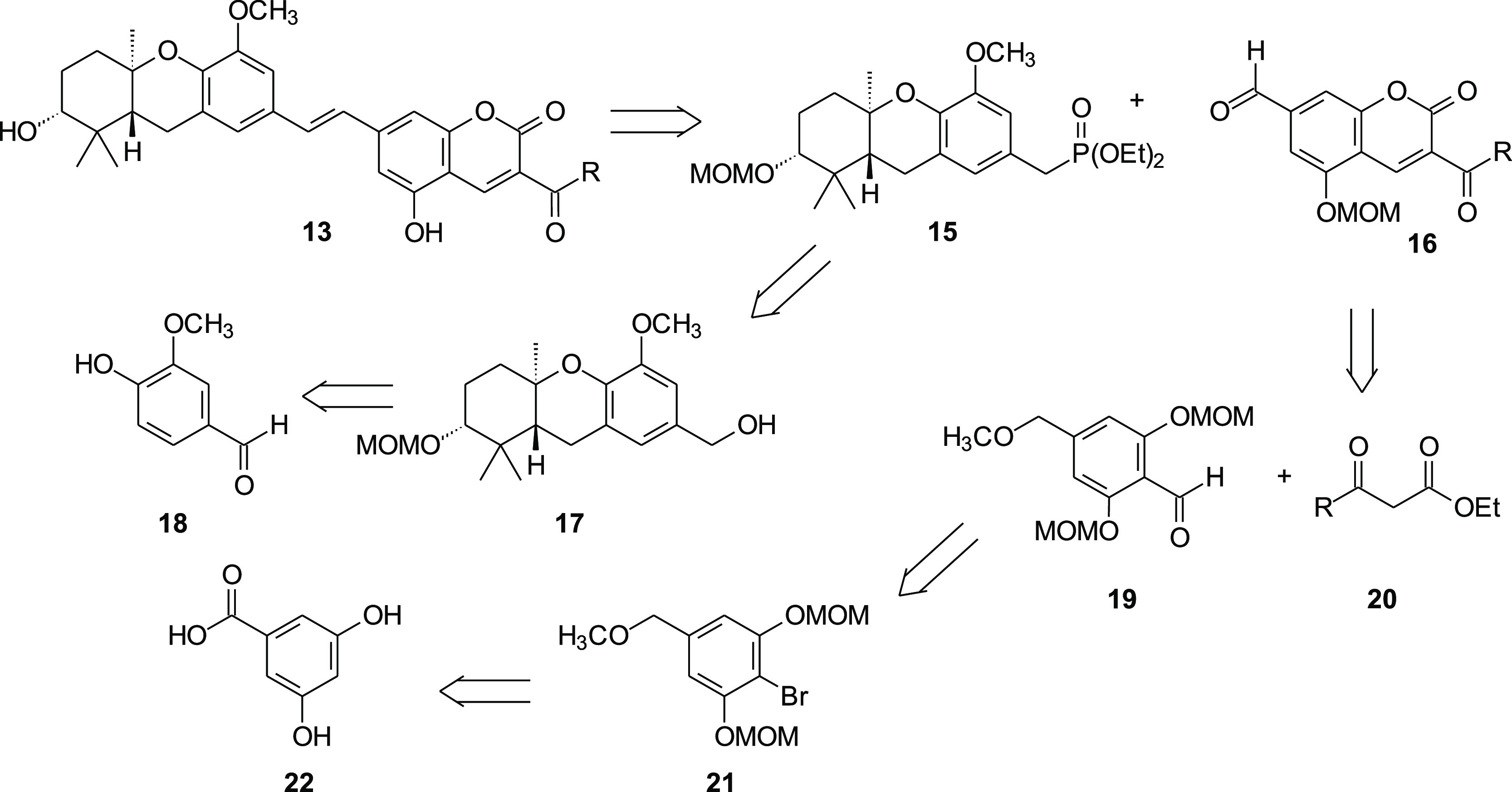
One Retrosynthesis to the Schweinfurthin Analogue **13**

Although several disconnections have been explored for schweinfurthin assembly,^1,22,23^ we have favored use of late-stage Horner–Wadsworth–Emmons (HWE) condensation to form the central stilbene olefin because this approach allows a highly convergent synthesis. From this perspective, the coumarin-containing schweinfurthin F analogue **13** could be seen arising from an HWE olefination between phosphonate **15** and aldehyde **16** (Scheme 1). Phosphonate **15** may be formed from the known tricyclic alcohol **17**,^10^ which can be prepared in high enantiomeric excess from commercial vanillin (**18**) through an enantioselective Shi epoxidation.^24^ If the HWE condensation were postponed to the end of the synthetic sequence, the complementary aldehyde **16** would be required. Coumarin **16** could be prepared via a Knoevenagel condensation between the aldehyde **19** and a β-ketoester of the general structure **20**, with ethyl acetoacetate providing the methyl ketone and extended acetoacetates giving larger analogues. Aldehyde **19** could be seen to arise from bromide **21** by halogen metal exchange followed by reaction with dimethylformamide (DMF). Finally, the benzyl methyl ether **21** could be obtained from commercial 3,5-dihydroxybenzoic acid (**22**).

Initial synthetic efforts were focused on the coumarin component because previous syntheses of other schweinfurthins provided confidence that an appropriate tricyclic component could be prepared. When compounds **13** and **16** include a methyl ketone, this group can be viewed as a mimic of the prenyl group in schweinfurthin F and a homoprenyl ketone could be imagined to mimic the geranyl substituent of larger schweinfurthins. Our efforts began with the prenyl mimic where R is a methyl group because this allowed use of commercially available ethyl acetoacetate as the ketoester **20**.

**Scheme 2:**
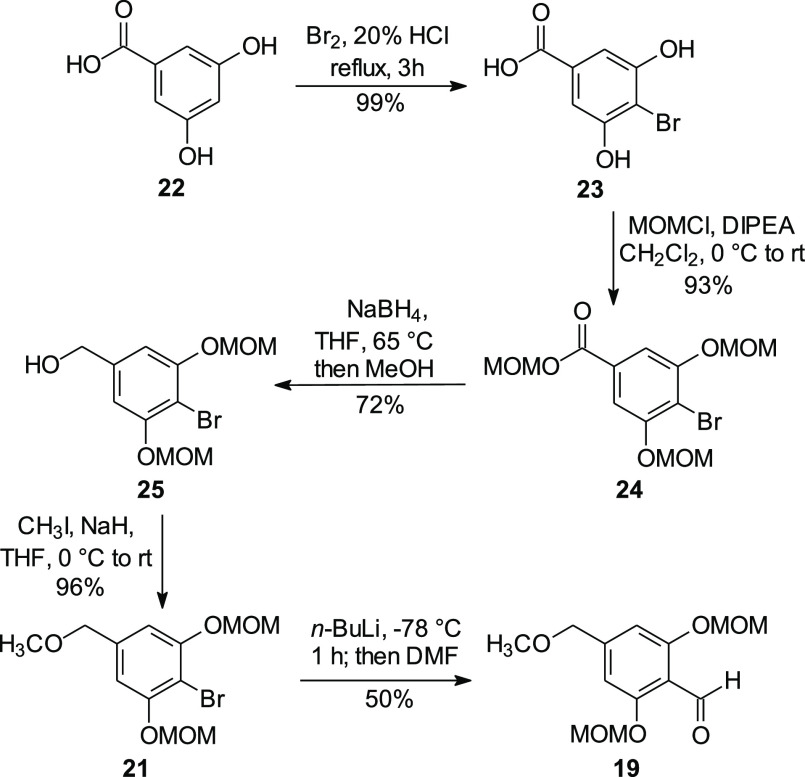
Synthesis of the Aldehyde Intermediate **19**

Although the brominated resorcinol **23** is commercially available, it can be easily prepared in virtually quantitative yield by treatment of 3,5-dihydroxybenzoic acid (**22**) with bromine (Scheme 2). The benzoic acid **23** has been converted to the benzylic alcohol **25** through a three-step sequence via the methyl ester,^25,26^ but it also was possible to accomplish this overall transformation in just two steps by formation of the acyloxyester **24** while concurrently protecting the phenols as MOM ethers, followed by reduction of this intermediate to the desired alcohol **25**. Protection of the benzylic alcohol **25** as the methyl ether **21** proceeded smoothly,^27^ and halogen metal exchange followed by treatment with DMF afforded the desired aldehyde **19**.

**Scheme 3:**
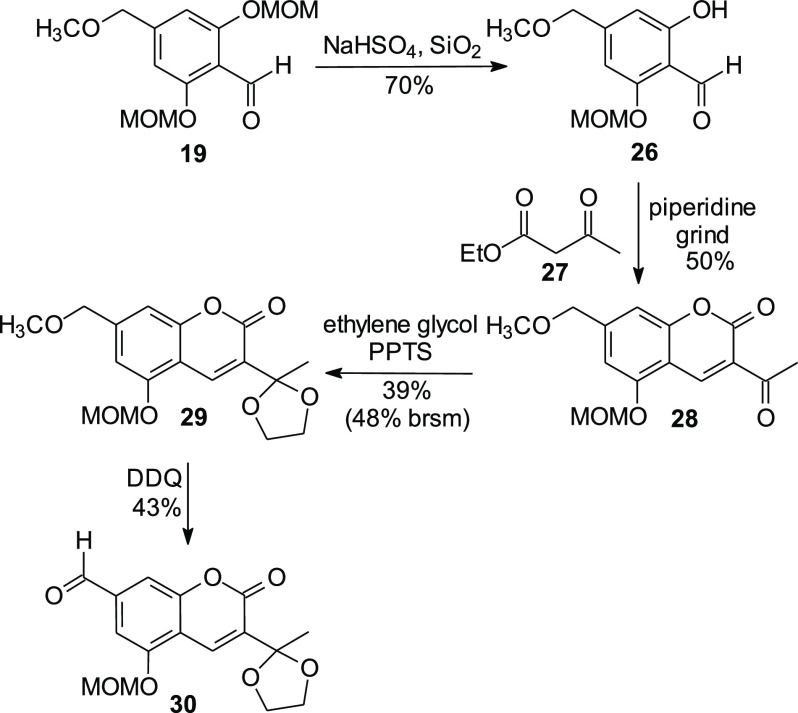
Formation of the Coumarin Aldehyde **30**

With the aldehyde **19** in hand, attention was turned to formation of the desired coumarin ring system through a Knoevenagel condensation. All efforts to form a coumarin directly from the bis-MOM-protected compound **19** went unrewarded. Fortunately, treatment of compound **19** with sodium bisulfate on silica resulted in cleavage of a single MOM protecting group in reasonably good yield (70%, Scheme 3).^28,29^ Grinding the resulting *ortho*-hydroxy benzaldehyde **26** with ethyl acetoacetate (**27**) and piperidine resulted in condensation and cyclization to afford the coumarin **28**. Because ketones can undergo reaction with DDQ via their tautomeric enol forms, the ketone **28** was protected as its acetal **29**. Subsequent reaction with DDQ gave the desired coumarin aldehyde **30** in modest yield. Preparation of the tricyclic phosphonate **15** then was investigated because aldehyde **30** appeared to be an appropriate substrate for an HWE condensation.

**Scheme 4:**
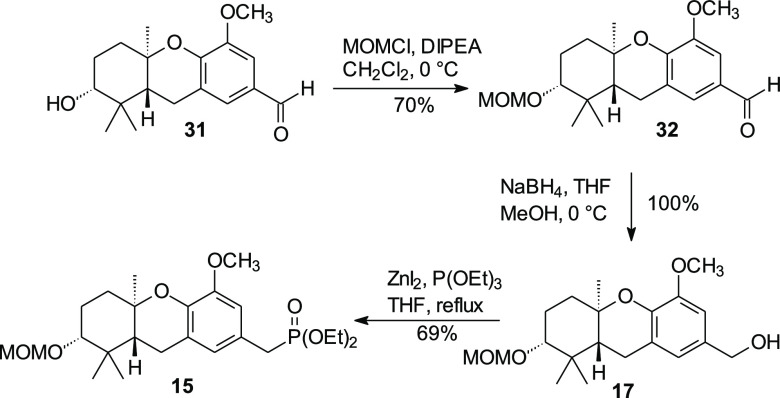
Direct Conversion of Benzylic Alcohol **17** to Phosphonate **15**

The tricyclic phosphonate **15** was employed in our original synthesis of schweinfurthin F,^10^ where it was prepared from the corresponding alcohol **17** in an overall yield of 62% via a classical approach involving formation of the mesylate, displacement by sodium iodide, and an Arbuzov reaction with triethyl phosphite. Instead, a shortened Arbuzov approach was followed. After the aldehyde **31** was prepared via DDQ oxidation of the corresponding benzyl methyl ether, the C-2 alcohol was protected as the MOM ether (**32**) and the aldehyde was reduced to the benzylic alcohol **17** (Scheme 4). Then the alcohol **17** simply was treated with zinc iodide and triethyl phosphite, modernized Arbuzov conditions^30^ for benzylic alcohols, which gave phosphonate **15** in a single step and 69% isolated yield.

**Scheme 5:**
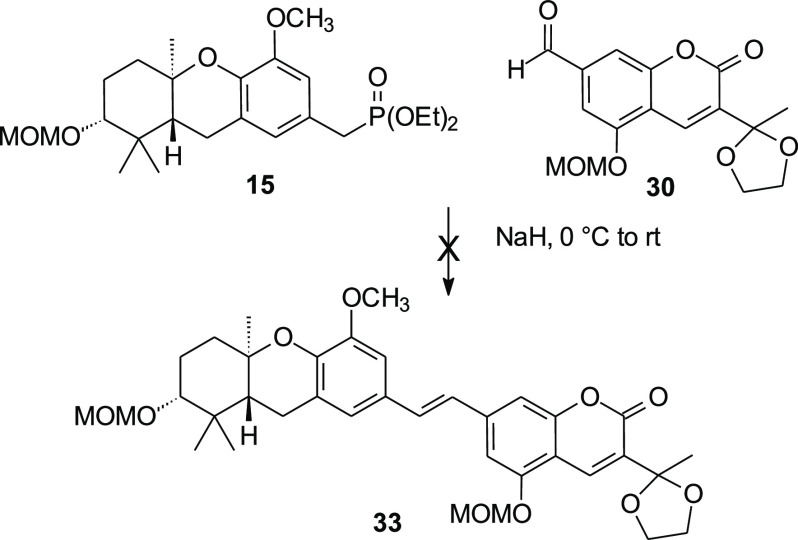
Initially Attempted HWE Condensation

From all perspectives, the HWE condensation of phosphonate **15** and aldehyde **30** was expected to be straightforward, and parallel reactions have been employed in multiple schweinfurthin syntheses. To our disappointment, this specific HWE condensation failed despite repeated attempts (Scheme 5) perhaps because the coumarin subunit was not stable under the reaction conditions. Whatever the root cause, the failure of this reaction necessitated a redesign of the synthetic sequence.

**Scheme 6:**
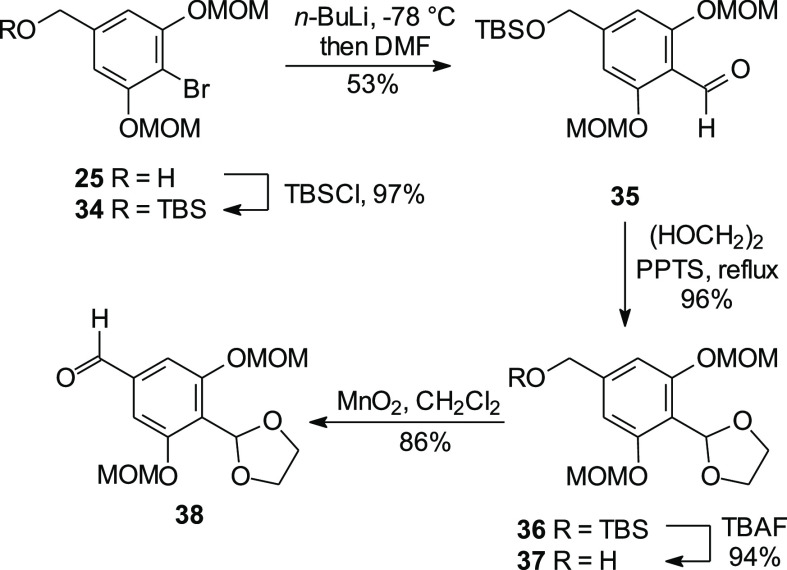
Synthesis of the Aldehyde **38**

To take full advantage of the intermediates in hand and the experience gained with the successful reactions described above, introduction of the coumarin ring system was postponed until after the formation of the central stilbene. Therefore, the alcohol **25** was protected as its TBS ether **34** to avoid potential side-reactions during DDQ oxidation (Scheme 6). Compound **34** undergoes lithium halogen exchange under standard conditions, and a subsequent reaction with DMF gave the aldehyde **35**. After protection of the carbonyl group as its acetal **36**, treatment with TBAF generated the primary alcohol **37**. Final MnO_2_ oxidation provided the new HWE coupling partner, aldehyde **38**.

**Scheme 7:**
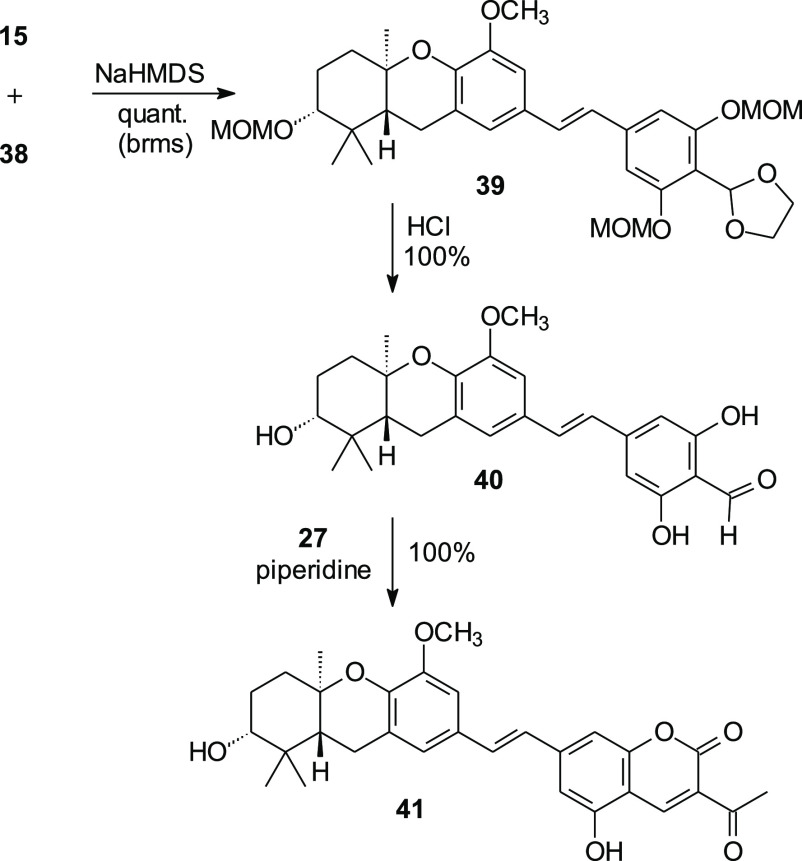
Assembly of the First Coumarin-Based Schweinfurthin (**41**)

The HWE condensation of phosphonate **15** and aldehyde **38** proceeded smoothly upon treatment with sodium hexamethyldisilazide, affording the schweinfurthin analogue **39** in quantitative yield based on recovered phosphonate **15** (Scheme 7). The three MOM acetals and the ethylene glycol-protected aldehyde underwent hydrolysis under acidic conditions to afford the aldehyde **40**. After aldehyde **40** was combined with ethyl acetoacetate (**27**) and piperidine and the reaction was allowed to stir in anhydrous MeOH, the desired fluorescent coumarin schweinfurthin analogue **41** was obtained in high enantiomeric excess.^10^ The spectral data for coumarin **41** indicates that the compound has an absorption maximum at ∼420 nm and an emission maximum at ∼590 nm, showing the expected fluorescence.

**Scheme 8:**
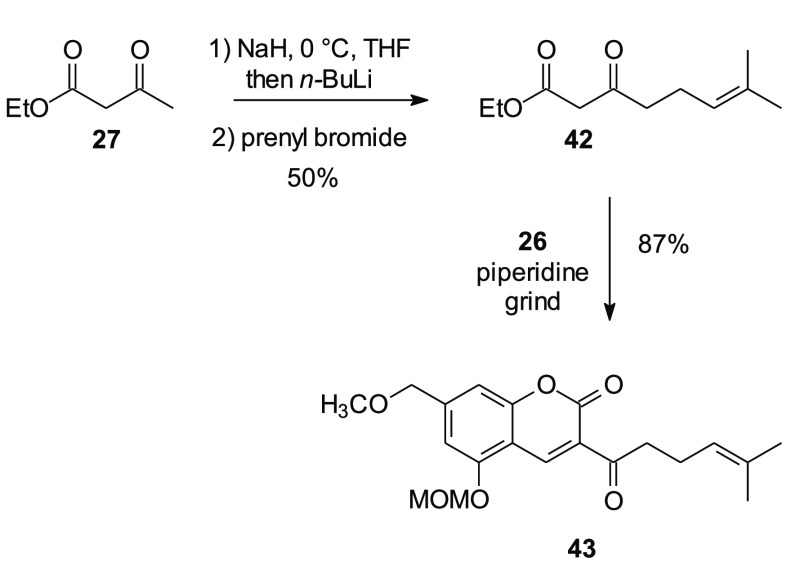
Synthesis of Coumarin **43** with an Extended Isoprenoid Chain

In principal, synthesis of other coumarin-based schweinfurthin analogues could be based on C-alkylation of the methyl ketone in compound **41**. However this would certainly require protection of the free phenol and probably the C-2 hydroxyl group as well. A more attractive approach might involve extension of ethyl acetoacetate (**27**) prior to the Knoevenagel condensation. To test this possibility, the β-ketoester **42** was prepared via alkylation of the ethyl acetoacetate dianion (Scheme 8).^31^ When compound **42** was ground in a mortar with a pestle in the presence of piperidine to induce condensation and cyclization by mechanochemical means, the extended coumarin **43** was obtained in an attractive yield. Although synthesis of compound **43** demonstrates the accessibility of more extended coumarins, pursuit of additional schweinfurthin analogues was postponed pending the results of bioassays on the new analogues in hand.

Both schweinfurthin analogues **40** and **41** were tested in the NCI-60 cell line bioassay. Both compounds were first tested in a single-dose assay and demonstrated sufficient activity to warrant testing in the full five-dose assay. The aldehyde **40** shows modest activity against SF-295 with a GI_50_ of 3.0 μM (Table 1 and Supporting Information). This activity is 270 times less potent than that of natural schweinfurthin A (**1**). Although aldehyde **40** is not as active as most schweinfurthins and schweinfurthin analogues sent to the NCI, it does show a pattern of activity similar to that of other schweinfurthins, with a Pearson correlation coefficient of 0.66 to schweinfurthin A.^32^ The GI_50_ against each cell line in the NCI-60 assay for compound **40** shows a pattern similar to that of other schweinfurthins. Of the cell lines tested, analogue **40** also has the greatest activity against the CNS cancer cell line SF-539 with a GI_50_ of 1.3 μM, which aligns with our expectation that the schweinfurthins have selective activity against CNS malignancies.

**Table 1:** Comparison of the Activity (GI_50_) of Compounds **40** and **41** to Representative Schweinfurthins against Selected CNS Cell Lines

compound # (NSC number)	SF-295 (μM)	SF-539 (μM)	SNB-75 (μM)	Pearson correlation to **1**
**1** (696119)	0.011	0.010	0.015	1.00
**44** (730430)^a^	1.5	–	15.8	0.39
**14** (740545)	0.066	0.28	0.18	0.78
**40** (819974)	3.0	1.3	1.8	0.66
**41** (823234)	0.51	0.98	1.0	0.66

a Figure 4.

**Figure 4:**
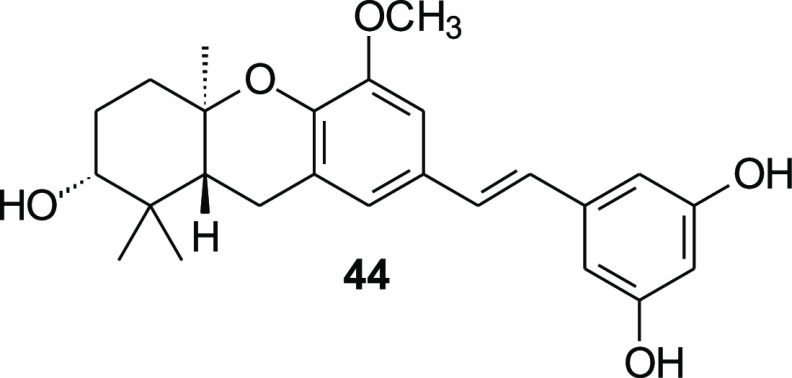
Parent compound **44**.

The five-dose assay of coumarin **41** also shows selective activity toward some cancer cell lines over others (c.f. Supporting Information) in a pattern of activity strikingly similar to that of other schweinfurthins that carry a substituent para to the stilbene linkage (i.e. more substituted variations on the parent compound **44**, Figure 4). Among the most sensitive cell lines were the SF-295 and SF-539 human-derived glioblastoma lines, with GI_50_ values of 0.51 and 0.98 μM respectively, but cells in the leukemia (RPMI-8226, 0.32 μM) and renal (RXF 393, 0.43 μM) panels also were sensitive as is often the case with other schweinfurthins. Conversely, the ovarian cancer panel was uniformly resistant, which is also typical of the schweinfurthins. The three-fold increase in potency of the analogue **41** relative to its precursor, aldehyde **40**, also is striking and encouraging.

In conclusion, two new schweinfurthin analogues, coumarin **41** and its immediate precursor aldehyde **40**, have been synthesized and tested for biological activity in the NCI-60 cell bioassay. Although the initial approach to the central stilbene demonstrated only that this coumarin system did not survive the standard HWE reaction conditions, it proved possible to incorporate the coumarin ring system after formation of the central stilbene. Both traditional Knoevenagel condensation and mechanical grinding of an ortho hydroxy aldehyde with a β-ketoester allowed formation of the coumarin system. Furthermore, the target compound **41** displays both significant antiproliferative activity in the NCI 60-cell line screen and fluorescent properties that may help illuminate the mechanism of action for the schweinfurthins. Finally, the reactions and strategies reported here might be applicable to preparation of fluorescent analogues of other natural stilbenes, including compounds such as combretastatin,^33^ resveratrol^34,35^ and its myriad derivatives,^36^ the chiricanines,^37^ the arachidins and arahypins,^38^ and the pawhuskins.^39^

## Experimental Section

### General Section

Diethyl ether (Et_2_O) and tetrahydrofuran (THF) were distilled from sodium and benzophenone, and dichloromethane (CH_2_Cl_2_) was distilled from calcium hydride prior to use. Solutions of *n*-BuLi were purchased from commercial sources and titrated with diphenylacetic acid to determine molar concentrations prior to use. All other reagents and solvents were purchased from commercial sources and used without further purification. The nuclear magnetic resonance (NMR) spectra were obtained on 300, 400, or 600 MHz Bruker spectrometers with Si(CH_3_)_4_ (^1^H, δ 0.00), CDCl_3_ (^1^H, δ 7.26; ^13^C, δ 77.2), CD_3_CN (^1^H, δ 1.94; ^13^C, δ 118.3, 1.32), or (CD_3_)_2_CO (^1^H, δ 2.05; ^13^C, δ 206.3, 29.8) as internal standards. To assign signals as C, CH, CH_2_, or CH_3_, DEPT-135 NMR spectra were obtained. High-resolution mass spectra were obtained at the University of Iowa Mass Spectrometry Facility. Silica gel (60 Å, 0.040–0.063 mm) was used for flash column chromatography. The UV–vis spectra were obtained on a Cary UV–vis NIR spectrophotometer, and fluorescence data were collected on HORIBA Scientific FluroMax-4. A quartz (200–2500 nm) 1400 μL Hellma Analytics cuvette (semi-micro cell type 114F-QS) with a 10 mm × 4 mm path length fitted with a PTFE stopper was used for UV–vis and fluorometry.

### 4-Bromo-3,5-dihydroxybenzoic Acid (**23**)

To an oven-dried and argon-purged round-bottom flask containing 3,5-dihydroxybenzoic acid (**22**, 10.0 g, 64.5 mmol) was added aqueous 20% HCl (110 mL) followed by a dropwise addition of bromine (3.31 mL, 64.5 mmol). The reaction was heated in an oil bath under reflux for 3 h and then was quenched by addition of ice. The solution was washed with Et_2_O (3 × 50 mL), and the combined organic layers were dried (Na_2_SO_4_) and then filtered through a bed of Celite, and the filtrate was concentrated on a rotary evaporator to afford aryl bromide **23** as an off-white solid (14.9 g, 99%). Both the ^1^H NMR and ^13^C NMR spectra were in agreement with the reported data.^40^

### Methoxymethyl 4-Bromo-3,5-bis(methoxymethoxy)benzoate (**24**)

To an oven-dried and argon-purged round-bottom flask containing the carboxylic acid **23** (400 mg, 1.7 mmol) in CH_2_Cl_2_ (20 mL) at 0 °C was added dropwise DIPEA (1.0 mL, 6.0 mmol) followed by a dropwise addition of MOMCl (520 μL, 6.9 mmol). The reaction was allowed to stir at 0 °C for 2.5 h, and then the reaction was quenched by addition of saturated NH_4_Cl (10 mL) and the organic compounds were extracted into CH_2_Cl_2_ (3 × 20 mL). The combined organic layers were washed with 3 N NaOH (15 mL) and dried (Na_2_SO_4_), and the solids were removed by filtration. The filtrate was concentrated on a rotary evaporator to afford compound **24** as a white solid (0.58 g, 93%): ^1^H NMR (400 MHz, CDCl_3_): δ 7.51 (s, 2H), 5.46 (s, 2H), 5.30 (s, 4H), 3.53 (s, 3H), 3.52 (s, 6H); ^13^C{^1^H} NMR (101 MHz, CDCl_3_): δ 165.3, 154.9 (2C), 130.0, 110.2 (2C), 109.9, 95.1 (2C), 91.2, 57.9, 56.6 (2C). HRMS (ESI) *m*/*z*: [M + Na]^+^ calcd for C_13_H_17_O_7_BrNa, 387.0055; found, 387.0060.

### [4-Bromo-3,5-bis(methoxymethoxy)phenyl]methanol (**25**)

To an oven-dried and argon-purged round-bottom flask containing compound **24** (4.56 g, 12.5 mmol) in THF (60 mL) was slowly added solid NaBH_4_ (4.02 g, 106 mmol), and the reaction was allowed to stir at 65 °C for 15 min. After MeOH (60 mL) was added dropwise, the reaction was heated under reflux in an oil bath for an additional 2 h. After cooling to rt, the reaction was quenched by dropwise addition of saturated NH_4_Cl (50 mL). The organic compounds were extracted into EtOAc, the combined organic layers were dried over Na_2_SO_4_, and the solids were removed by filtration. The filtrate was concentrated on a rotary evaporator to afford the benzylic alcohol **25** as a white solid (2.76 g, 72%): ^1^H NMR (400 MHz, CDCl_3_): δ 6.85 (s, 2H), 5.26 (s, 4H), 4.64 (s, 2H), 3.52 (s, 6H); ^13^C{^1^H} NMR (100 MHz, (CD_3_)_2_CO): δ 154.8 (2C), 143.6, 107.3 (2C), 101.15, 95.4 (2C), 63.3, 55.6 (2C).^25^

### 2-Bromo-1,3-bis(methoxymethoxy)-5-(methoxymethyl)benzene (**21**)

To a solution of the alcohol **25** (8.66 g, 28.2 mmol) in THF (200 mL) was slowly added 60% NaH in oil (1.27 g, 53.0 mmol), and the reaction then was allowed to stir at 0 °C for 5 min. To the solution was added iodomethane (2.19 mL, 35.3 mmol), and the solution was allowed to stir for 2 h. After the reaction was quenched by addition of H_2_O, it was extracted with EtOAc. The combined organic layers were washed with 1 N NaOH, dried over Na_2_SO_4_, and filtered, and the filtrate was concentrated on a rotary evaporator to afford the methyl ether **21** as colorless crystals (8.71 g, 96%): ^1^H NMR (400 MHz, (CD_3_)_2_CO): δ 6.88 (s, 2H), 5.30 (s, 4H), 4.40 (s, 2H), 3.49 (s, 6H), 3.36 (s, 3H); ^13^C{^1^H} NMR (100 MHz, (CD_3_)_2_CO): δ 154.9 (2 C), 152.0 (C), 139.8 (C), 108.0 (2 CH), 94.9 (2 CH_2_), 73.5 (CH_2_), 57.4 (CH_3_), 55.6 (2 CH_3_).^27^

### 2,6-Bis(methoxymethoxy)-4-(methoxymethyl)benzaldehyde (**19**)

To a flame-dried and argon-purged round-bottom flask containing *n*-BuLi (2.48 M, 5.5 mL, 13 mmol) at −78 °C was added a −78 °C solution of the aryl bromide **21** (3.55 g, 11.1 mmol) in Et_2_O (200 mL) using a cannula. The solution was allowed to stir for 15 min and then DMF (0.94 mL, 12 mmol) was added dropwise. Once the reaction reached rt, it was quenched by addition of NH_4_Cl and extracted with Et_2_O. The combined organic layers were dried over Na_2_SO_4_ and filtered, and the filtrate was concentrated on a rotary evaporator to afford a yellow oil. Final purification was achieved using an ISCO auto-column (0%–100% EtOAc in hexanes) to afford aldehyde **19** as a yellow oil (1.52 g, 50%): ^1^H NMR (400 MHz, CDCl_3_): δ 10.51 (s, 1H), 6.82 (s, 2H), 5.27 (s, 4H), 4.42 (s, 2H), 3.50 (s, 6H), 3.41 (s, 3H); ^13^C{^1^H} NMR (100 MHz, CDCl_3_): δ 188.5 (CH), 159.4 (2 CH), 147.1 (C), 114.9 (C), 106.6 (2 C), 94.6 (2 CH_2_), 73.8 (CH_2_), 58.3 (CH_3_), 56.3 (2 CH_3_). HRMS (ESI) *m*/*z*: [M + Na]^+^ calcd for C_13_H_18_O_6_Na, 293.0996; found, 293.0995.

### 2-Hydroxy-6-(methoxymethoxy)-4-(methoxymethyl)benzaldehyde (**26**)

A mixture of compound **19** (1.38 g, 5.09 mmol) and NaHSO_4_ on SiO_2_ (3.06 g)^28,29^ was allowed to stir in CH_2_Cl_2_ (75 mL) at rt for 10 min. The mixture was filtered through Celite, washed with CH_2_Cl_2_, and the filtrate was concentrated on a rotary evaporator to afford the phenol **26** (806 mg, 70%): ^1^H NMR (300 MHz, CDCl_3_): δ 11.85 (s, 1H), 10.23 (s, 1H), 6.51 (s, 1H), 6.44 (s, 1H), 5.23 (s, 2H), 4.31 (s, 2H), 3.43 (s, 3H), 3.32 (s, 3H); ^13^C{^1^H} NMR (75 MHz, CDCl_3_): δ 193.7 (CH), 163.3 (C), 160.0 (C), 150.3 (C), 110.3 (C), 108.4 (CH), 102.2 (CH), 94.5 (CH_2_), 73.7 (CH_2_), 58.3 (CH_3_), 56.4 (CH_3_). HRMS (ESI) *m*/*z*: [M + H]^+^ calcd for C_11_H_15_O_5_, 227.0920; found, 227.0913.

### 3-Acetyl-5-(methoxymethoxy)-7-(methoxymethyl)chromen-2-one (**28**)

The aldehyde **26** (515 mg, 2.28 mmol) was combined with ethyl acetoacetate (**27**, 580 μL, 4.55 mmol) and piperidine (nine drops) in a mortar, and the solution was ground with a pestle for 30 min. The residue was transferred to a round-bottom flask with EtOAc, and the solvent was removed using a rotary evaporator. Final purification by crystallization from hot EtOH and H_2_O gave the coumarin **28** (329 mg, 50%): ^1^H NMR (300 MHz, CDCl_3_): δ 8.87 (s, 1H), 6.96 (s, 1H), 6.93 (s, 1H), 5.33 (s, 2H), 4.50 (s, 2H), 3.51 (s, 3H), 3.44 (s, 3H), 2.70 (s, 3H); ^13^C{^1^H} NMR (75 MHz, CDCl_3_): δ 195.3 (C), 159.6 (C), 157.2 (C), 156.5 (C), 149.1 (C), 142.2 (CH), 123.6 (C), 109.9 (C), 108.1 (CH), 108.0 (CH), 95.1 (CH_2_), 74.3 (CH_2_), 59.0 (CH_3_), 57.0 (CH_3_), 30.4 (CH_3_). HRMS (ESI) *m*/*z*: [M + H]^+^ calcd for C_15_H_17_O_6_, 293.1020; found, 293.1019.

### 5-(Methoxymethoxy)-7-(methoxymethyl)-3-(2-methyl-1,3-dioxolan-2-yl)chromen-2-one (**29**)

To a round-bottom flask containing the ketone **28** (250 mg, 850 μmol) in benzene (20 mL) were added ethylene glycol (0.34 mL, 6.0 mmol) and pyridinium *p*-toluenesulfonate (43 mg, 0.17 mmol). The solution was heated in an oil bath under reflux with a Dean–Stark trap in place for 18 h. After it had cooled to rt, the reaction was quenched by addition of saturated NaHCO_3_ (5 mL) and extracted with EtOAc, and the combined organic layers were dried (Na_2_SO_4_) and filtered. The filtrate was concentrated by rotary evaporation. Final purification was achieved through flash column chromatography (20% EtOAc in hexanes) to afford the acetal **29** as colorless crystals (110 mg, 39%) and recovered ketone **28** (44 mg, 18%). For the acetal: ^1^H NMR (400 MHz, CDCl_3_): δ 8.27 (s, 1H), 6.96 (s, 1H), 6.93 (s, 1H), 5.33 (s, 2H), 4.40 (s, 2H), 4.10 (m, 2H), 3.92 (m, 2H), 3.55 (s, 3H), 3.44 (s, 3H), 1.83 (s, 3H); ^13^C{^1^H} NMR (100 MHz, CDCl_3_): δ 159.4 (C), 154.9 (C), 154.2 (C), 143.8 (C), 133.6 (CH), 126.7 (C), 109.0 (C), 108.1 (CH), 107.2 (CH), 107.1 (C), 94.9 (CH_2_), 74.0 (CH_2_), 65.0 (2 CH_2_), 58.5 (CH_3_), 56.6 (CH_3_), 24.4 (CH_3_). HRMS (ESI) *m*/*z*: [M + H]^+^ calcd for C_17_H_21_O_7_, 337.1282; found, 337.1278.

### 5-(Methoxymethoxy)-7-methyl-3-(2-methyl-1,3-dioxolan-2-yl)chromen-2-one (**30**)

To a solution of the methyl ether **29** (110 mg, 330 μmol), CH_2_Cl_2_ (5 mL), and H_2_O (0.5 mL) was added DDQ (210 mg, 900 μmol), and the mixture was allowed to stir at rt for 24 h. The mixture was quenched by addition of saturated NaHCO_3_, extracted with CH_2_Cl_2_, and the combined organic layers were washed with brine, dried (Na_2_SO_4_), and filtered. The filtrate was concentrated on a rotary evaporator. Final purification was achieved through flash column chromatography (20% EtOAc in hexanes) to afford the aldehyde **30** as a yellow oil (45 mg, 43%): ^1^H NMR (400 MHz, CDCl_3_): δ 10.00 (s, 1H), 8.29 (s, 1H), 7.48 (s, 1H), 7.43 (s, 1H), 5.38 (s, 2H), 4.12 (m, 2H), 3.95 (m, 2H), 3.53 (s, 3H), 1.82 (s, 3H); ^13^C{^1^H} NMR (100 MHz, CDCl_3_): δ 190.7 (CH), 158.5 (C), 154.8 (C), 154.7 (C), 138.8 (C), 132.7 (CH), 129.8 (C), 118.1 (C), 111.9 (CH), 107.4 (CH), 107.0 (C), 95.1 (CH_2_), 65.1 (2 CH_2_), 56.8 (CH_3_), 24.5 (CH_3_). HRMS (ESI) *m*/*z*: [M + H]^+^ calcd for C_16_H_16_O_7_Na, 343.0788; found, 343.0796.

### (7*R*,8a*R*,10a*R*)-4-Methoxy-7-(methoxymethoxy)-8,8,10a-trimethyl-6,7,8*a*,9-tetrahydro-5*H*-xanthene-2-carbaldehyde (**32**)

To a vial containing aldehyde **31**(8) (177 mg, 580 μmol) in CH_2_Cl_2_ (5 mL) at 0 °C was added dropwise DIPEA (110 μL, 0.64 mmol). The solution was allowed to stir for 10 min, and then MOMCl (50 μL, 0.64 mmol) was added dropwise. After the solution was allowed to stir for 72 h, it was quenched by addition of NH_4_Cl and was extracted with CH_2_Cl_2_. The combined organic layers were washed with 1 N NaOH, dried (Na_2_SO_4_), and filtered, and the filtrate was concentrated in vacuo to afford aldehyde **32** (140 mg, 70%): ^1^H NMR (400 MHz, CDCl_3_): δ 9.80 (s, 1H), 7.26–7.23 (m, 2H), 4.79 (d, *J* = 6.9 Hz, 1H), 4.66 (d, *J* = 7.0 Hz, 1H), 3.90 (s, 3H), 3.41 (s, 3H), 3.31–3.24 (m, 1H), 2.80–2.78 (m, 2H), 2.18 (td, *J* = 12.9, 3.6 Hz, 1H), 2.02 (dq, *J* = 14.4, 4.0 Hz, 1H), 1.82 (ddd, *J* = 13.8, 13.8, 4.1 Hz, 1H), 1.72 (dd, *J* = 11.3, 7.5 Hz, 1H), 1.65–1.58 (m, 1H), 1.28 (s, 3H), 1.10 (s, 3H), 0.92 (s, 3H); ^13^C NMR (101 MHz, CDCl_3_): δ 191.2, 149.6, 148.9, 129.0, 127.4, 122.6, 107.4, 96.3, 83.8, 78.5, 56.1, 55.7, 46.7, 38.4, 37.5, 27.5, 25.4, 23.1, 20.1, 15.2. HRMS (ESI) *m*/*z*: [M + H]^+^ calcd for C_20_H_29_O_5_, 349.2015; found, 349.2006.

### [(7*R*,8a*R*,10a*R*)-4-Methoxy-7-(methoxymethoxy)-8,8,10a-trimethyl-6,7,8a,9-tetrahydro-5*H*-xanthen-2-yl]methanol (**17**)

To an oven-dried and argon-purged round-bottom flask containing the aldehyde **32** (350 mg, 1.00 mmol) in THF (10 mL) and MeOH (2 mL) at 0 °C was added solid NaBH_4_ (234 mg, 1.60 mmol). The solution was allowed to stir for 40 min, was quenched by addition of H_2_O, and then was extracted with EtOAc. The combined organic layers were washed with saturated NaHCO_3_ and brine and dried (MgSO_4_). After filtration, the filtrate was concentrated on a rotary evaporator to afford the benzylic alcohol **17** (352 mg, 100%) as a colorless oil. Both the ^1^H and ^13^C NMR spectra were in agreement with data reported in the literature.^10^

### [(2*R*,4*aR*,9*aR*)-7-(Diethoxyphosphonylmethyl)-5-methoxy-1,1,4*a*-trimethyl-3,4,9,9*a*-tetrahydro-2*H*-xanthen-2-yl]oxymethanol (**15**)

To an oven-dried and argon-purged round-bottom flask containing ZnI_2_ (1.1 g, 3.5 mmol) and triethyl phosphite (400 μL, 2.3 mmol) in THF (15 mL) was added benzylic alcohol **17** (410 mg, 1.2 mmol). The reaction was heated in an oil bath under reflux for 17 h. The solution was concentrated in vacuo to 1 mL, and then the residue was dissolved in Et_2_O, which caused formation of a solid that was removed by filtration. After the filtrate was washed with 1 N NaOH (0.5 mL), the organic layer was dried (Na_2_SO_4_) and filtered, and the filtrate was concentrated on a rotary evaporator. Excess triethyl phosphite was removed using high vacuum to afford phosphonate **15** (370 mg, 69%) as a colorless oil. Both the ^1^H and ^31^P NMR spectra were in agreement with data reported for compound **15** prepared by a traditional Arbuzov sequence.^10^

### Attempted Preparation of 7-[(*E*)-2-[(7*R*,8a*R*,10a*R*)-4-Methoxy-7-(methoxymethoxy)-8,8,10a-trimethyl-6,7,8a,9-tetrahydro-5*H*-xanthen-2-yl]vinyl]-3-(2-methyl-1,3-dioxolan-2-yl)chromen-2-one (**33**)

To a flame-dried round-bottom flask containing the phosphonate **15** (78 mg, 0.17 mmol) in THF (1 mL) at 0 °C was added NaH (60% dispersion in oil, 10 mg, 0.25 mmol). To the stirring solution was added the aldehyde **30** (8.6 mg, 26 μmol) in THF (2 mL). The reaction was allowed to warm to rt naturally and then was quenched by addition of H_2_O. The organic compounds were extracted into EtOAc, dried (Na_2_SO_4_), and filtered, and the filtrate was concentrated *in vacuo*. The desired stilbene **33** could not be detected in the resulting material.

### [4-Bromo-3,5-bis(methoxymethoxy)phenyl]methoxy *tert*-Butyldimethylsilane (**34**)

To an oven-dried and argon-purged round-bottom flask containing alcohol **25** (1.3 g, 3.4 mmol) in anhydrous CH_2_Cl_2_ (200 mL) was added imidazole (470 mg, 6.9 mmol) followed by TBSCl (560 mg, 3.8 mmol), and the reaction was allowed to stir at rt for 14 h. After the reaction was quenched by addition of water, the aqueous layer was extracted with CH_2_Cl_2_. The combined organic layers were washed with brine, dried (Na_2_SO_4_), and filtered through a pad of Celite, and the filtrate was concentrated under reduced pressure to afford the silyl ether **34** as a yellow oil (1.4 g, 97%): ^1^H NMR (400 MHz, CDCl_3_): δ 6.84 (s, 2H), 5.24 (s, 4H), 4.68 (s, 2H), 3.52 (s, 6H), 0.94 (s, 9H), 0.10 (s, 6H). ^13^C{^1^H} NMR (75 MHz, CDCl_3_): δ 158.5 (2 C), 144.4 (C), 107.3 (2 CH), 103.5 (C), 94.7 (2 CH_2_), 64.9 (CH_2_), 56.1 (2 CH_3_), 26.1 (3 CH_3_), 18.6 (C), −5.1 (2 CH_3_).^41^

### 4-(((*tert*-Butyldimethylsilyl)oxy)methyl)-2,6-bis(methoxymethoxy)benzaldehyde (**35**)

An oven-dried round-bottom flask containing aryl bromide **34** (5.6 g, 13 mmol) in Et_2_O (150 mL) was cooled to −78 °C for 20 min. To the solution was added *n*-BuLi (7.4 mL, 18 mmol, 2.4 M). Immediately after the addition was complete, anhydrous DMF (1.4 mL, 18 mmol) was added dropwise and the reaction was allowed to stir and warm to rt overnight. After the reaction was quenched by addition of saturated NH_4_Cl (50 mL), the organic compounds were extracted into Et_2_O (3 × 50 mL). The combined organic layers were dried (Na_2_SO_4_), the solids were removed by filtration, and the filtrate was concentrated on a rotary evaporator. Final purification was achieved by ISCO normal-phase auto-chromatography (0–5% EtOAc in hexanes), which gave aldehyde **35** as a yellow oil (2.61 g, 53%). Both the ^1^H and ^13^C NMR spectra match the literature data for material prepared by a different route.^42^^1^H NMR (300 MHz, CDCl_3_): δ 10.34 (s, 1H), 6.70 (s, 2H), 5.09 (s, 4H), 4.56 (s, 2H), 3.32 (s, 6H), 0.80 (s, 9H), 0.05 (s, 6H).

### *tert*-Butyl-[[4-(1,3-dioxolan-2-yl)-3,5-bis(methoxymethoxy)phenyl]methoxy]dimethylsilane (**36**)

A round-bottom flask containing aldehyde **35** (680 mg, 1.8 mmol), ethylene glycol (660 μL, 11 mmol), and PPTS (110 mg, 370 μmol) in benzene (50 mL) was fitted with a Dean–Stark trap and heated in an oil bath under reflux. The reaction progress was monitored periodically by thin-layer chromatography. After 1 h, the reaction was allowed to cool to rt, quenched by addition of saturated NaHCO_3_ (5 mL), and extracted with EtOAc (3 × 20 mL). The combined organic layers were dried (Na_2_SO_4_), the solid was removed by gravity filtration, and the resulting filtrate was concentrated on a rotary evaporator to afford acetal **36** (720 mg, 96%): ^1^H NMR (400 MHz, CDCl_3_) 6.74 (2H, s), 6.40 (1H, s), 5.09 (4H, s), 4.63 (2H, s), 4.11 (2H, m), 3.89 (2H, m), 3.39 (6H, s), 0.89 (9H, s), 0.03 (6H, s); ^13^C NMR (101 MHz, CDCl_3_) 157.4 (C), 145.0 (C), 114.1 (2 CH), 106.5 (CH), 98.9 (2 C), 94.7 (2 CH_2_), 65.6 (2 CH_2_), 64.4 (CH_2_), 56.1 (2 CH_3_), 26.2 (3 CH_3_), 18.4 (C), −5.4 (2 CH_3_). HRMS (ESI) *m*/*z*: [M + H]^+^ calcd for C_20_H_35_O_7_Si, 417.2147; found, 415.2145.

### [4-(1,3-Dioxolan-2-yl)-3,5-bis(methoxymethoxy)phenyl]methanol (**37**)

To a flask containing the silyl ether **36** (2.5 g, 6.0 mmol) in THF (150 mL) was added TBAF (1.0 M in THF, 6.0 mL, 6.0 mmol), and the solution was allowed to stir at 0 °C and naturally warm to rt over 1 h. The reaction was quenched by addition of water, and the organic compounds were extracted into EtOAc (3 × 100 mL), washed with brine, and dried (Na_2_SO_4_). The solids were removed by gravity filtration, and the filtrate was concentrated on a rotary evaporator to afford alcohol **37** as a pale yellow solid (1.7 g, 94%): ^1^H NMR (400 MHz, CDCl_3_) 6.81 (2H, s), 6.48 (1H, s), 5.20 (4H, s), 4.57 (2H, s), 4.24–4.19 (2H, m), 4.04–4.01 (2H, m), 3.50 (6H, s); ^13^C NMR (101 MHz, CDCl_3_): δ 157.3 (2 C), 144.2 (C), 114.8 (C), 106.9 (2 CH), 98.5 (CH), 94.7 (2 CH_2_), 66.1 (2 CH_2_), 65.4 (CH_2_), 56.3 (2 CH_3_). HRMS (ESI) *m*/*z*: [M + H]^+^ calcd for C_14_H_21_O_7_, 301.1282; found, 301.1280.

### 4-(1,3-Dioxolan-2-yl)-3,5-bis(methoxymethoxy)benzaldehyde (**38**)

To a flame-dried and argon-purged round-bottom flask containing alcohol **37** (210 mg, 690 μmol) in anhydrous CH_2_Cl_2_ (20 mL) was slowly added manganese dioxide (1.5 g, 14 mmol). The resulting mixture was allowed to stir at rt for 26 h. The mixture then was filtered through a bed of Celite, and the filtrate was concentrated to afford aldehyde **38** (180 mg, 86%): ^1^H NMR (400 MHz, CDCl_3_): δ 9.89 (s, 1H), 7.29 (s, 2H), 6.50 (s, 1H), 5.24 (s, 4H), 4.23–4.20 (m, 2H), 4.04–4.00 (m, 2H), 3.49 (s, 6H); ^13^C NMR (101 MHz, CDCl_3_): δ 191.7 (CH), 158.0 (2 C), 138.1 (C), 121.5 (C), 109.7 (2 CH), 98.1 (CH), 94.5 (2 CH_2_), 66.5 (2 CH_2_), 56.6 (2 CH_3_). HRMS (ESI) *m*/*z*: [M + H]^+^ calcd for C_14_H_19_O_7_, 299.1125; found, 299.1127.

### (2*R*,4a*R*,9a*R*)-7-[(*E*)-2-[4-(1,3-Dioxolan-2-yl)-3,5-bis(methoxymethoxy)phenyl]vinyl]-5-methoxy-2-(methoxymethoxy)-1,1,4a-trimethyl-3,4,9,9a-tetrahydro-2*H*-xanthene (**39**)

To a flame-dried and argon-purged round-bottom flask containing the phosphonate **15** (116 mg, 250 μmol) and the aldehyde **38** (61 mg, 0.21 mmol) in THF (12 mL) at 0 °C was added dropwise NaHMDS (1.0 M in THF, 680 μL, 680 μmol), and the mixture was allowed to stir at 0 °C for 32 h. The reaction was quenched by a dropwise addition of saturated NH_4_Cl to reach a pH of 8, and the organic compounds were extracted into EtOAc (3 × 20 mL). The combined organic layers were washed with brine and dried (MgSO_4_), the solids were removed by filtration, and the filtrate was concentrated on a rotary evaporator. Final purification was achieved by column chromatography (15–100% EtOAc in hexanes) to afford the recovered phosphonate **15** (98 mg, 83%) and the desired stilbene **39** as a fluorescent yellow oil (25 mg, 16%): ^1^H NMR (400 MHz, CDCl_3_): δ 6.98 (d, *J* = 14.8 Hz, 1H), 6.92 (s, 2H), 6.90–6.84 (m, 3H), 6.47 (s, 1H), 5.22 (s, 4H), 4.77 (d, *J* = 7.1 Hz, 1H), 4.65 (d, *J* = 6.4 Hz, 1H), 4.24–4.19 (m, 2H), 4.02–3.99 (m, 2H), 3.89 (s, 3H), 3.51 (s, 6H), 3.41 (s, 3H), 3.28 (dd, *J* = 11.3, 3.5 Hz, 1H), 2.71 (d, *J* = 8.6 Hz, 1H), 2.15 (d, *J* = 12.9 Hz, 1H), 2.02–1.92 (m, 1H), 1.84–1.75 (m, 1H), 1.75–1.69 (m, 1H), 1.65–1.54 (m, 2H), 1.25 (s, 3H), 1.10 (s, 3H), 0.91 (s, 3H); ^13^C NMR (101 MHz, CDCl_3_): δ 157.5 (2C), 149.3, 142.9, 140.6, 130.1, 126.1, 122.7, 121.1, 114.8, 109.7, 107.0, 106.8 (2C), 98.8, 96.5, 94.9 (2C), 84.1, 66.0 (2C), 56.3, 56.0, 55.6, 47.0, 38.3, 37.6, 29.8, 27.5, 25.4, 23.1, 19.9, 15.2, 14.2. HRMS (ESI) *m*/*z*: [M + H]^+^ calcd for C_34_H_47_O_17_, 615.3164; found, 615.3171.

### 4-[(*E*)-2-[(7*R*,8a*R*,10a*R*)-7-Hydroxy-4-methoxy-8,8,10a-trimethyl-6,7,8a,9-tetrahydro-5*H*-xanthen-2-yl]vinyl]-2,6-dihydroxybenzaldehyde (**40**)

To the acetal **39** (35 mg, 57 μmol) was added 5 M HCl (5 mL) and anhydrous MeOH (15 mL); the solution was allowed to stir at rt for 69 h, and then was quenched by addition of saturated NaHCO_3_ to a pH of 7. The organic compounds were extracted into EtOAc (2 × 25 mL), the combined organic layers were dried (MgSO_4_), and the solids were removed by filtration. The filtrate was concentrated to afford the aldehyde **40** as an orange solid (26 mg, 100%): ^1^H NMR (400 MHz, CDCl_3_): δ 10.27 (s, 1H), 7.05 (d, *J* = 15.9 Hz, 1H), 6.85 (m, 2H), 6.76 (d, *J* = 15.4 Hz, 1H), 6.47 (s, 2H), 3.87 (s, 3H), 3.44 (d, *J* = 6.5 Hz, 1H), 2.71 (d, *J* = 8.6 Hz, 2H), 2.18–2.07 (m, 2H), 1.92–1.80 (m, 1H), 1.73–1.49 (m, 2H), 1.24 (s, 3H), 1.09 (s, 3H), 0.87 (s, 3H); ^13^C NMR (101 MHz, CDCl_3_): δ 193.1 (CH), 149.1 (2 C), 148.1, 143.7, 133.2, 128.0, 125.1 (2 C), 123.0, 121.7, 109.5, 107.4, 105.2, 78.4, 77.5, 56.1, 46.8, 38.5, 37.6, 29.7, 28.2, 27.3, 23.1, 19.9, 14.3. HRMS (ESI) *m*/*z*: [M – H]^–^ calcd for C_26_H_29_O_6_, 437.1964; found, 437.1965.

### 7-[(*E*)-2-[(7*R*,8a*R*,10a*R*)-7-Hydroxy-4-methoxy-8,8,10a-trimethyl-6,7,8a,9-tetrahydro-5*H*-xanthen-2-yl]vinyl]-3-acetyl-5-hydroxychromen-2-one (**41**)

To a flame-dried round-bottom flask containing the aldehyde **40** (19 mg, 41 μmol) in anhydrous MeOH (2 mL) were added ethyl acetoacetate (**27**, 5.2 μL, 41 μmol) and piperidine (2.0 μL, 20 μmol) and the sides of the flask were washed with 1 mL of anhydrous MeOH. The solution was allowed to stir in a foil-covered flask at rt for 75 h, and then the reaction was quenched by addition of H_2_O (10 mL). The organic compounds were extracted into CH_2_Cl_2_ (3 × 20 mL), the combined organic layers were dried (Na_2_SO_4_), and the solids were removed by filtration. The filtrate was concentrated on a rotary evaporator, and the resulting material was purified by column chromatography (50–100% EtOAc in hexanes). Final purification was achieved by washing the solid with pentane (3 × 2 mL) to afford coumarin **41** as a fluorescent orange solid (22 mg, 100%): ^1^H NMR (400 MHz, CD_3_CN): δ 8.66 (s, 1H), 7.27 (d, *J* = 15.9 Hz, 1H), 7.07–7.01 (m, 3H), 6.94 (s, 2H), 3.86 (s, 3H), 3.71–3.67 (m, 1H), 2.76 (d, *J* = 8.8 Hz, 2H), 2.61 (s, 3H), 1.80–1.71 (m, 3H), 1.71–1.65 (m, 2H), 1.22 (s, 3H), 1.09 (s, 3H), 0.87 (s, 3H); ^13^C NMR (151 MHz, CD_3_CN): δ 195.3, 169.0, 166.5, 160.8, 159.1, 156.2, 149.4, 148.9, 141.7, 133.2, 124.3, 122.8, 121.9, 121.1, 108.1, 108.0, 107.7, 104.4, 77.1, 76.9, 55.2, 46.5, 38.2, 37.5, 29.2, 26.7, 25.9, 23.4, 19.3, 13.9. HRMS (ESI) *m*/*z*: [M + Na]^+^ calcd for C_30_H_32_O_7_Na, 527.2046; found, 527.2051.

### Ethyl 7-Methyl-3-oxo-6-octenoate (**42**)

To an oven-dried and argon-purged round-bottom flask containing NaH (2.08 g, 51.9 mmol) in THF (400 mL) at 0 °C was added ethyl acetoacetate (**27**, 6.02 mL, 47.2 mmol), and the solution was stirred for 10 min. To the reaction flask was added dropwise *n*-BuLi (21.6 mL, 51.9 mmol) followed by a dropwise addition of prenyl bromide (6.00 mL, 51.9 mmol). The reaction was stirred at rt for 20 min and then quenched by addition of saturated NH_4_Cl and extracted with Et_2_O. The combined organic layers were dried (Na_2_SO_4_) and filtered, and the filtrate was concentrated on a rotary evaporator. Final purification was achieved by column chromatography (10% EtOAc in hexanes) to afford the β-ketoester **42** as a pale yellow oil (4.69 g, 50%)^.^ Both the ^1^H and ^13^C NMR spectra matched those in the literature.^43^

### 5-(Methoxymethoxy)-7-(methoxymethyl)-3-(5-methylhex-4-enoyl)chromen-2-one (**43**)

To a mortar were added aldehyde **26** (806 mg, 3.56 mmol), the β-ketoester **42** (1.41 g, 7.12 mmol), and piperidine (three drops). The solution was ground with a pestle intermittently over 12 h. Purification was achieved by column chromatography (20–40% EtOAc in hexanes). The desired fractions were combined and heated (oil bath) under reflux in benzene with a Dean–Stark trap overnight. After cooling to rt, the resulting solution was concentrated in vacuo to afford compound **43** (1.12 g, 87%): ^1^H NMR (400 MHz, CDCl_3_): δ 8.83 (s, 1H), 6.93 (s, 1H), 6.90 (s, 1H), 5.30 (s, 2H), 5.13 (tt, *J* = 7.2, 1.3 Hz, 1H) 4.47 (s, 2H), 3.49 (s, 3H), 3.41 (s, 3H), 3.12 (t, *J* = 7.2 Hz, 2H), 2.34 (q, *J* = 7.2 Hz, 2H), 1.64 (s, 3H), 1.60 (s, 3H); ^13^C{^1^H} NMR (100 MHz, CDCl_3_): δ 197.7 (C), 159.2 (C), 156.1 (C), 155.6 (C), 147.5 (CH), 142.8 (C), 132.5 (C), 123.0 (CH), 122.3 (C), 109.0 (C), 107.6 (CH), 107.0 (CH), 94.7 (CH_2_), 73.8 (CH_2_), 58.7 (CH_3_), 56.7 (CH_3_), 42.6 (CH_2_), 25.7 (CH_3_), 22.6 (CH_2_), 17.7 (CH_3_). HRMS (ESI) *m*/*z*: [M + H]^+^ calcd for C_20_H_25_O_6_, 361.1646; found, 361.1624.

## Supporting Information Available

^1^H and ^13^C NMR spectra for all new compounds and full tables of the bioassay data (PDF)
