# Supplementary material for: Synthesis of a Coumarin-Based Analogue of Schweinfurthin F
Source: J Org Chem. 2021 Oct 29;86(23):16824–33. doi: 10.1021/acs.joc.1c02046 (PMC8650015; doi:10.1021/acs.joc.1c02046)
Supplement: Supplementary file 1 — jo1c02046_si_001.pdf [file jo1c02046_si_001.pdf]

Synthesis of a Coumarin-based Analogue of Schweinfurthin F

Chloe M. Schroeder,[a] Patrick N. Dey,[a] John A. Beutler[b] and David F. Wiemer[a]*

[a]*Department of Chemistry University of Iowa, Iowa City, Iowa 52242-1294*

[b]*Molecular Targets Laboratory, Center for Cancer Research, NCI-Frederick, Frederick, MD 21702*

david-wiemer@uiowa.edu

Table of Contents

400 MHz ^{1}H NMR spectrum of **23** in $D_3CC(O)CD_3$ S3
400 MHz ^{1}H NMR spectrum of **24** in $CDCl_3$ S4
100 MHz $^{13}C\{^1H\}$ NMR spectrum of **24** in $CDCl_3$ S5
400 MHz ^{1}H NMR spectrum of **25** in $CDCl_3$ S6
400 MHz ^{1}H NMR spectrum of **21** in $(CD_3)_2CO$ S7
100 MHz $^{13}C\{^1H\}$ NMR spectrum of **21** in $(CD_3)_2CO$ S8
100 MHz DEPT-135 NMR spectrum of **21** in $(CD_3)_2CO$ S9
400 MHz ^{1}H NMR spectrum of **19** in $CDCl_3$ S10
100 MHz $^{13}C\{^1H\}$ NMR spectrum of **19** in $CDCl_3$ S11
100 MHz DEPT-135 NMR spectrum of **19** in $CDCl_3$ S12
300 MHz ^{1}H NMR spectrum of **26** in $CDCl_3$ S13
75 MHz $^{13}C\{^1H\}$ NMR spectrum of **26** in $CDCl_3$ S14
75 MHz DEPT-135 of **26** in $CDCl_3$ S15
300 MHz ^{1}H NMR spectrum of **28** in $CDCl_3$ S16
75 MHz $^{13}C\{^1H\}$ NMR spectrum of **28** in $CDCl_3$ S17
75 MHz DEPT-135 of **28** in $CDCl_3$ S18
400 MHz ^{1}H NMR spectrum of **29** in $CDCl_3$ S19
100 MHz $^{13}C\{^1H\}$ NMR spectrum of **29** in $CDCl_3$ S20
100 MHz DEPT-135 of **29** in $CDCl_3$ S21
400 MHz ^{1}H NMR spectrum of **30** in $CDCl_3$ S22
100 MHz $^{13}C\{^1H\}$ NMR spectrum of **30** in $CDCl_3$ S23
100 MHz DEPT-135 of **30** in $CDCl_3$ S24

400 MHz ^{1}H NMR spectrum of **32** in $CDCl_3$ S25
100 MHz $^{13}C\{^{1}H\}$ NMR spectrum of **32** in $CDCl_3$ S26
100 MHz DEPT-135 NMR spectrum of **32** in $CDCl_3$ S27
400 MHz ^{1}H NMR spectrum of **17** in $CDCl_3$ S28
300 MHz ^{1}H NMR spectrum of **15** in $CDCl_3$ S29
121 MHz ^{31}P NMR spectrum of **15** in $CDCl_3$.......... S30
400 MHz ^{1}H NMR spectrum of **34** in $CDCl_3$ S31
75 MHz $^{13}C\{^{1}H\}$ NMR spectrum of **34** in $CDCl_3$ S32
400 MHz ^{1}H NMR spectrum of **35** in $CDCl_3$ S33
400 MHz ^{1}H NMR spectrum of **36** in $CDCl_3$ S34
100 MHz $^{13}C\{^{1}H\}$ NMR spectrum of **36** in $CDCl_3$ S35
100 MHz DEPT-135 of **36** in $CDCl_3$.......... S36
300 MHz ^{1}H NMR spectrum of **37** in $CDCl_3$ S37
100 MHz $^{13}C\{^{1}H\}$ NMR spectrum of **37** in $CDCl_3$ S38
100 MHz DEPT-135 ^{13}C of **37** in $CDCl_3$.......... S39
400 MHz ^{1}H NMR spectrum of **38** in $CDCl_3$ S40
100 MHz $^{13}C\{^{1}H\}$ NMR spectrum of **38** in $CDCl_3$ S41
100 MHz DEPT-135 NMR spectrum of **38** in $CDCl_3$.......... S42
400 MHz ^{1}H NMR spectrum of **39** in $CDCl_3$ S43
100 MHz 13C$\{^{1}H\}$ NMR spectrum of **39** in $CDCl_3$.......... S44
400 MHz ^{1}H NMR spectrum of **40** in $CDCl_3$ S45
100 MHz $^{13}C\{^{1}H\}$ NMR spectrum of **40** in $CDCl_3$ S46
100 MHz DEPT-135 NMR spectrum of **40** in $CDCl_3$ S47
Dose-response data for compound **40** in the 60 cell line screen S48
Bioassay data for compound **40** in the 60 cell line screen S49
400 MHz ^{1}H NMR spectrum of **41** in CD_3CN S50
600 MHz 2D HMBC spectrum of **41** in CD_3CN S51
Phase sensitive HSQC spectrum of compound **41** in CD_3CN S52
Absorption spectrum of compound **41** in EtOH S53
Emission spectrum of compound **41** in EtOH upon excitation at 420 nm.......... S54
Dose-response data for compound **41** in the 60 cell line screen S55
Bioassay data for compound **41** in the 60 cell line screen S56
400 MHz ^{1}H NMR spectrum of **42** in $CDCl_3$ S57
400 MHz ^{1}H NMR spectrum of **43** in $CDCl_3$ S58
100 MHz $^{13}C\{^{1}H\}$ NMR spectrum of **43** in $CDCl_3$ S59
100 MHz DEPT-135 of **43** in $CDCl_3$.......... S60
HPLC trace of compound **41** S61

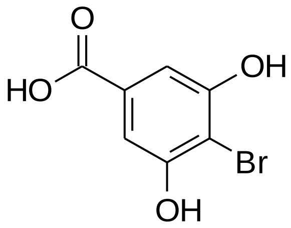

23

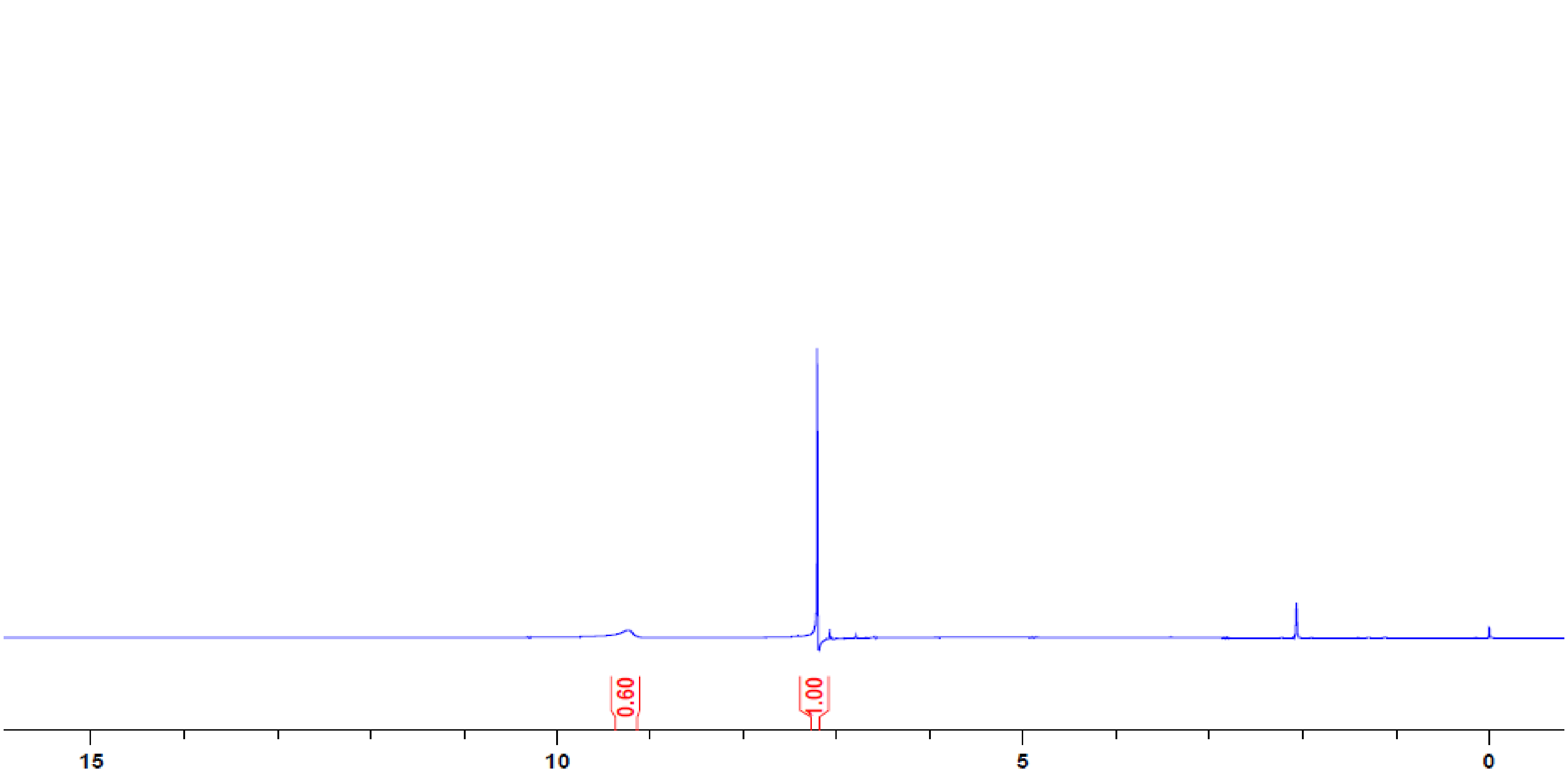

400 MHz ^{1}H NMR spectrum of **23** in $D_3CC(O)CD_3$

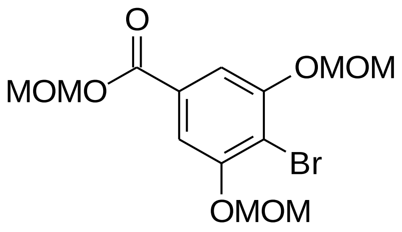

24

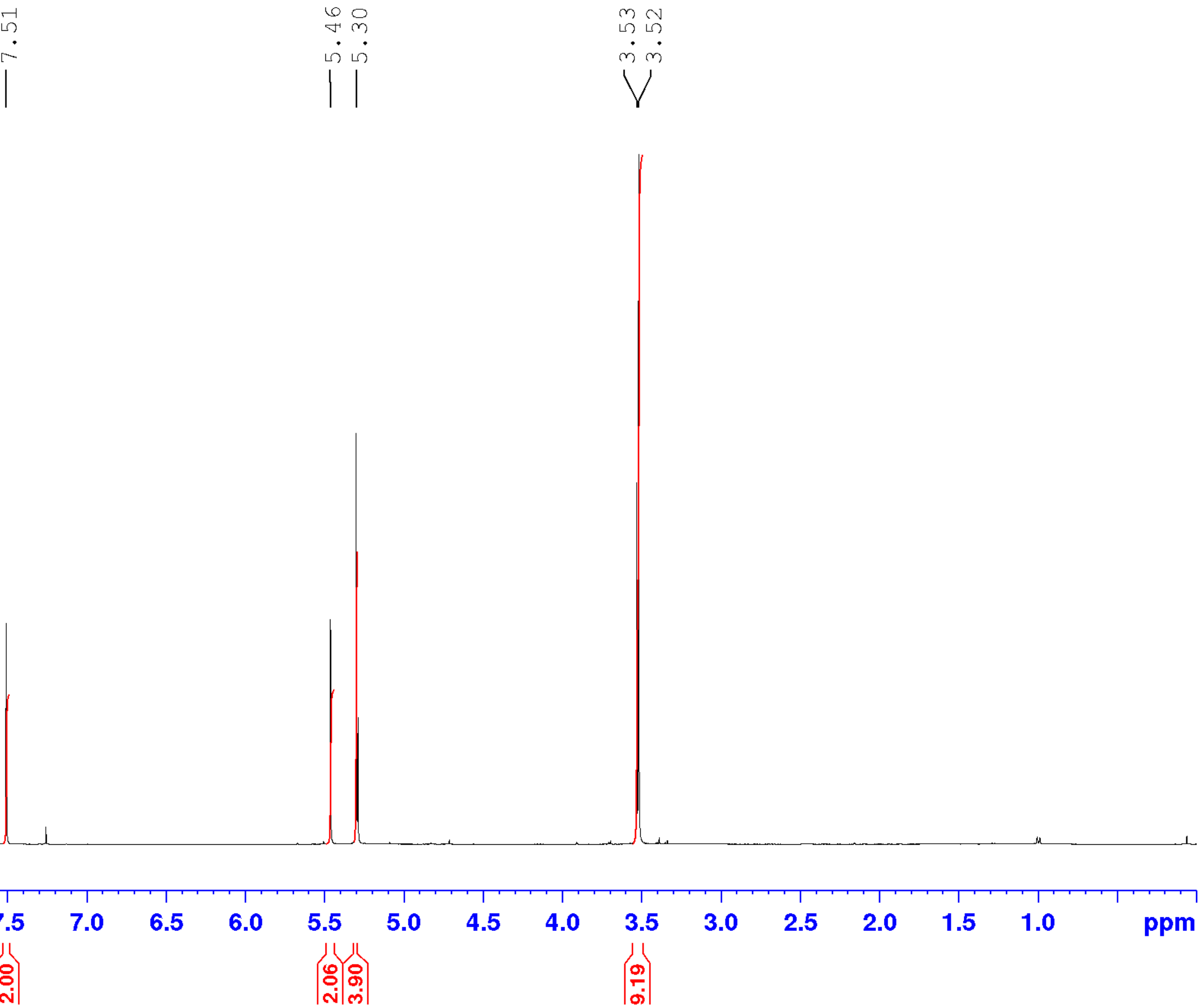

400 MHz ^{1}H NMR spectrum of **24** in $CDCl_3$

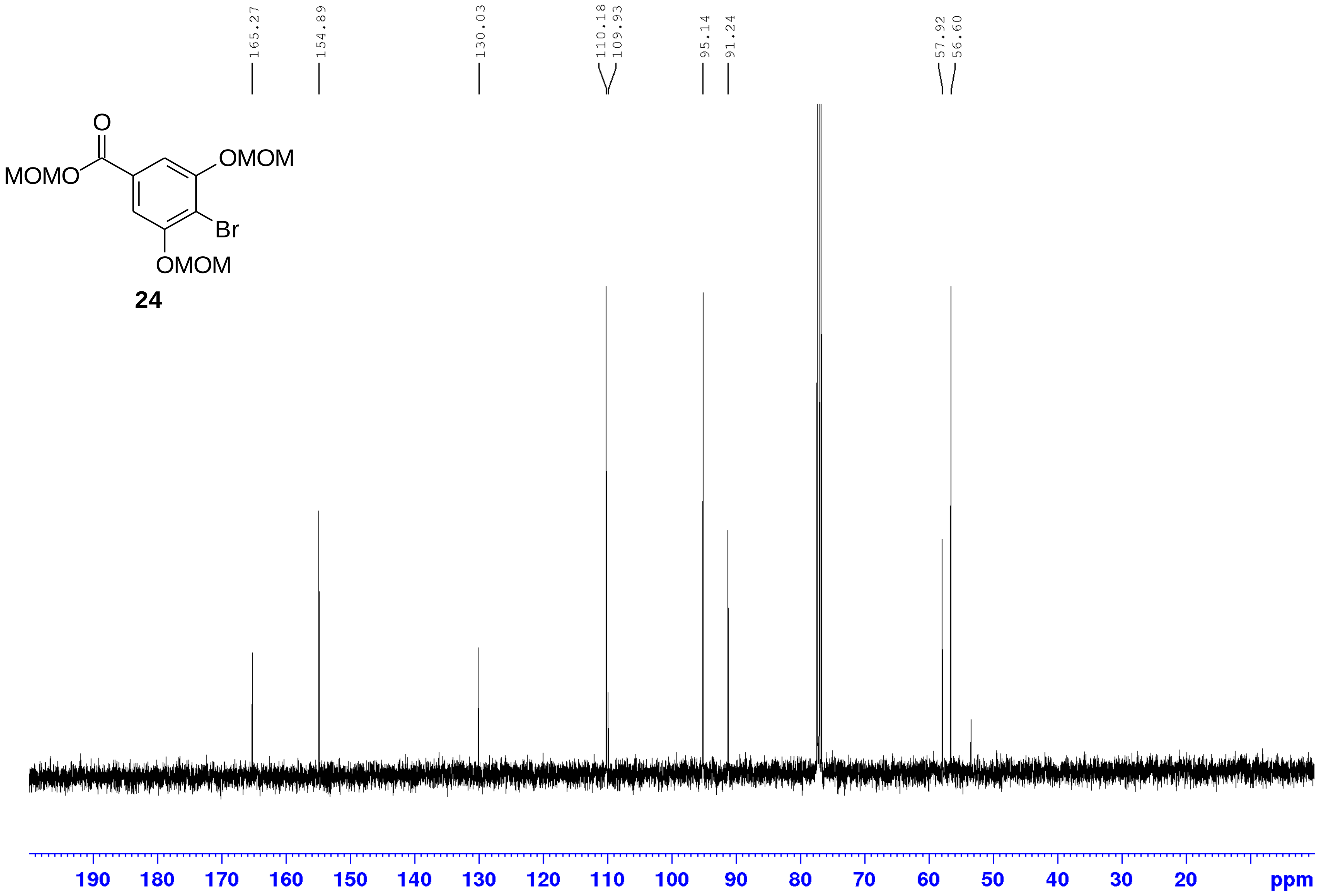

100 MHz $^{13}C\{^1H\}$ NMR spectrum of **24** in $CDCl_3$

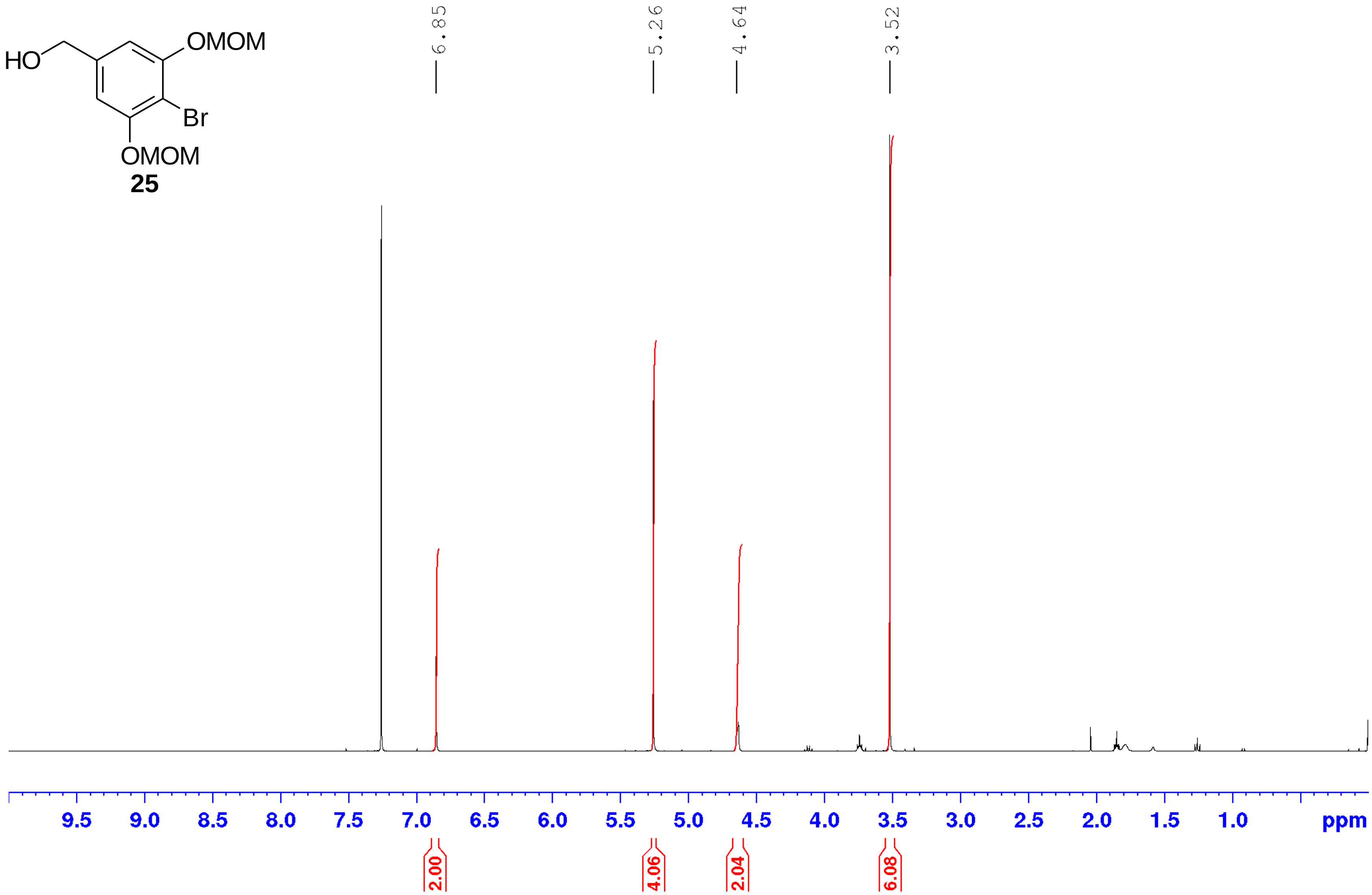

400 MHz ^{1}H NMR spectrum of **25** in $CDCl_3$

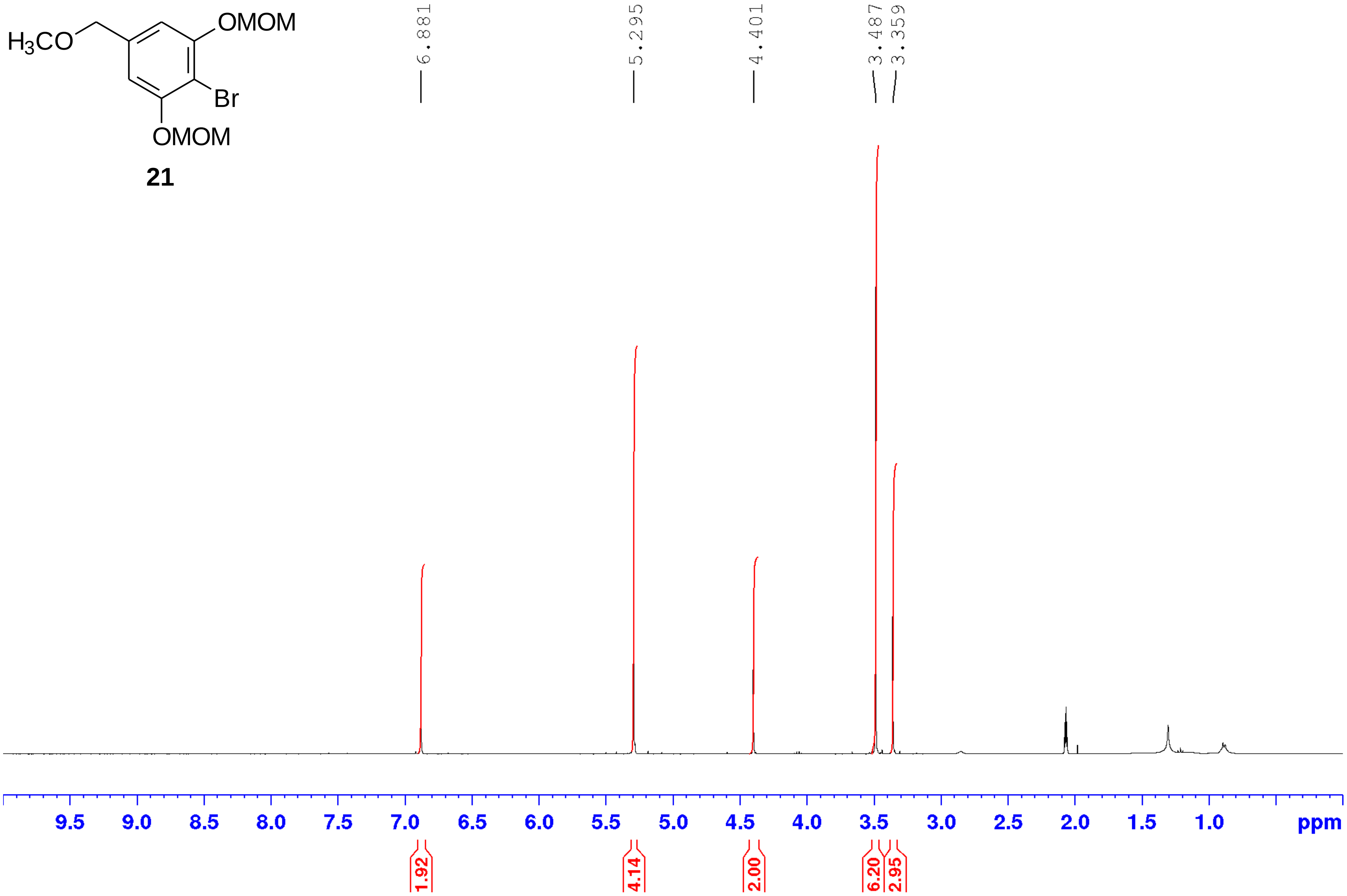

400 MHz ^{1}H NMR spectrum of **21** in $(CD_3)_2CO$

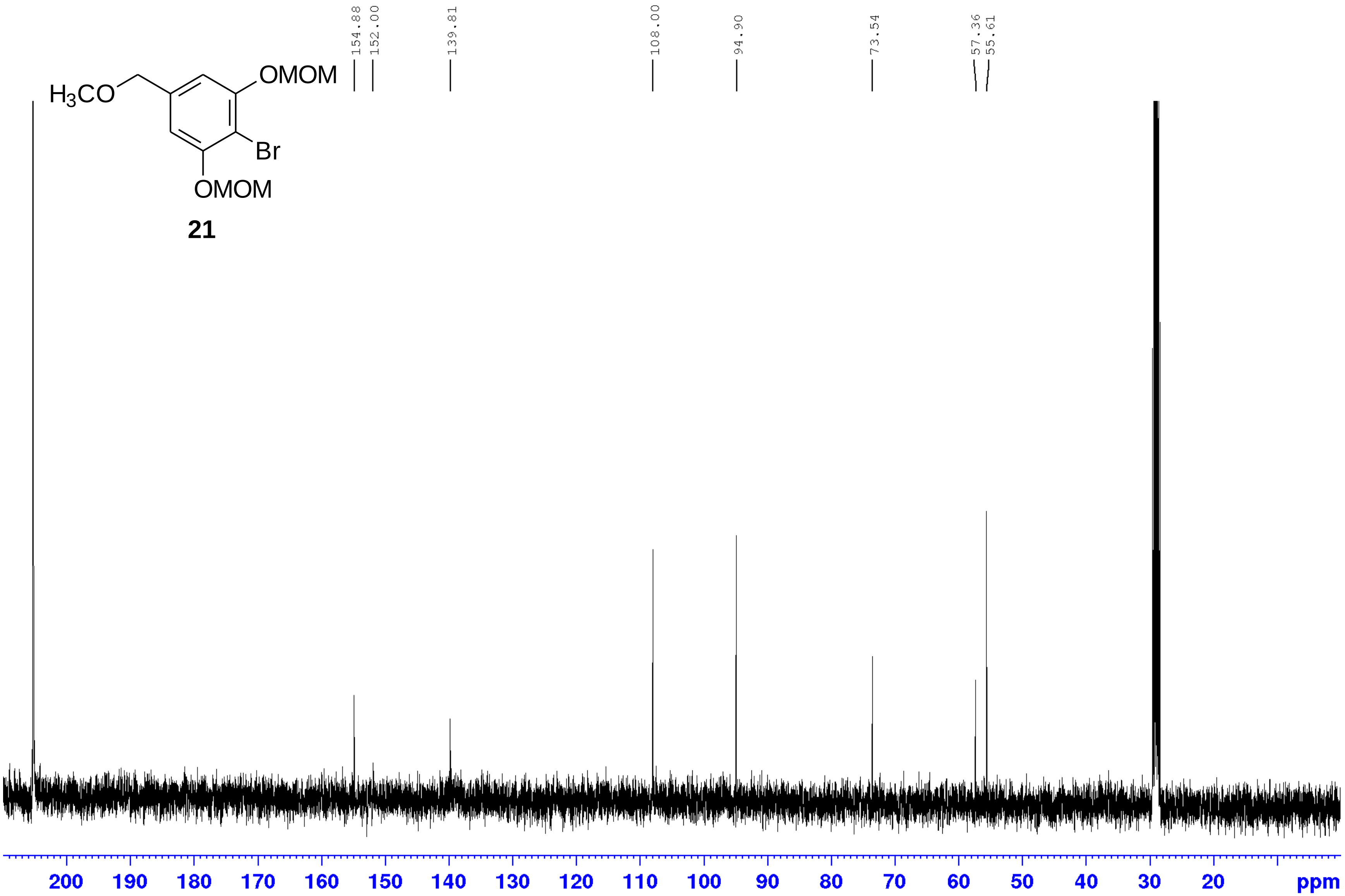

100 MHz $^{13}C\{^{1}H\}$ NMR spectrum of **21** in $(CD_3)_2CO$

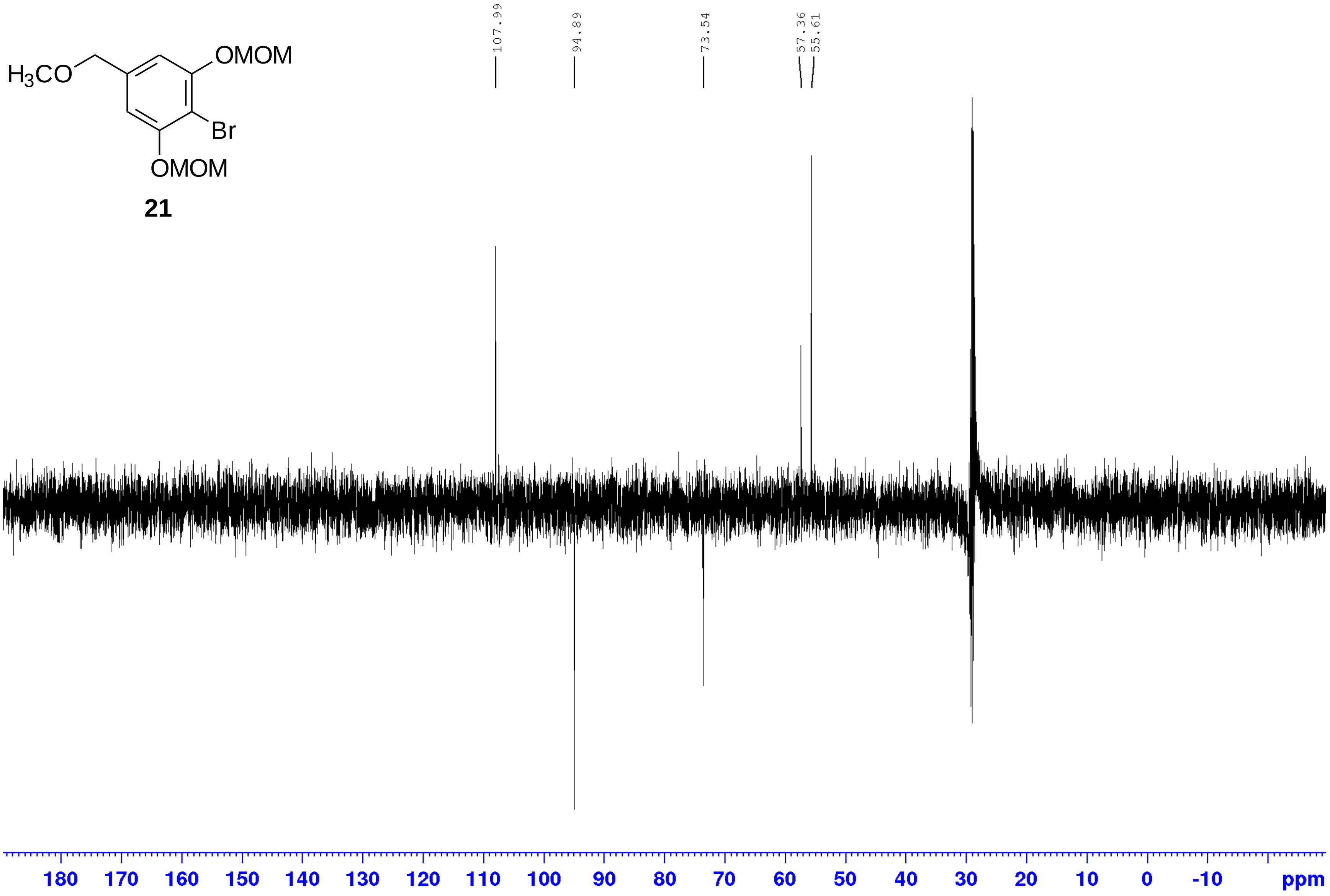

100 MHz DEPT-135 NMR spectrum of **21** in $(CD_3)_2CO$

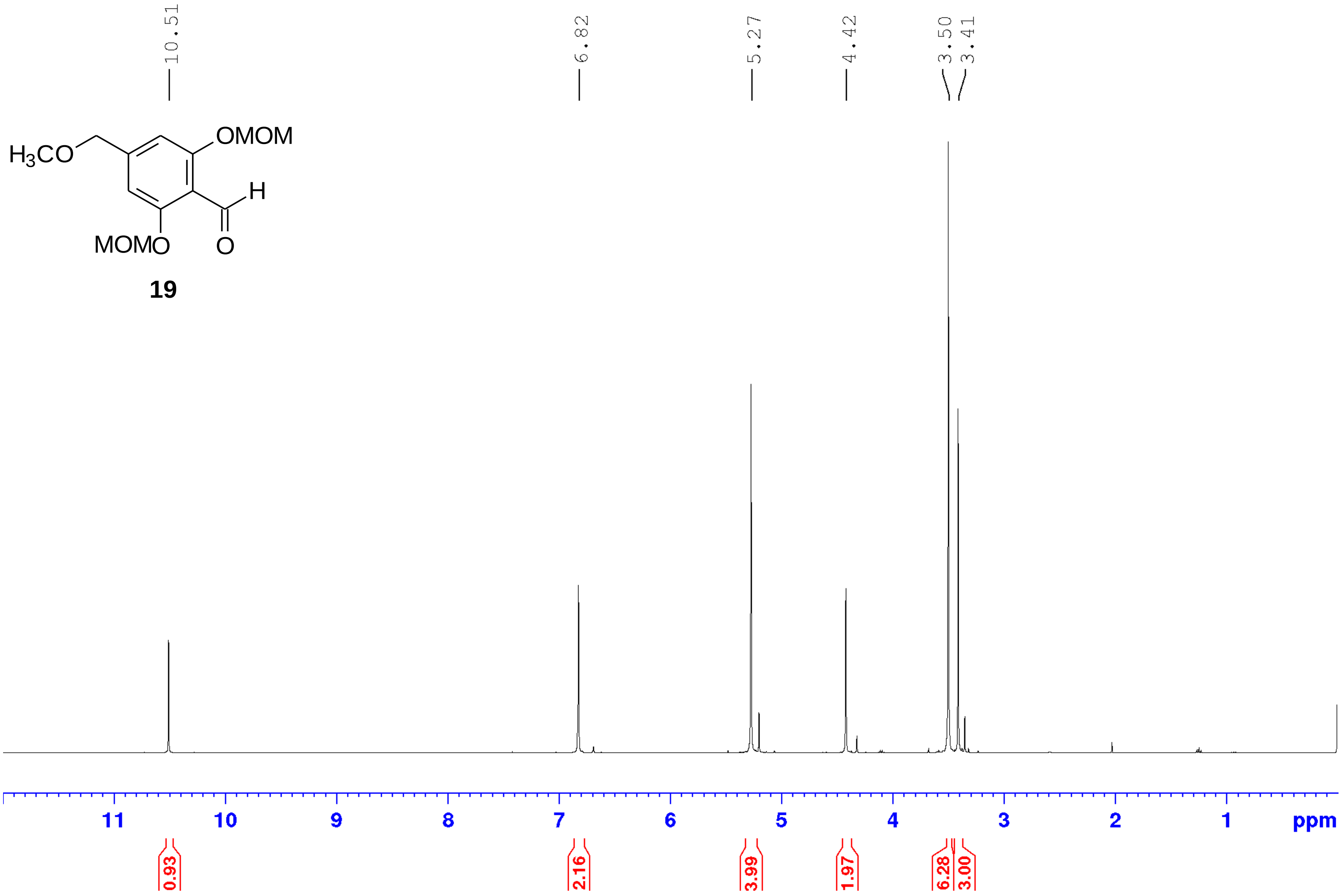

400 MHz ^{1}H NMR spectrum of **19** in $CDCl_3$

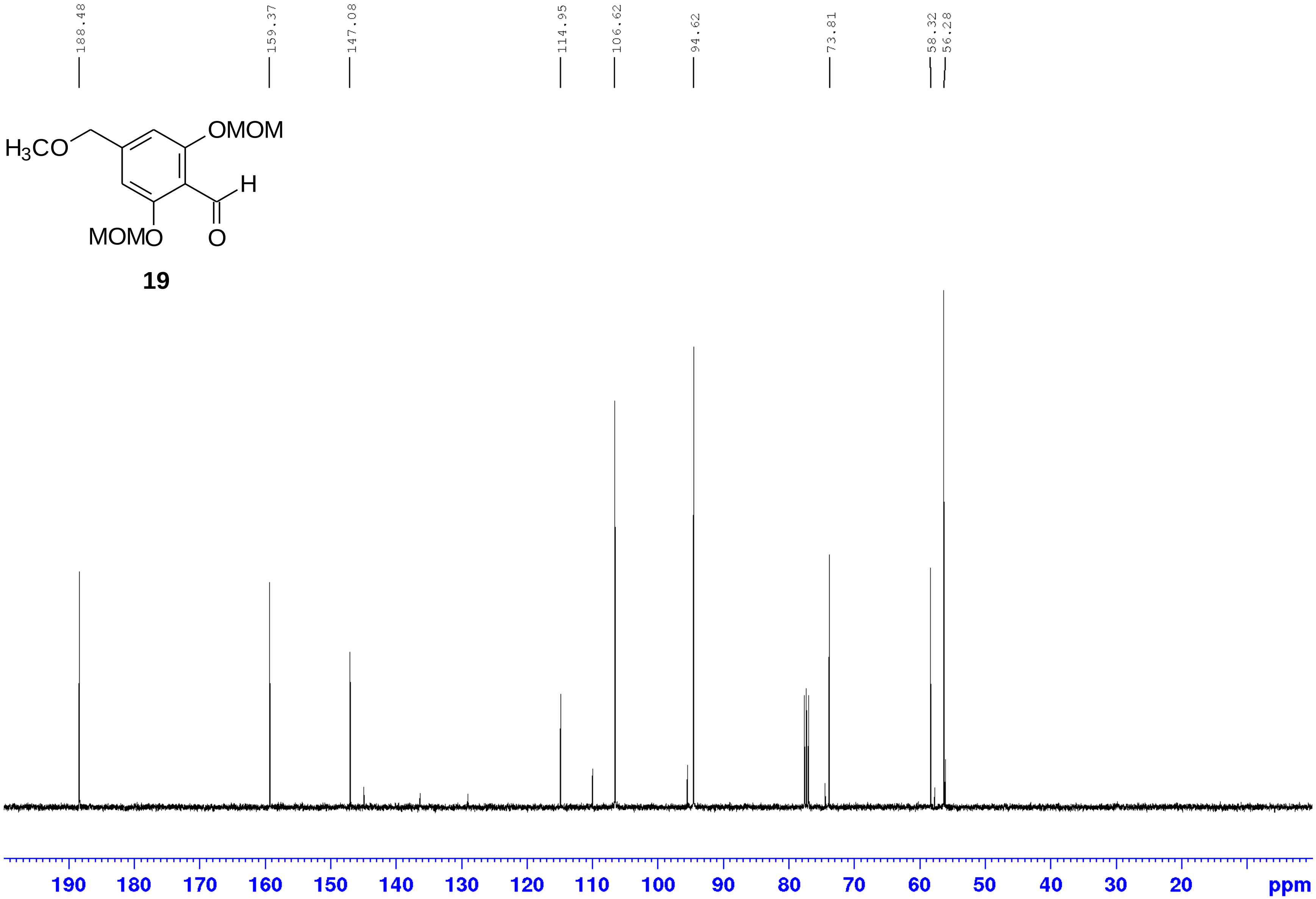

100 MHz $^{13}C\{^{1}H\}$ NMR spectrum of **19** in $CDCl_3$

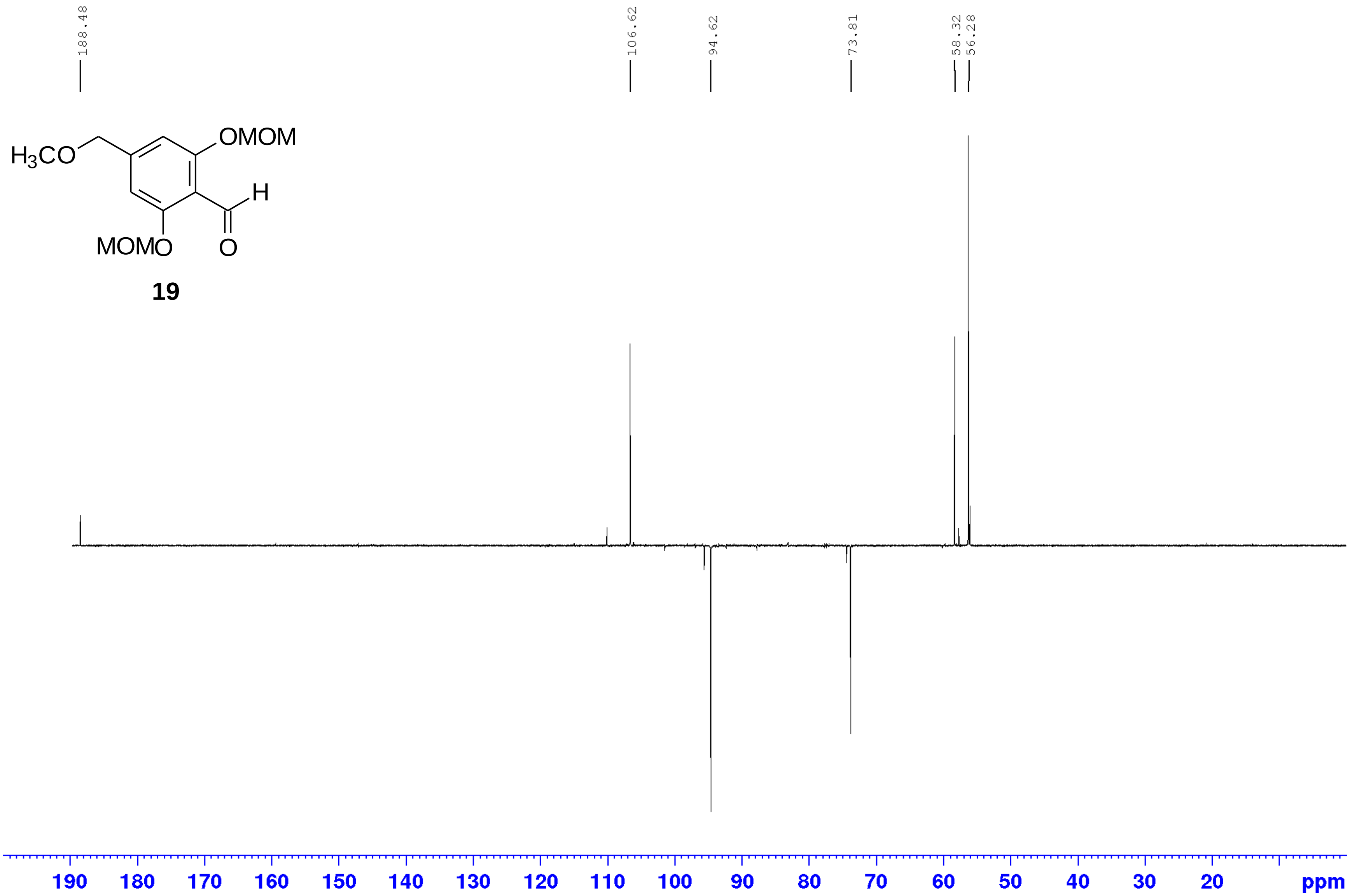

19

100 MHz DEPT-135 NMR spectrum of **19** in $CDCl_3$

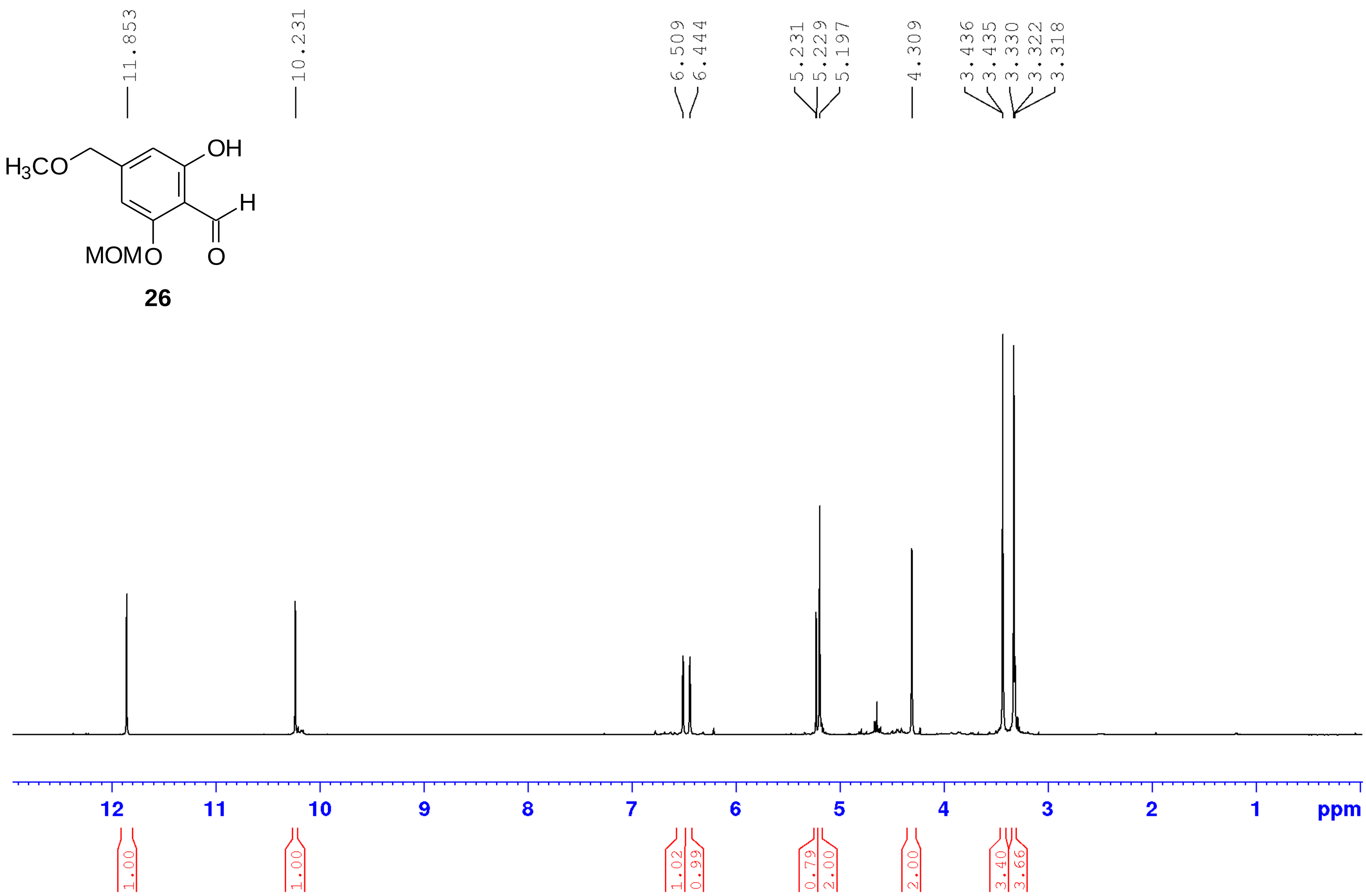

300 MHz ^{1}H NMR spectrum of **26** in $CDCl_3$

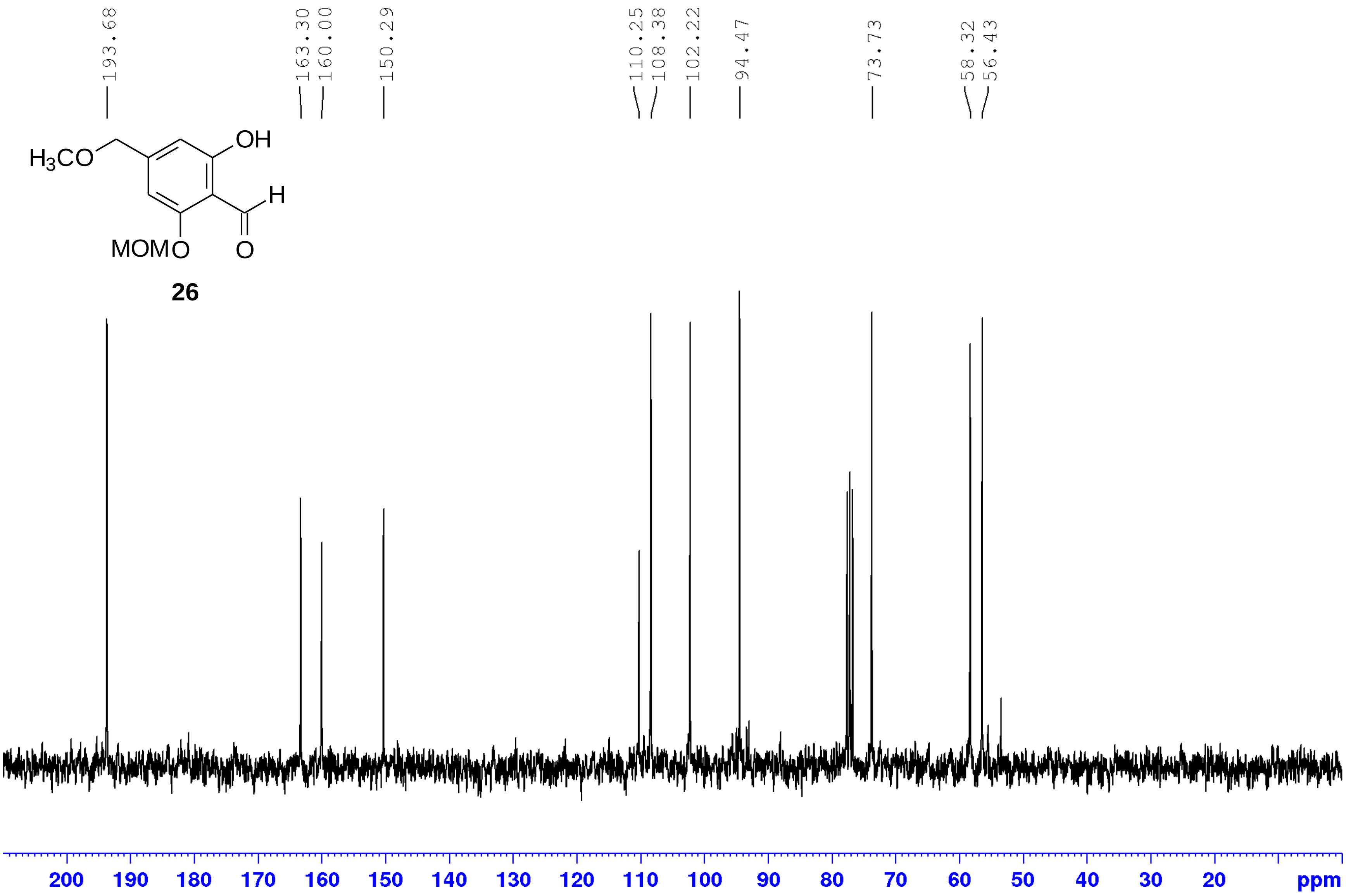

75 MHz $^{13}C\{^1H\}$ NMR spectrum of **26** in $CDCl_3$

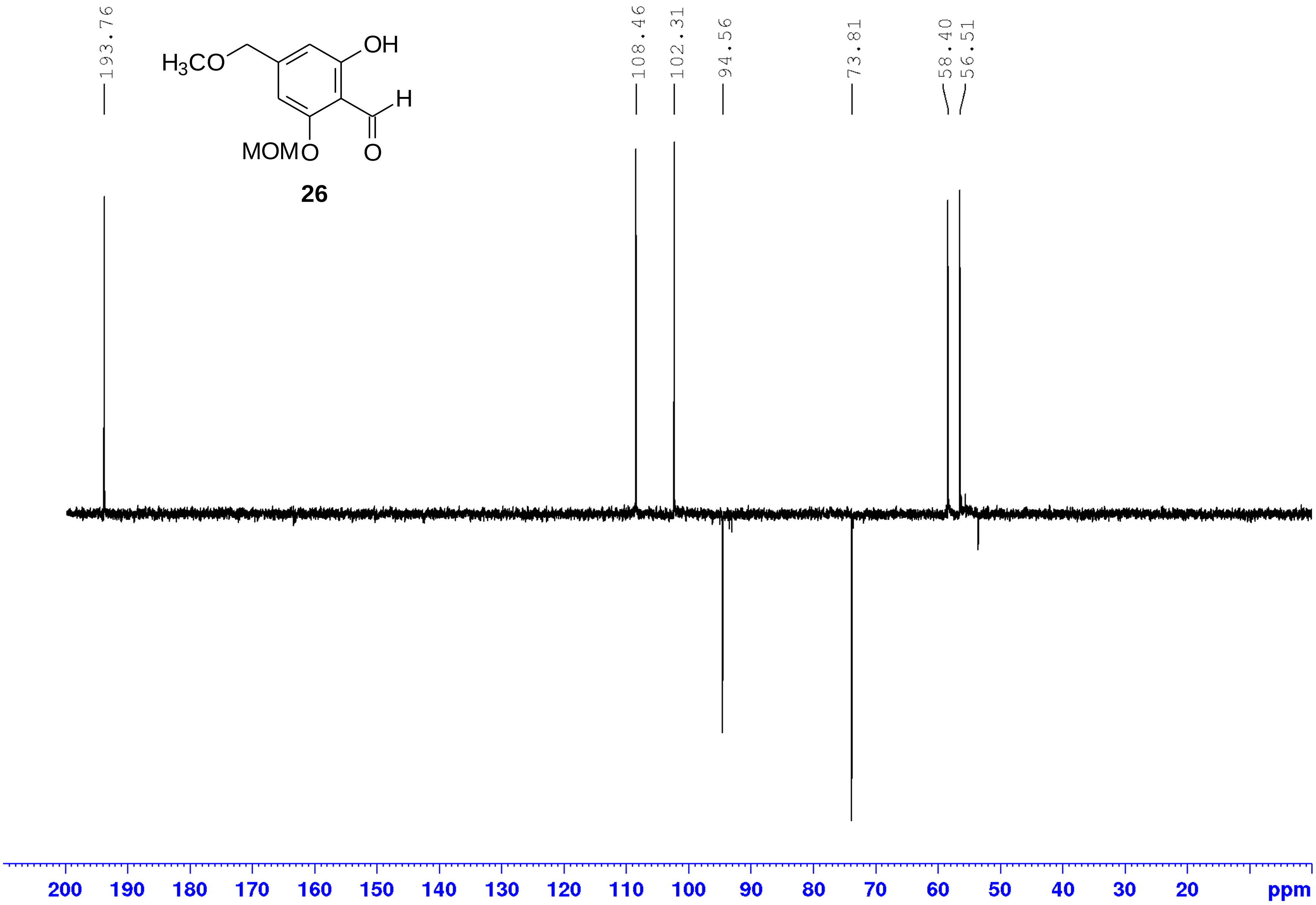

75 MHz DEPT-135 of **26** in $CDCl_3$

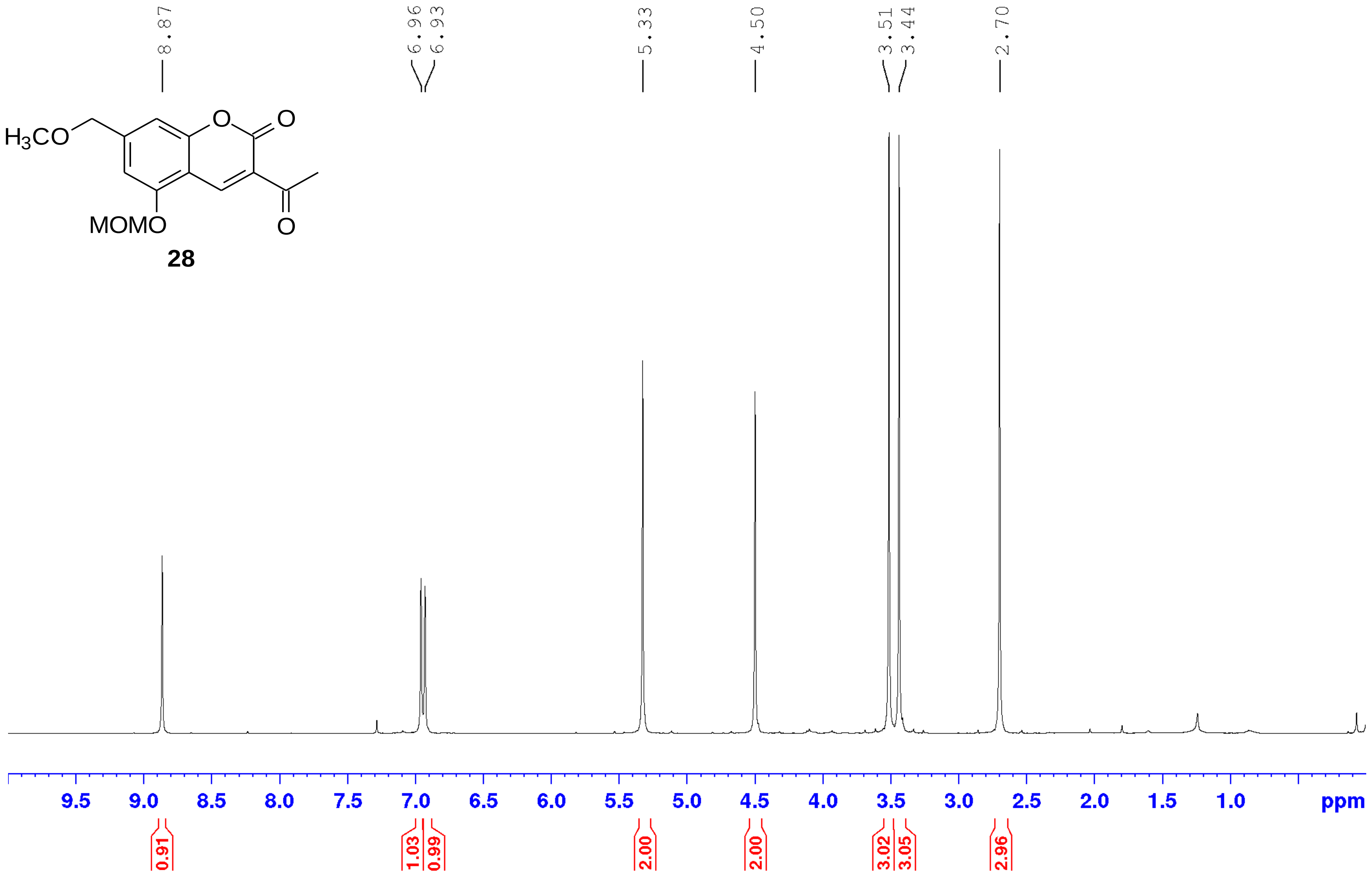

300 MHz ^{1}H NMR spectrum of **28** in $CDCl_3$

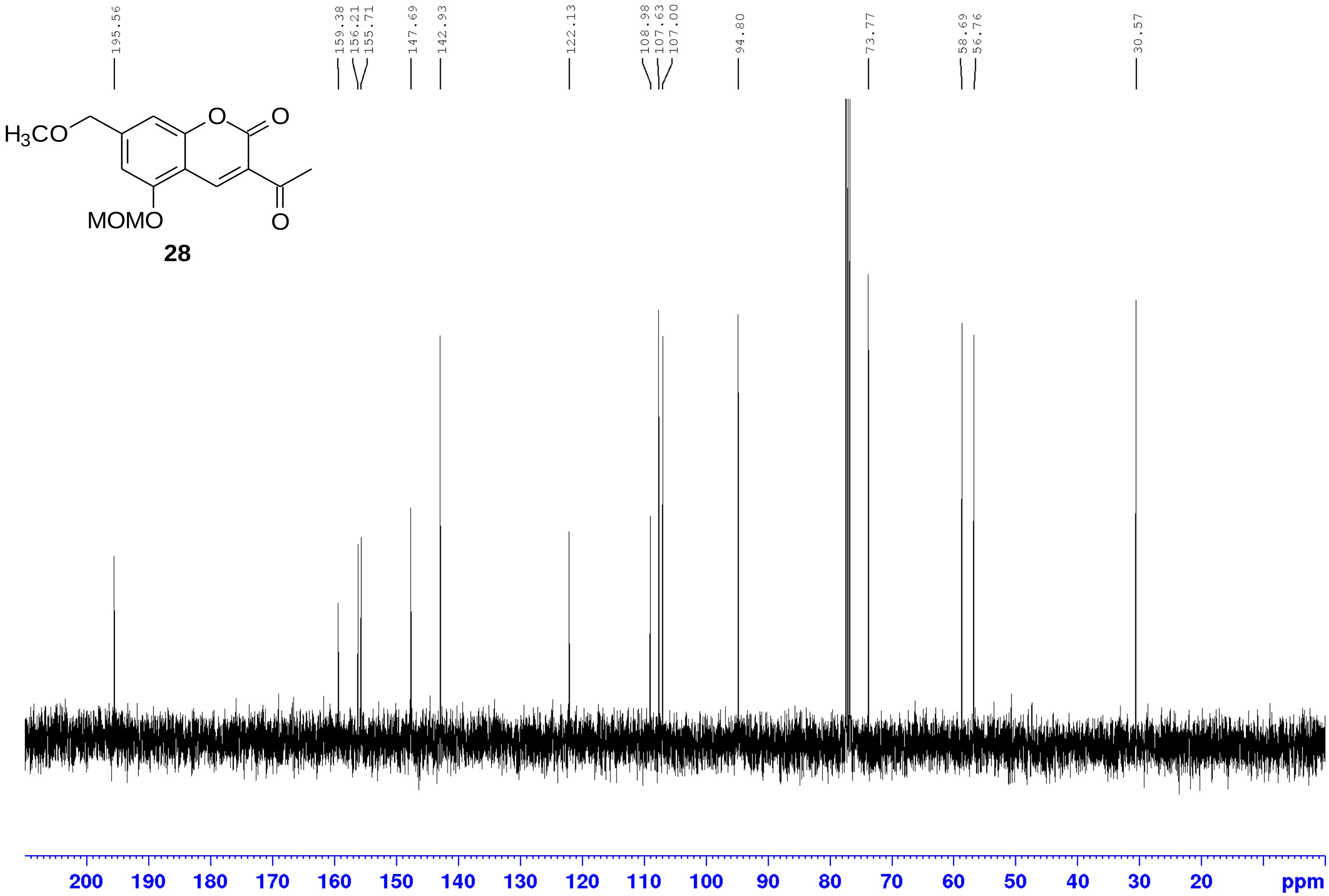

75 MHz $^{13}C\{^1H\}$ NMR spectrum of **28** in $CDCl_3$

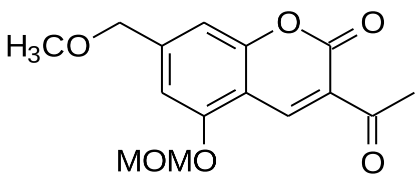

28

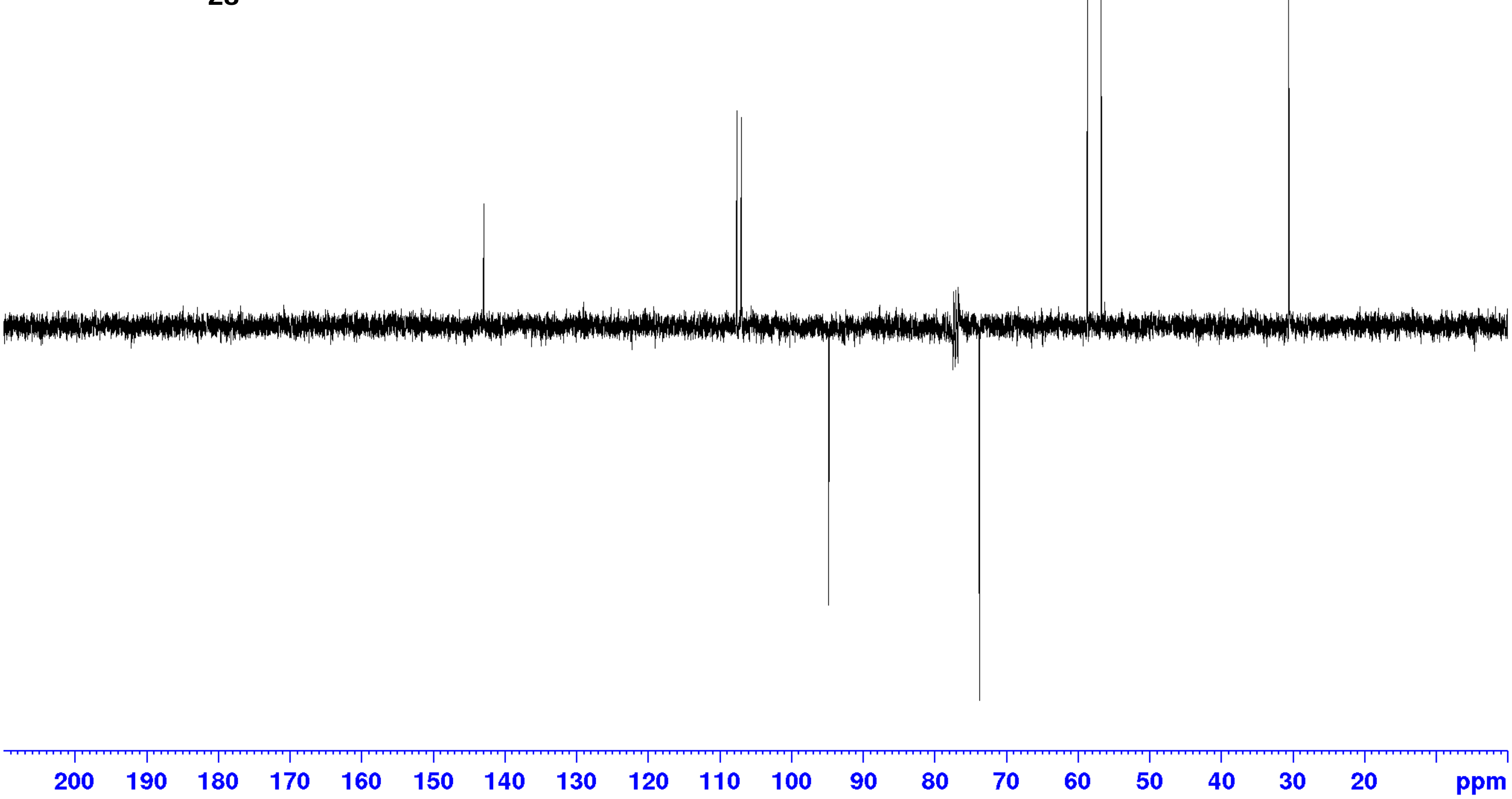

75 MHz DEPT-135 of **28** in $CDCl_3$

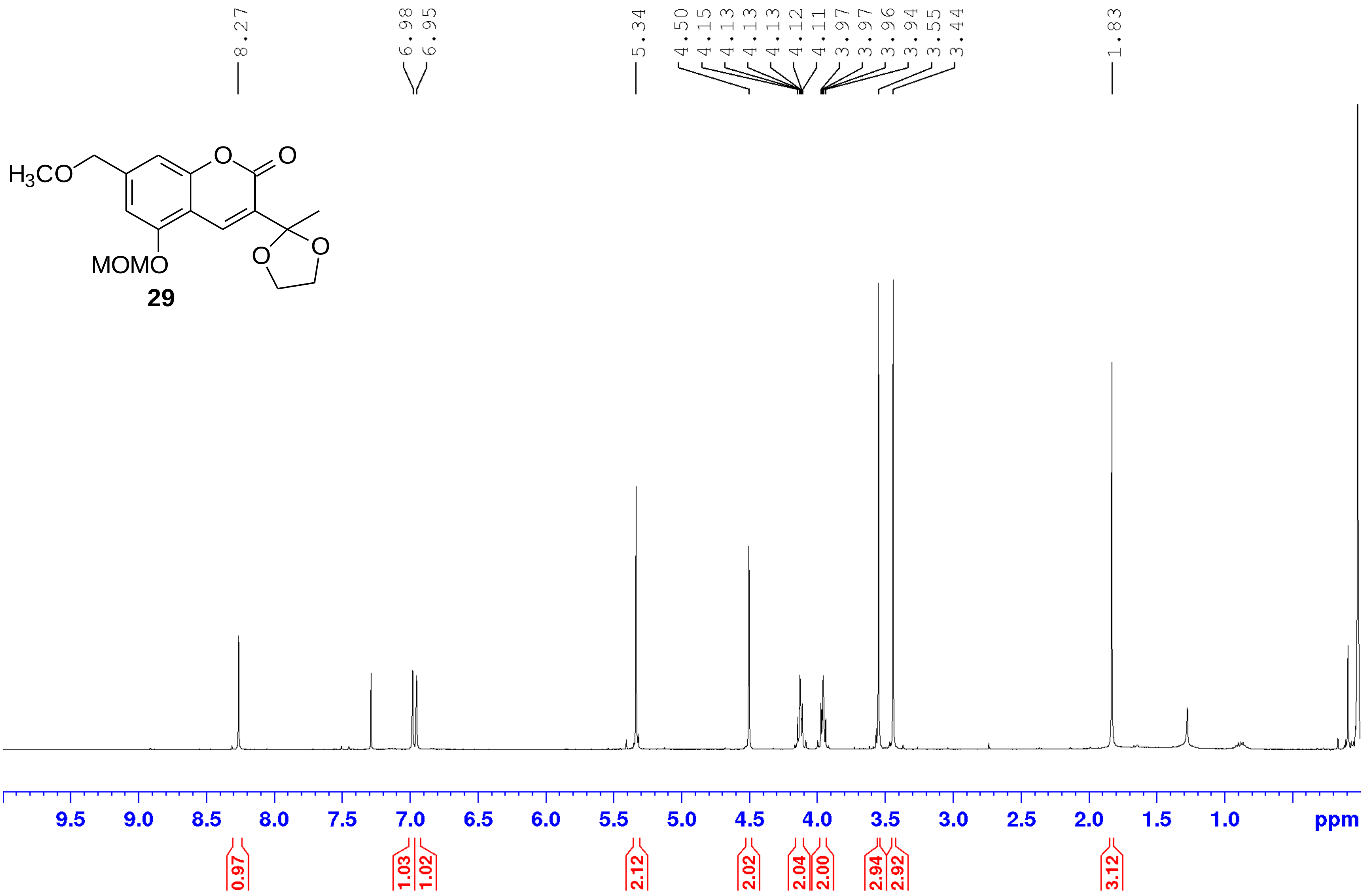

400 MHz ^{1}H NMR spectrum of **29** in $CDCl_3$

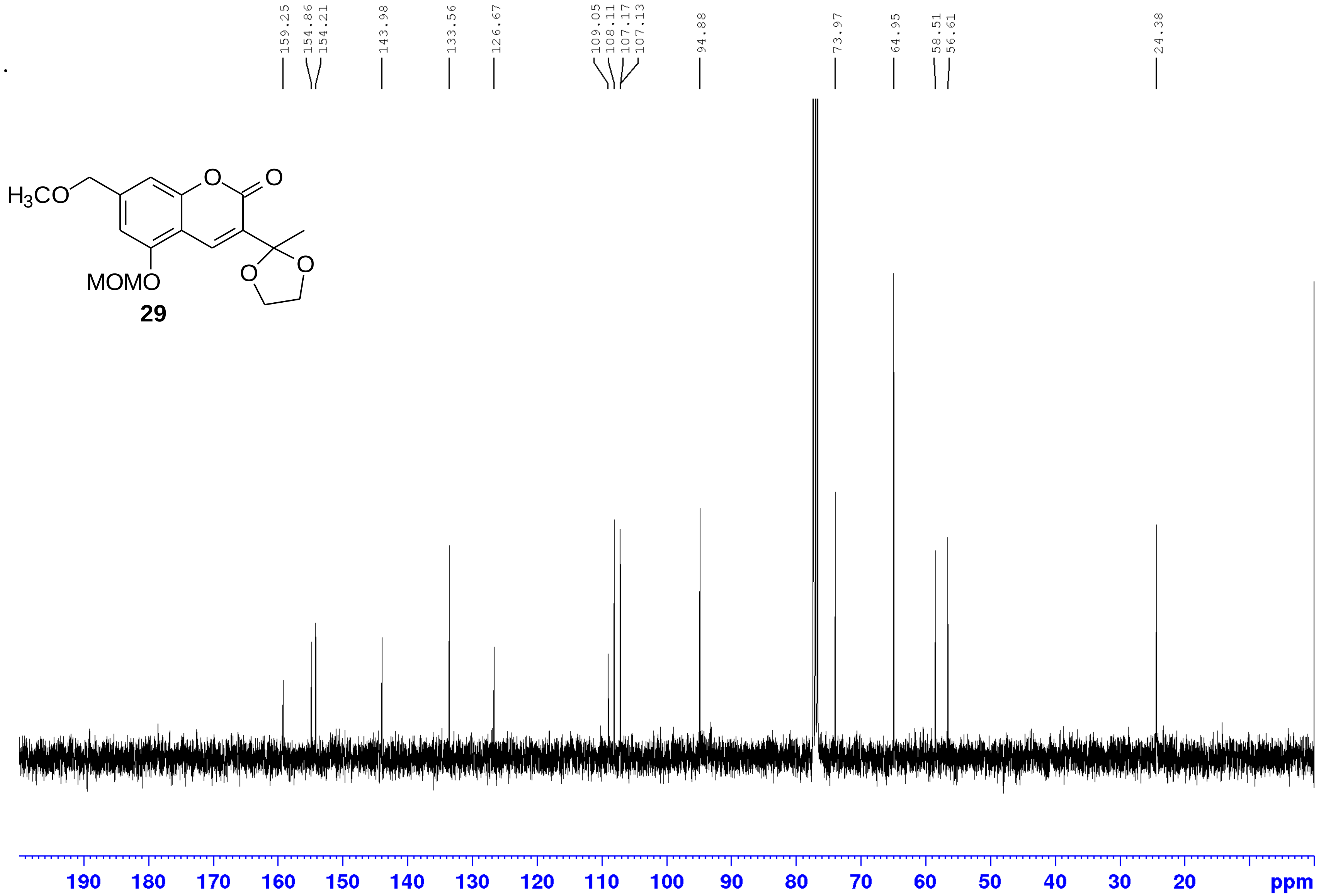

100 MHz $^{13}C\{^{1}H\}$ NMR spectrum of **29** in $CDCl_3$

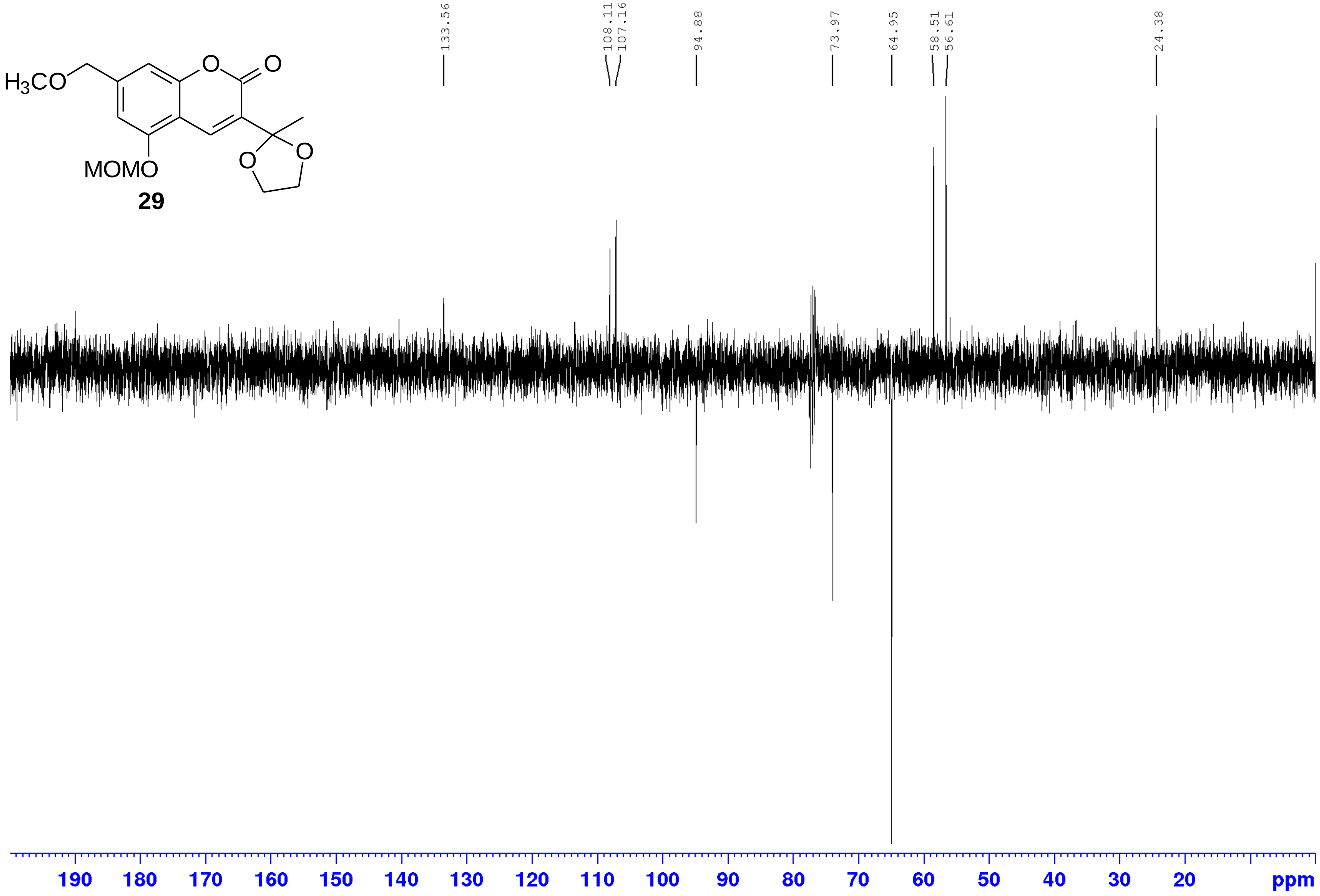

100 MHz DEPT-135 of **29** in $CDCl_3$

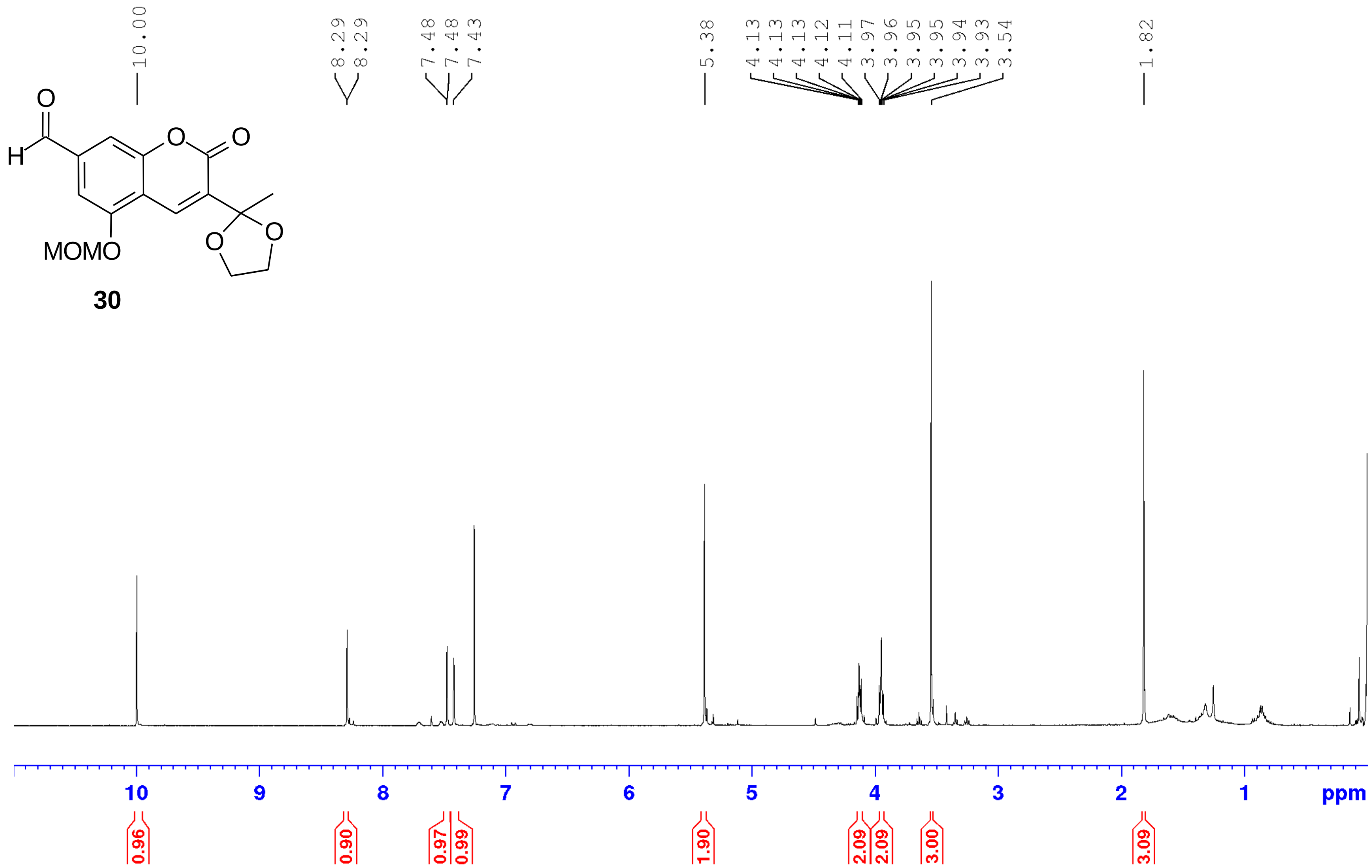

400 MHz ^{1}H NMR spectrum of **30** in $CDCl_3$

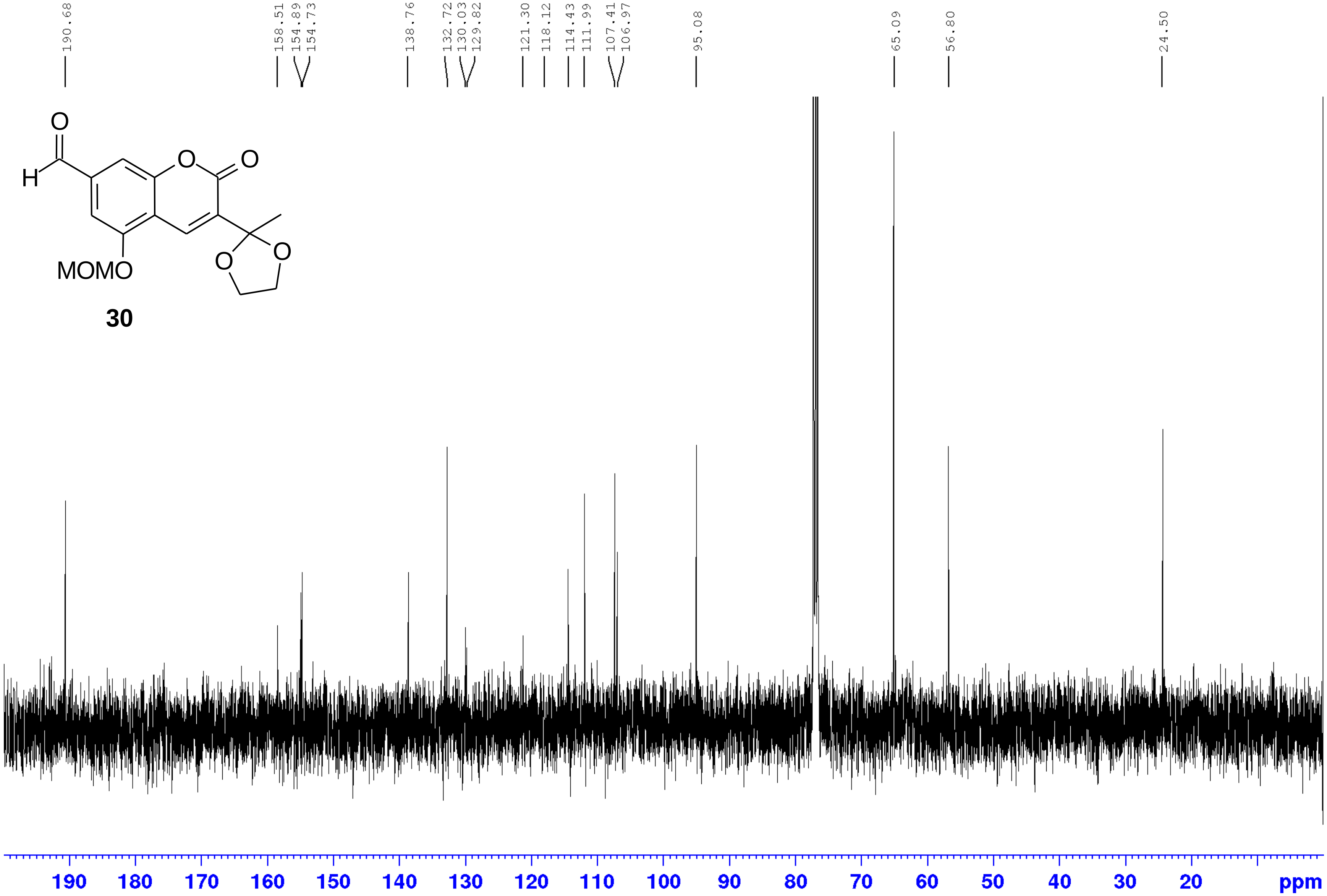

100 MHz $^{13}C\{^{1}H\}$ NMR spectrum of **30** in $CDCl_3$

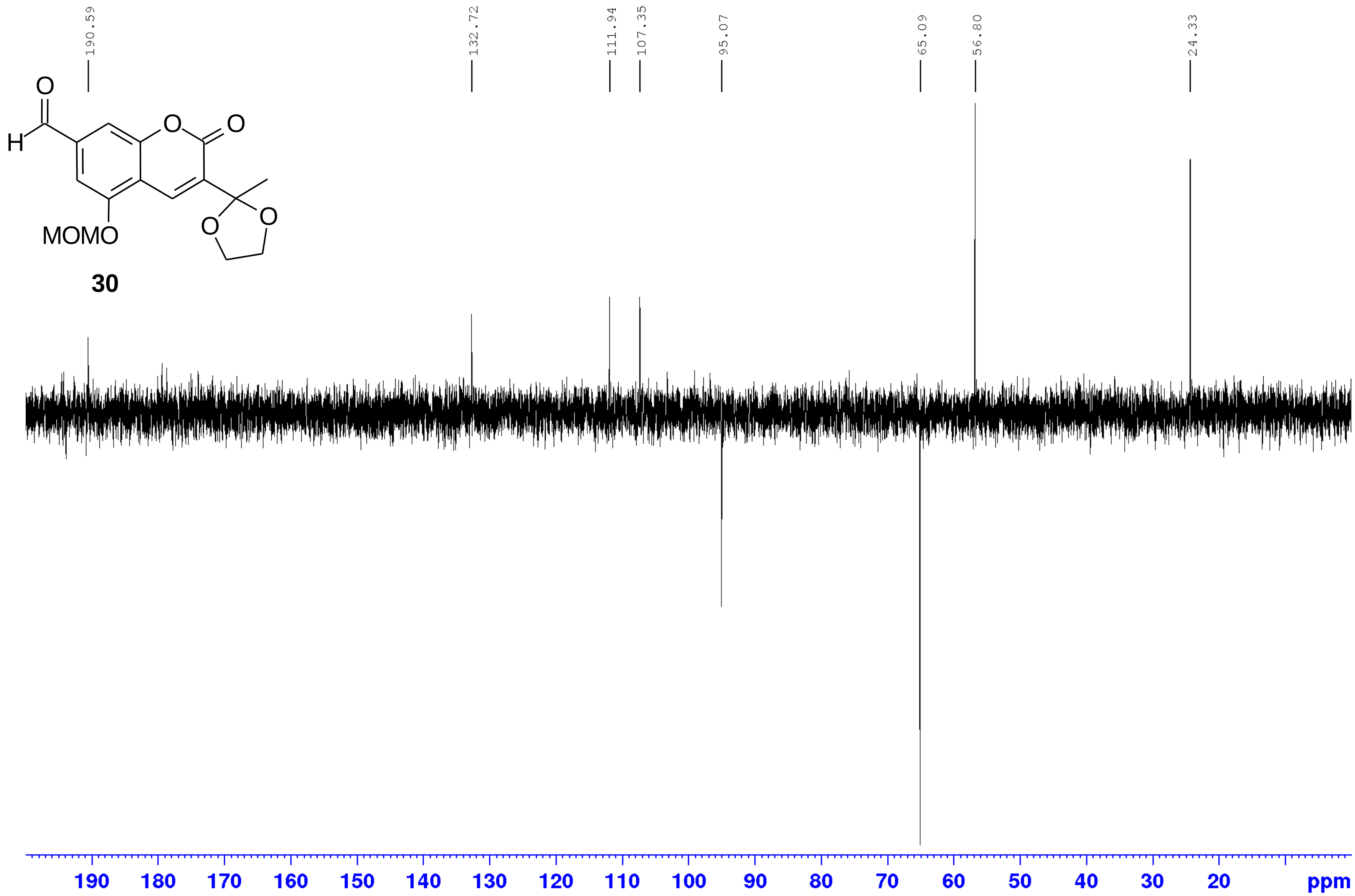

100 MHz DEPT-135 of **30** in $CDCl_3$

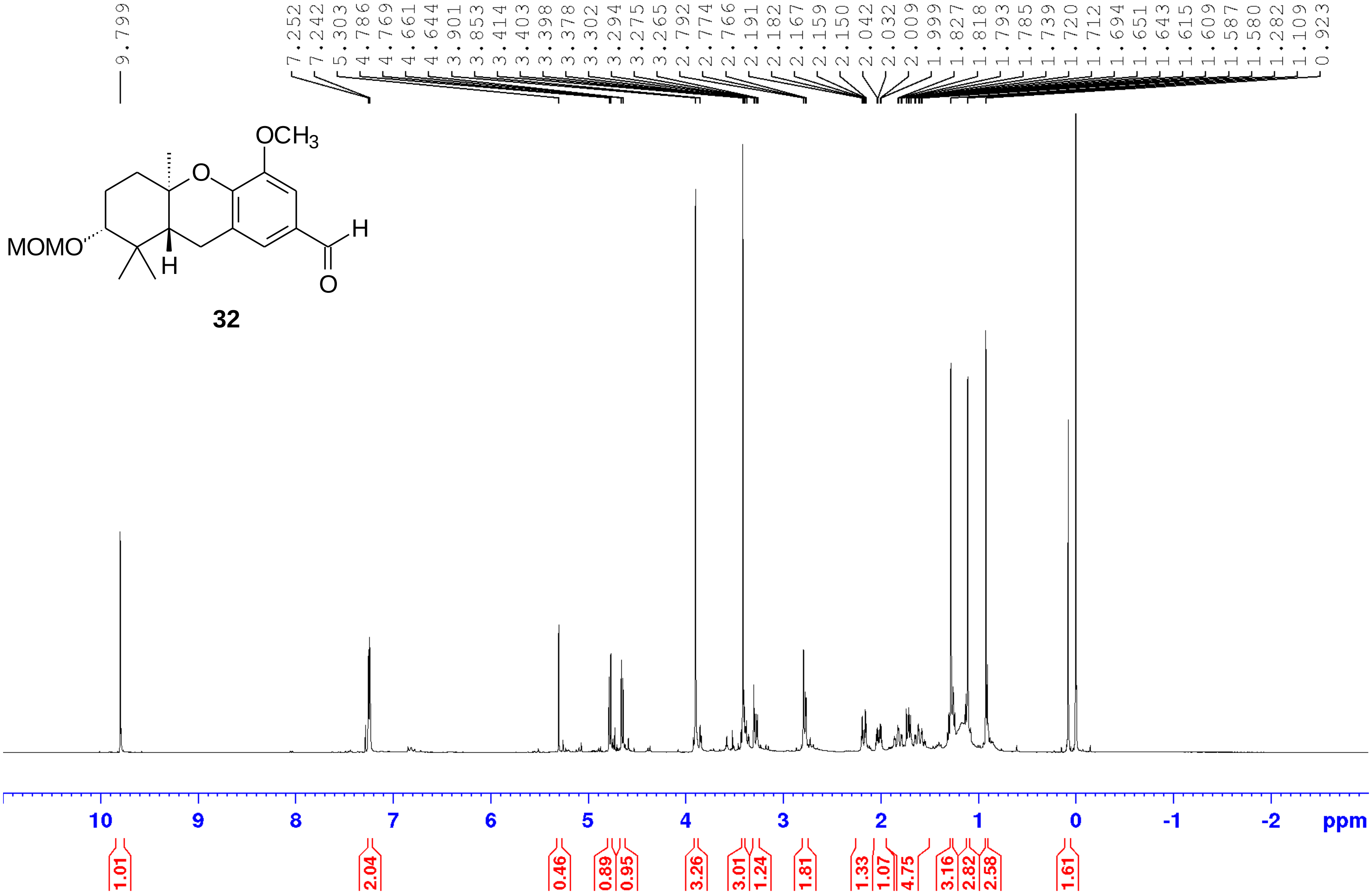

400 MHz ^1H NMR spectrum of **32** in $CDCl_3$

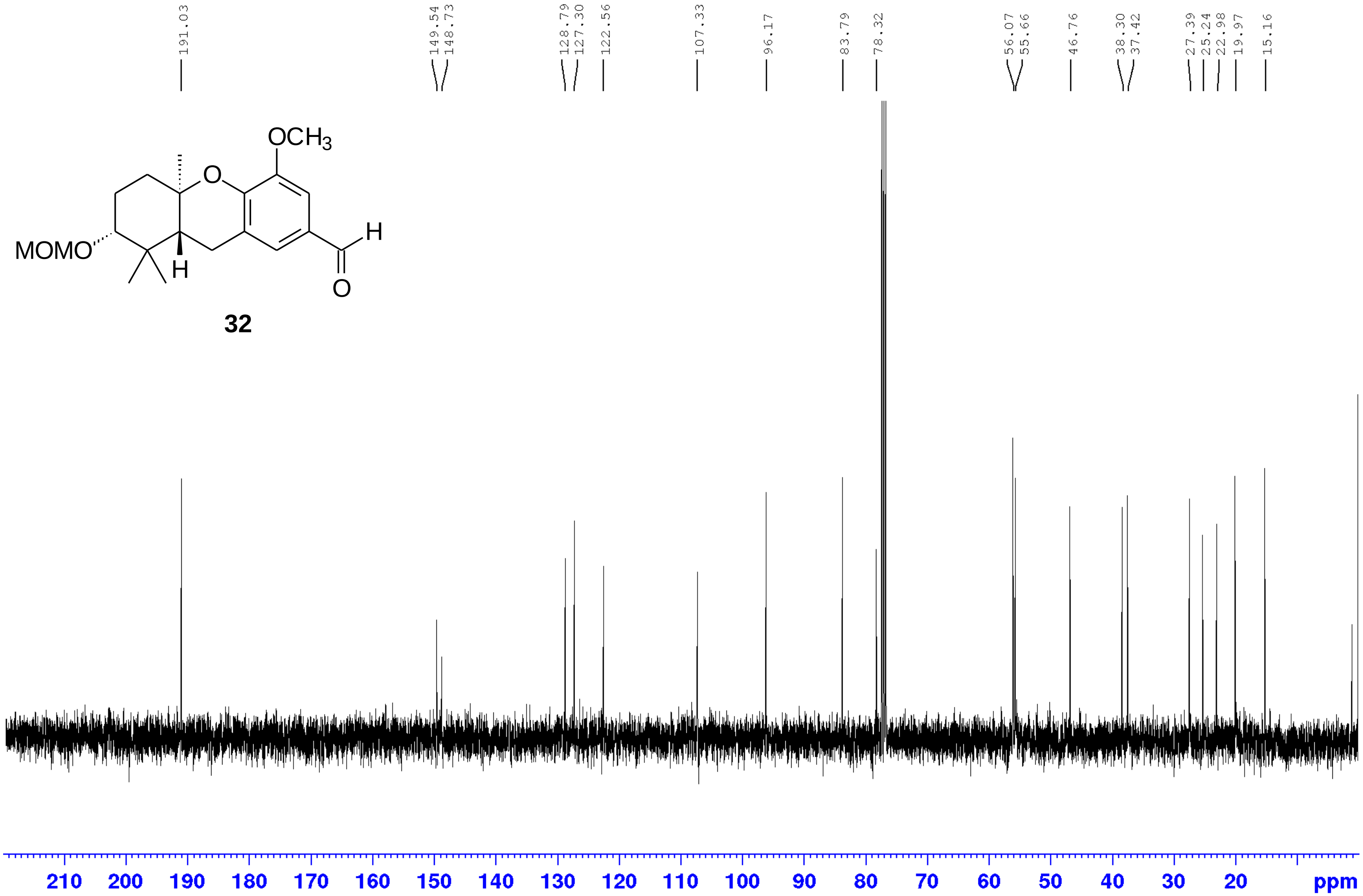

32

100 MHz $^{13}C\{^{1}H\}$ NMR spectrum of **32** in $CDCl_3$

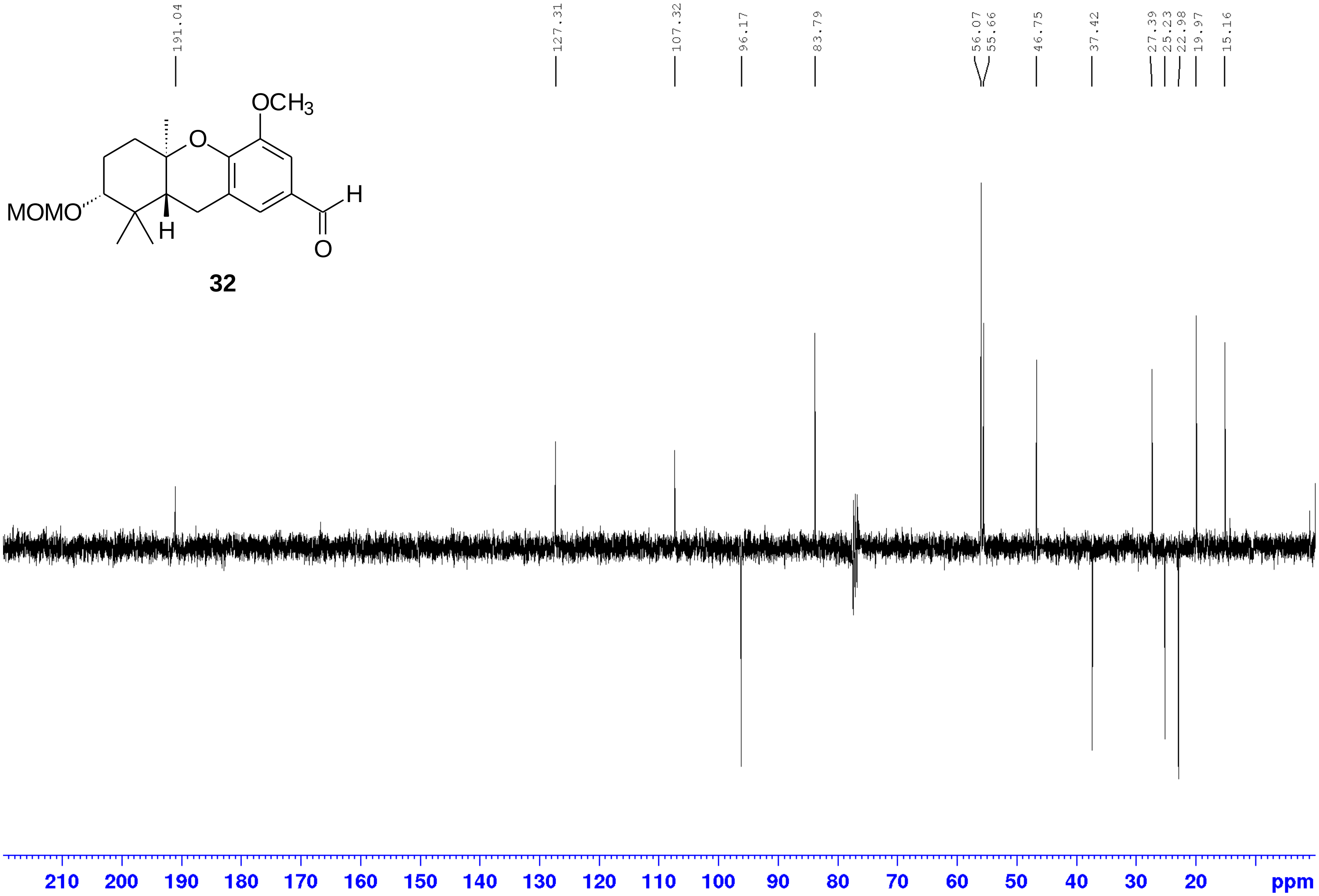

100 MHz DEPT-135 NMR spectrum of **32** in $CDCl_3$

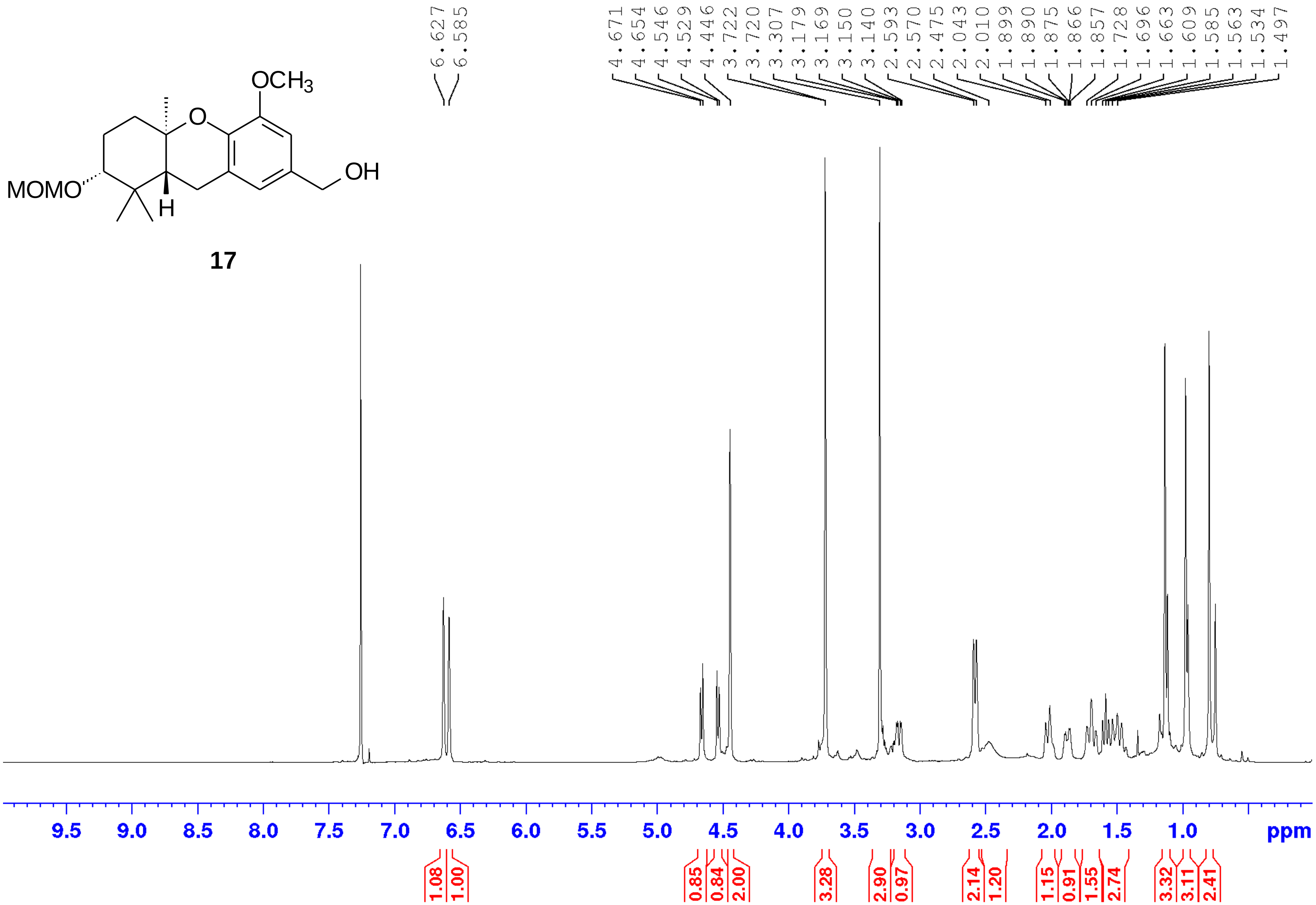

400 MHz ^{1}H NMR spectrum of **17** in $CDCl_3$

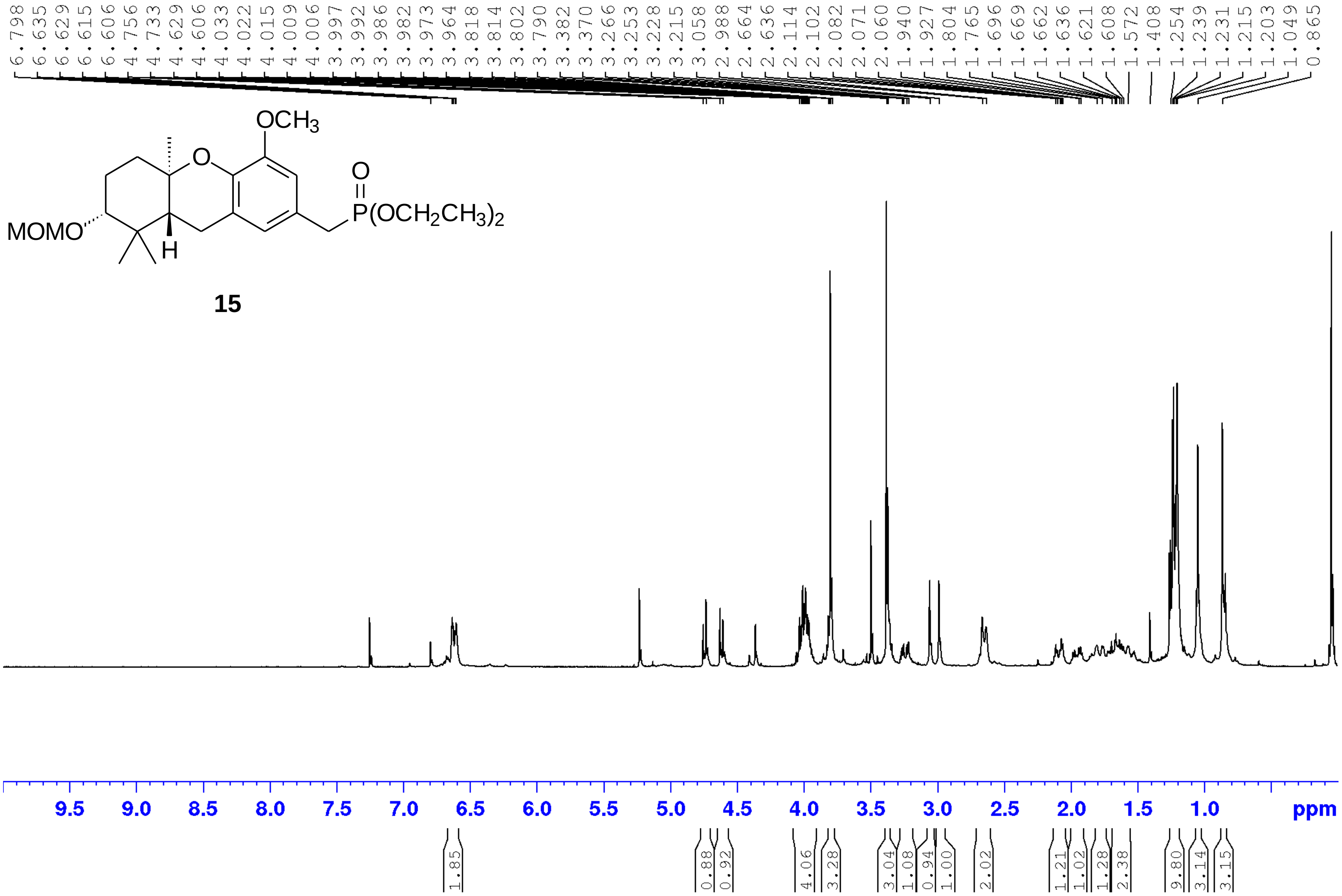

300 MHz ^{1}H NMR spectrum of **15** in $CDCl_3$

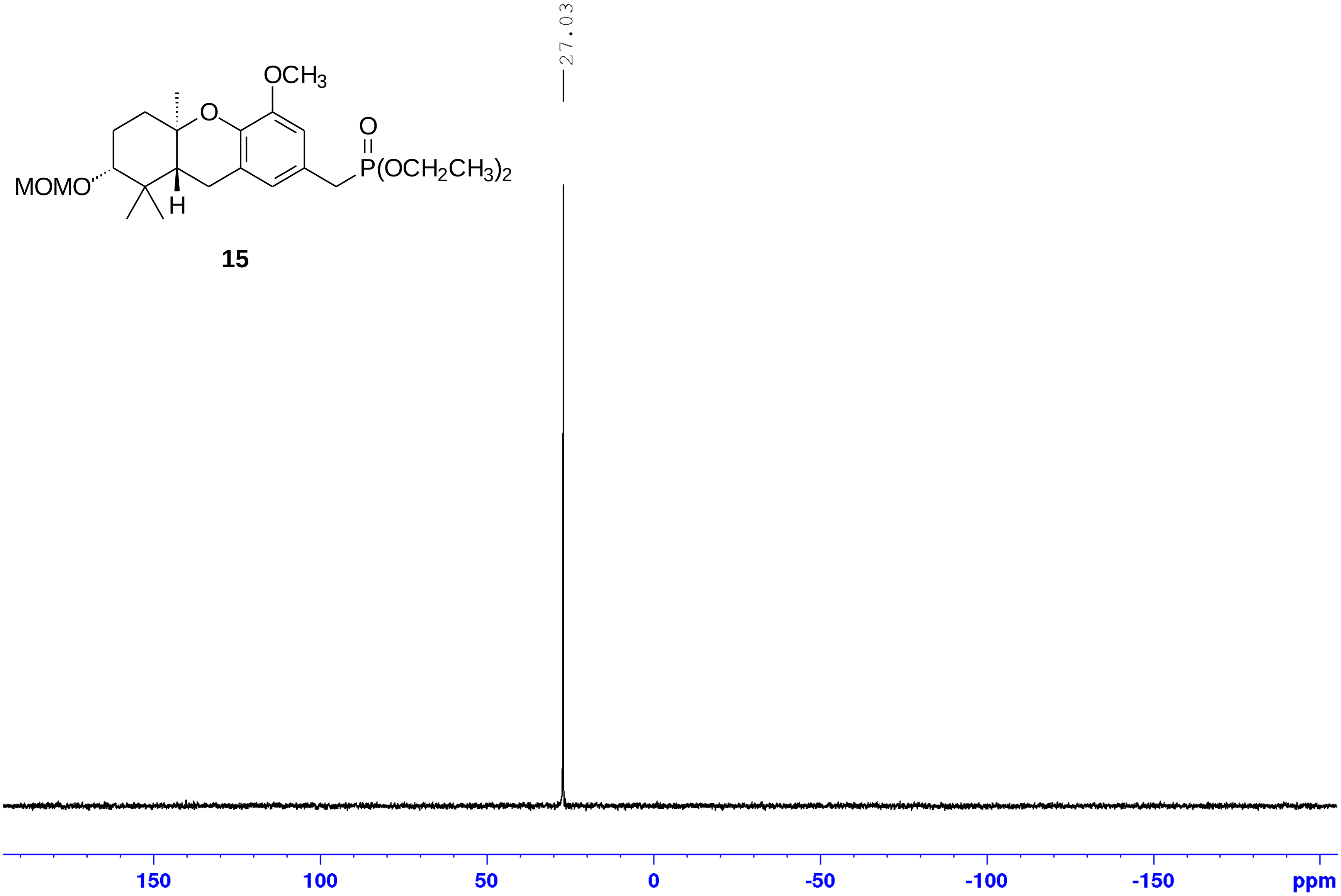

121 MHz ^{31}P NMR spectrum of **15** in $CDCl_3$

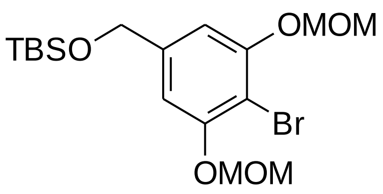

34

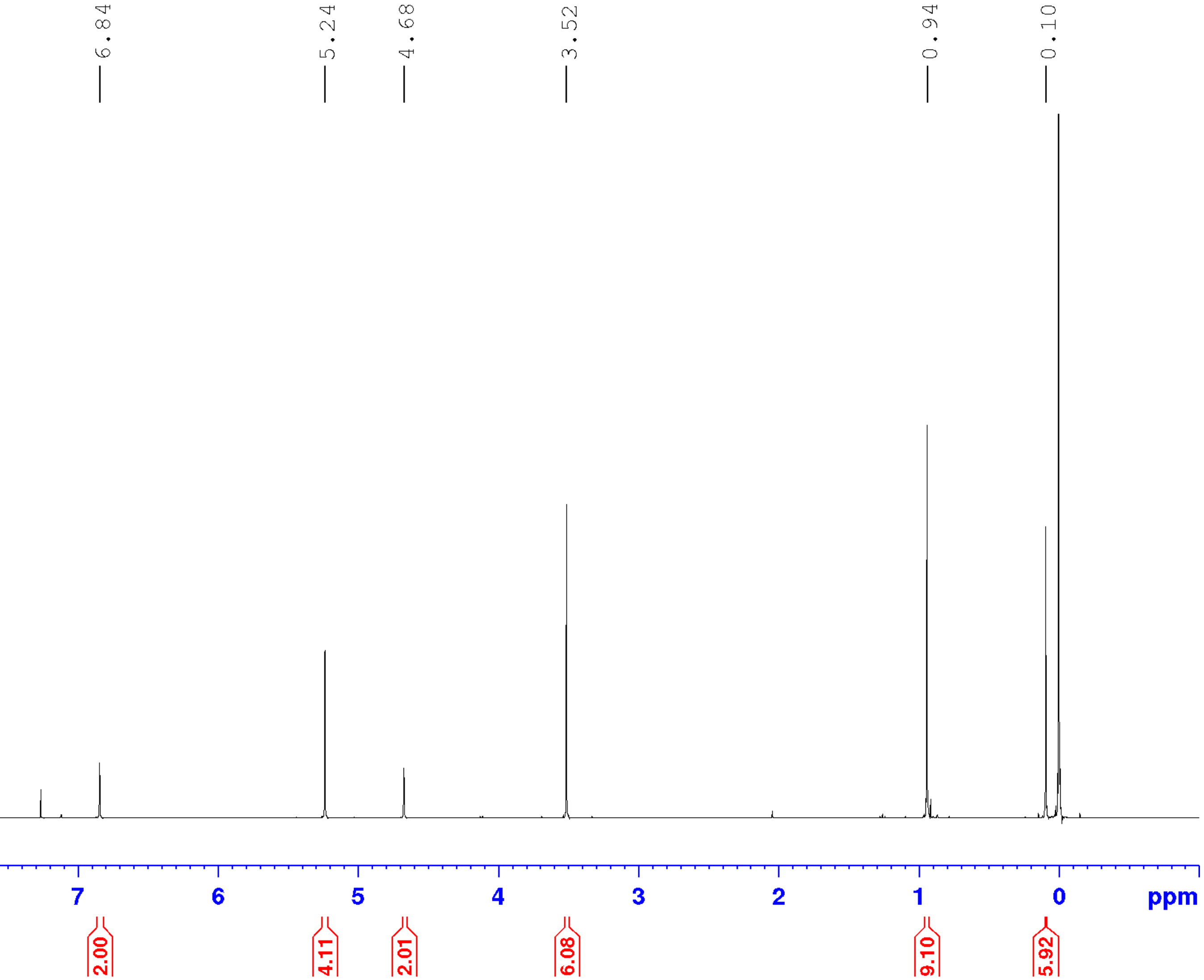

400 MHz ^{1}H NMR spectrum of **34** in $CDCl_3$

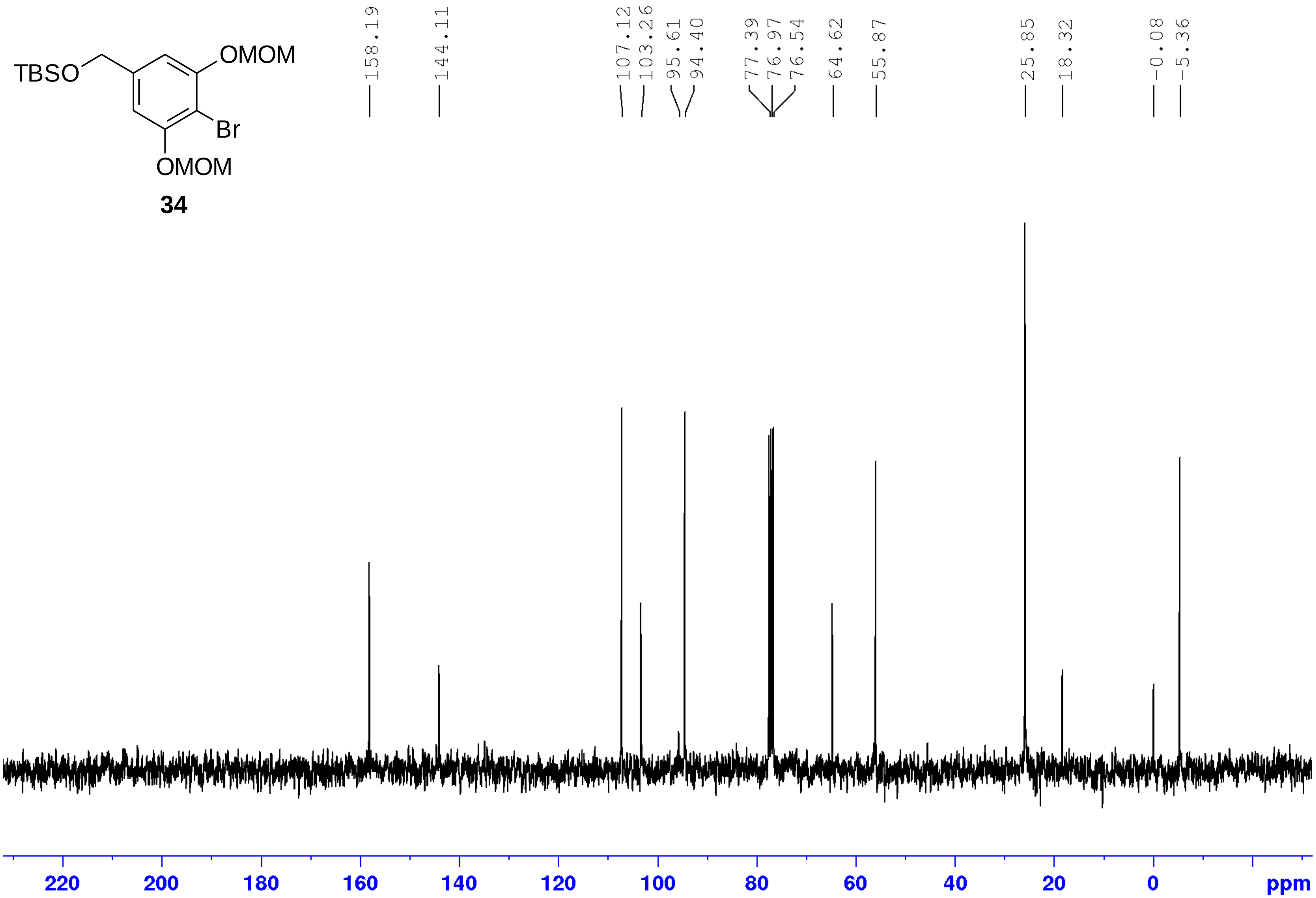

75 MHz $^{13}C\{^{1}H\}$ NMR spectrum of **34** in $CDCl_3$

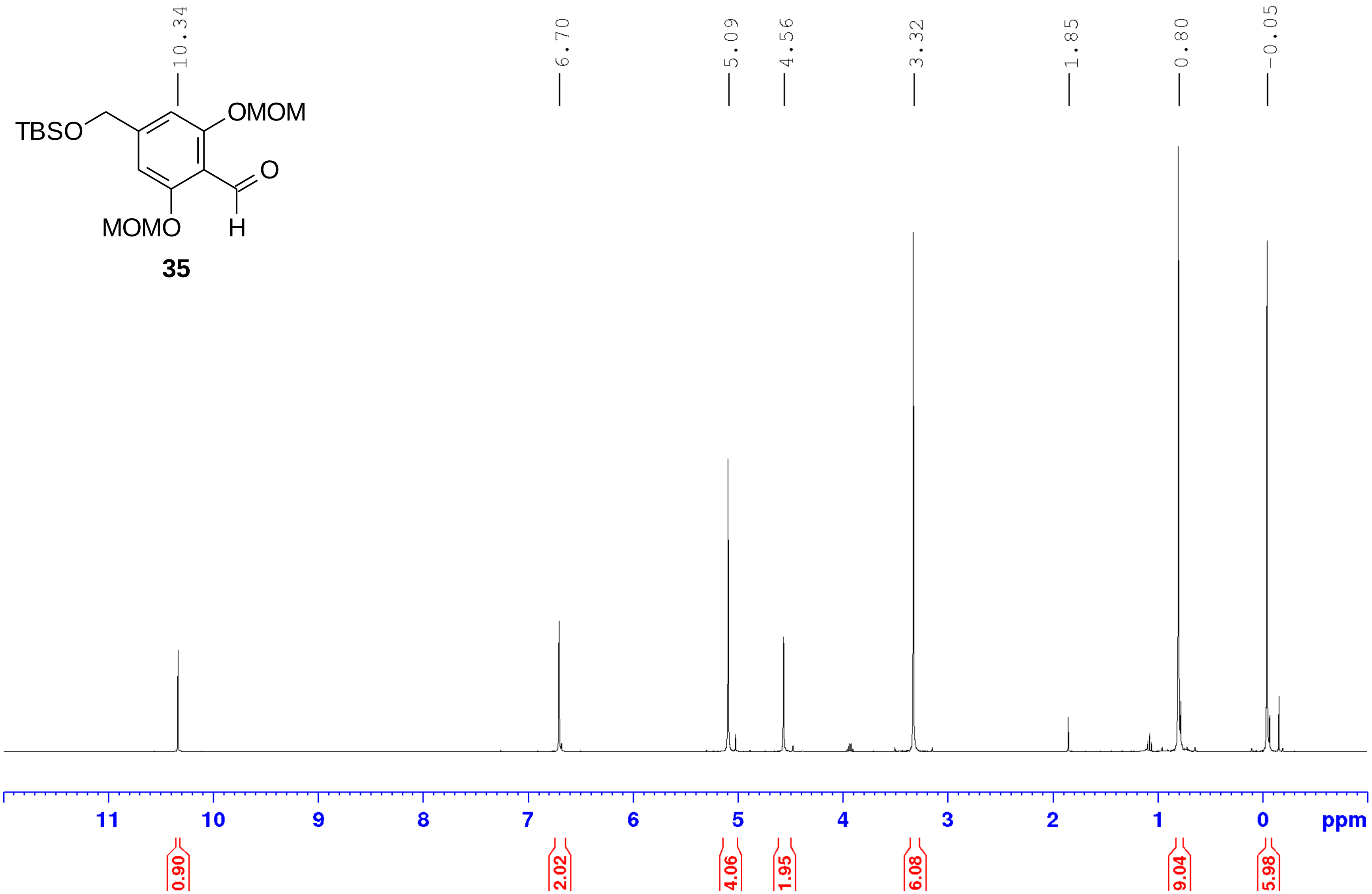

400 MHz ^{1}H NMR spectrum of **35** in $CDCl_3$

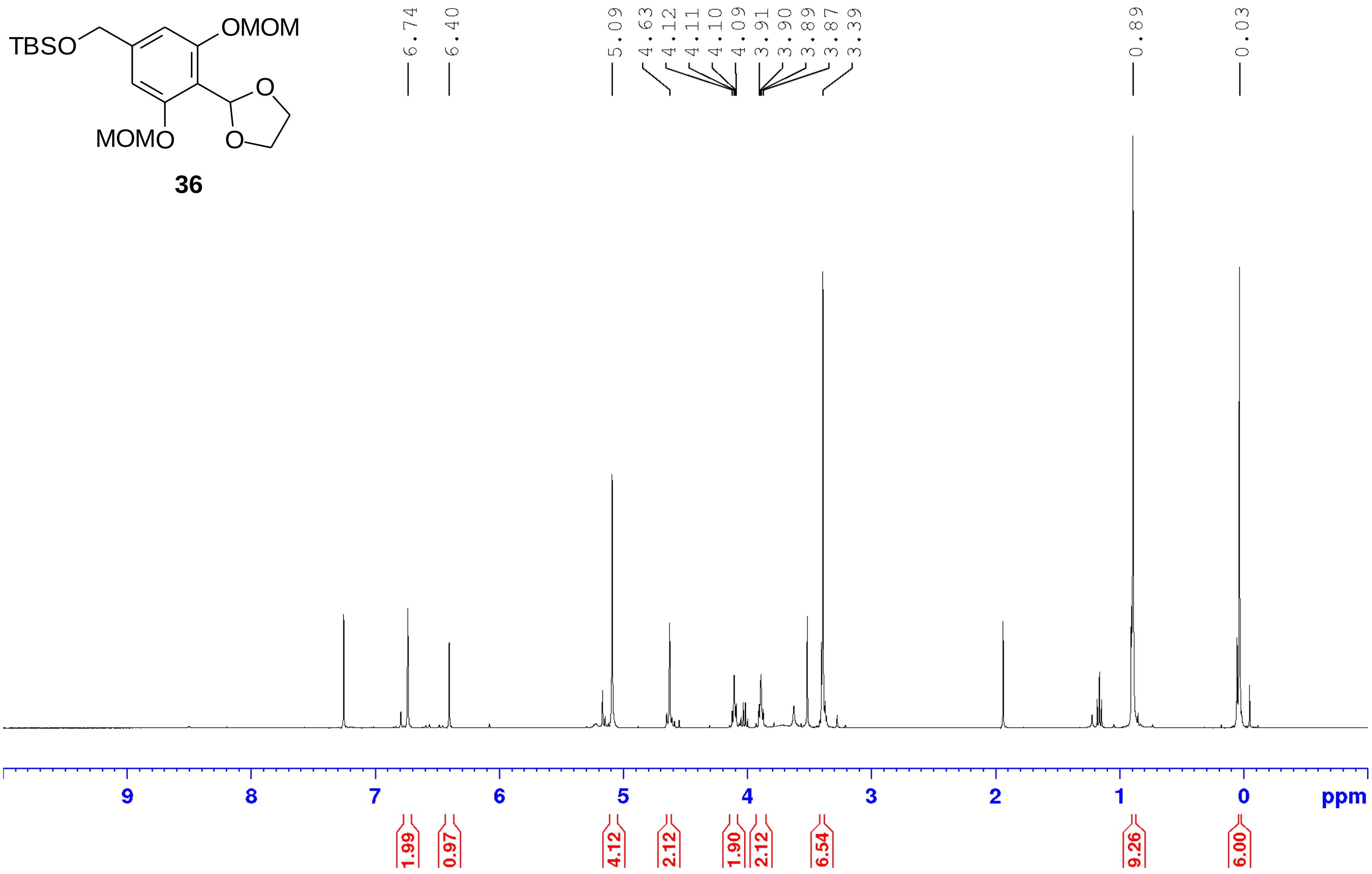

400 MHz ^{1}H NMR spectrum of **36** in $CDCl_3$

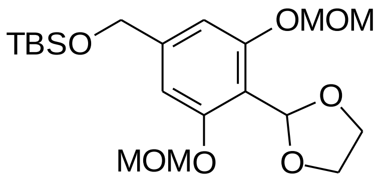

36

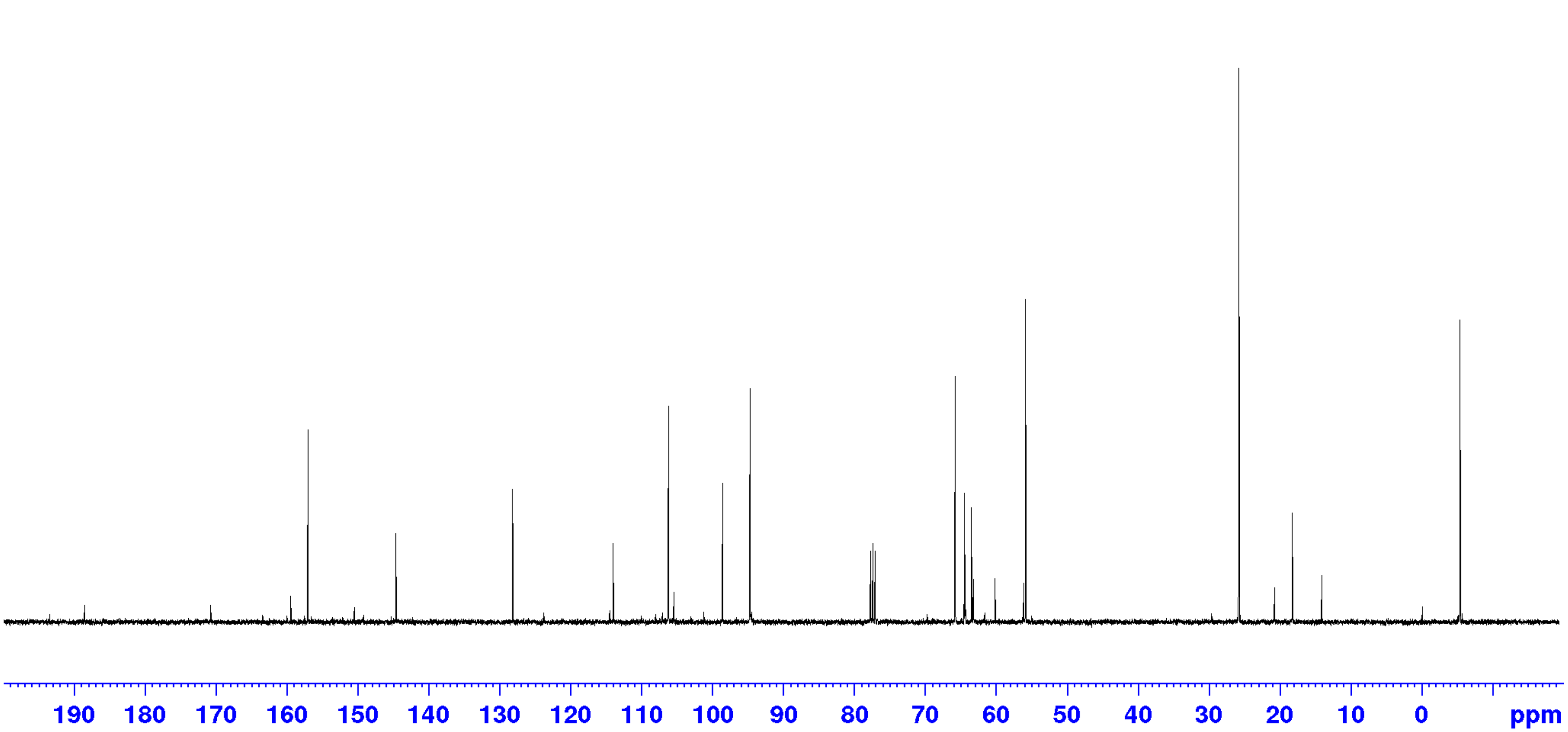

100 MHz $^{13}C\{^{1}H\}$ NMR spectrum of **36** in $CDCl_3$

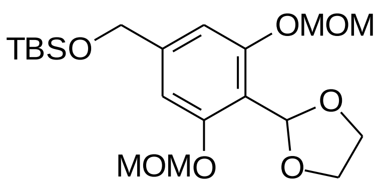

36

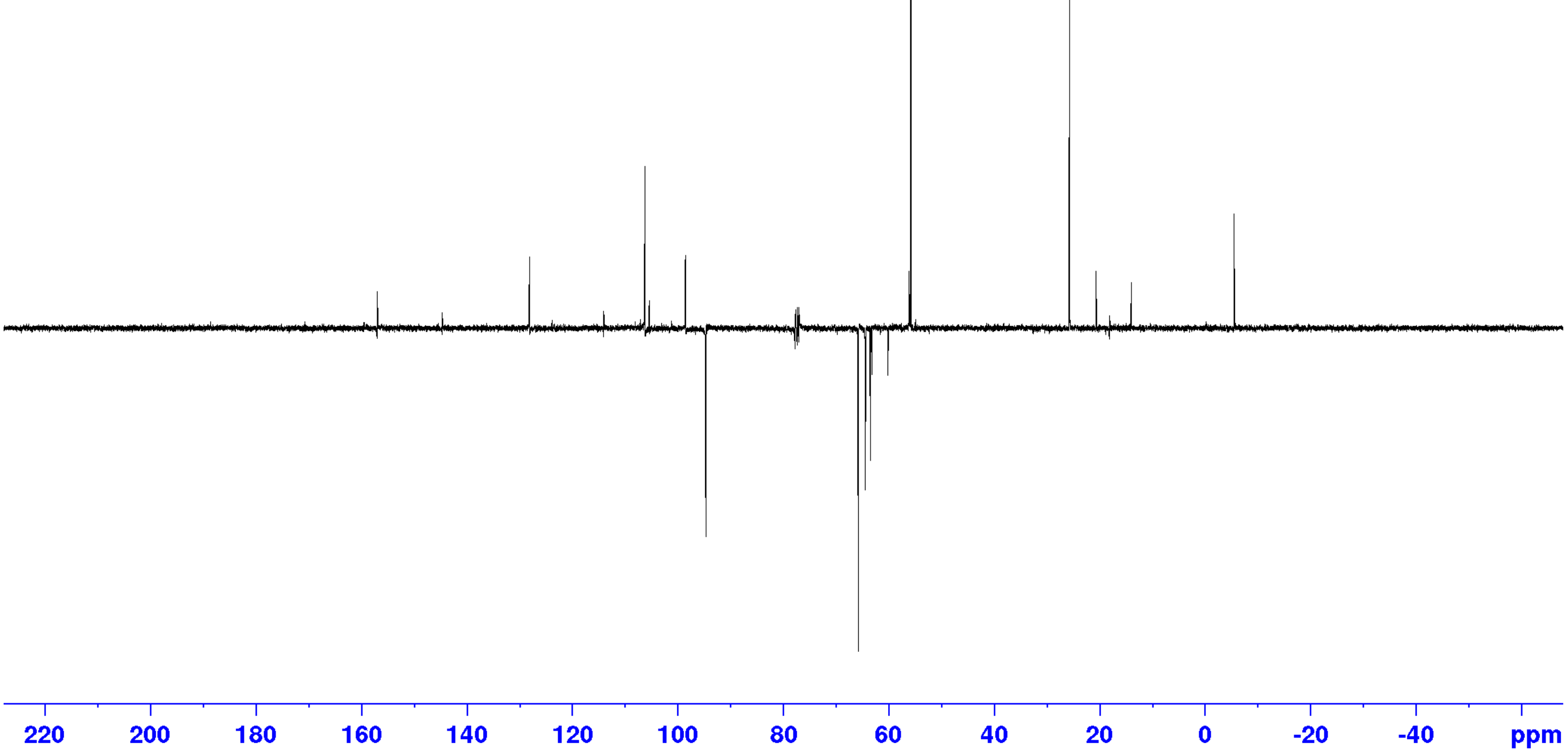

100 MHz DEPT-135 of **36** in $CDCl_3$

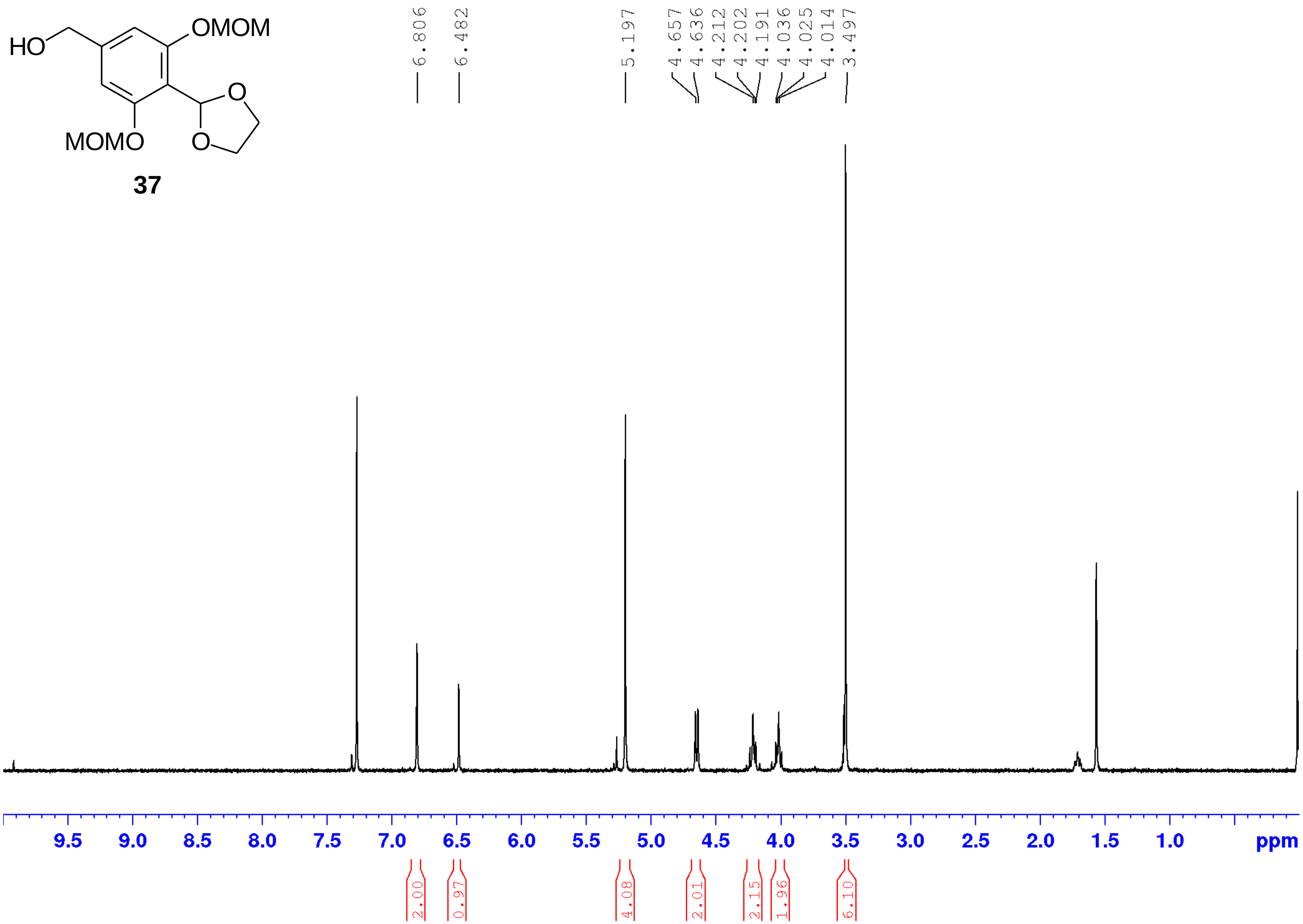

300 MHz ^{1}H NMR spectrum of **37** in $CDCl_3$

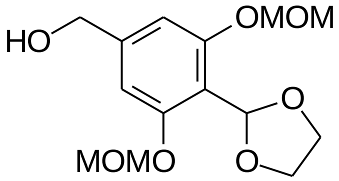

37

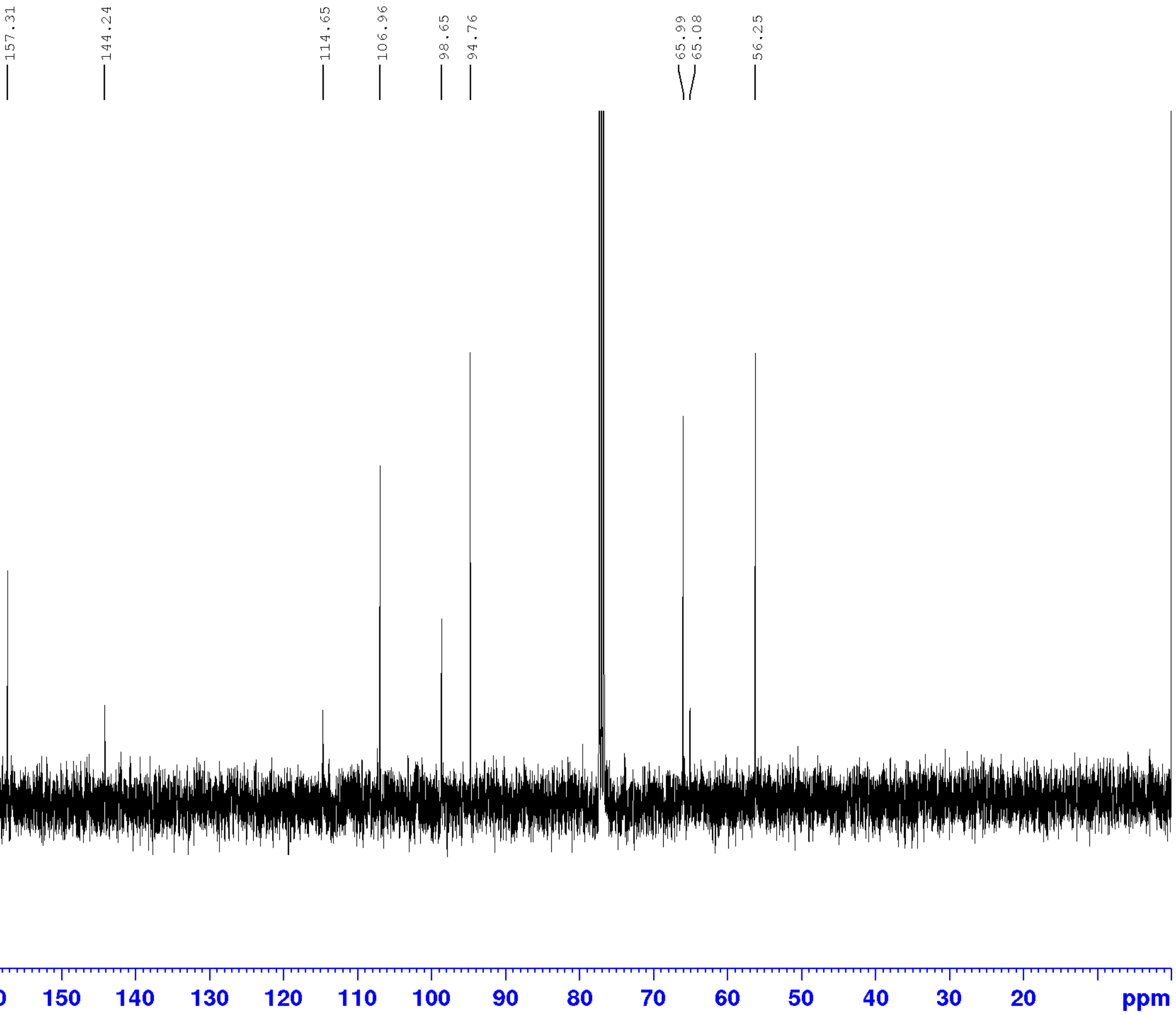

100 MHz ^{13}C{^{1}H} NMR spectrum of **37** in $CDCl_3$

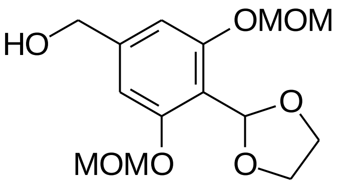

37

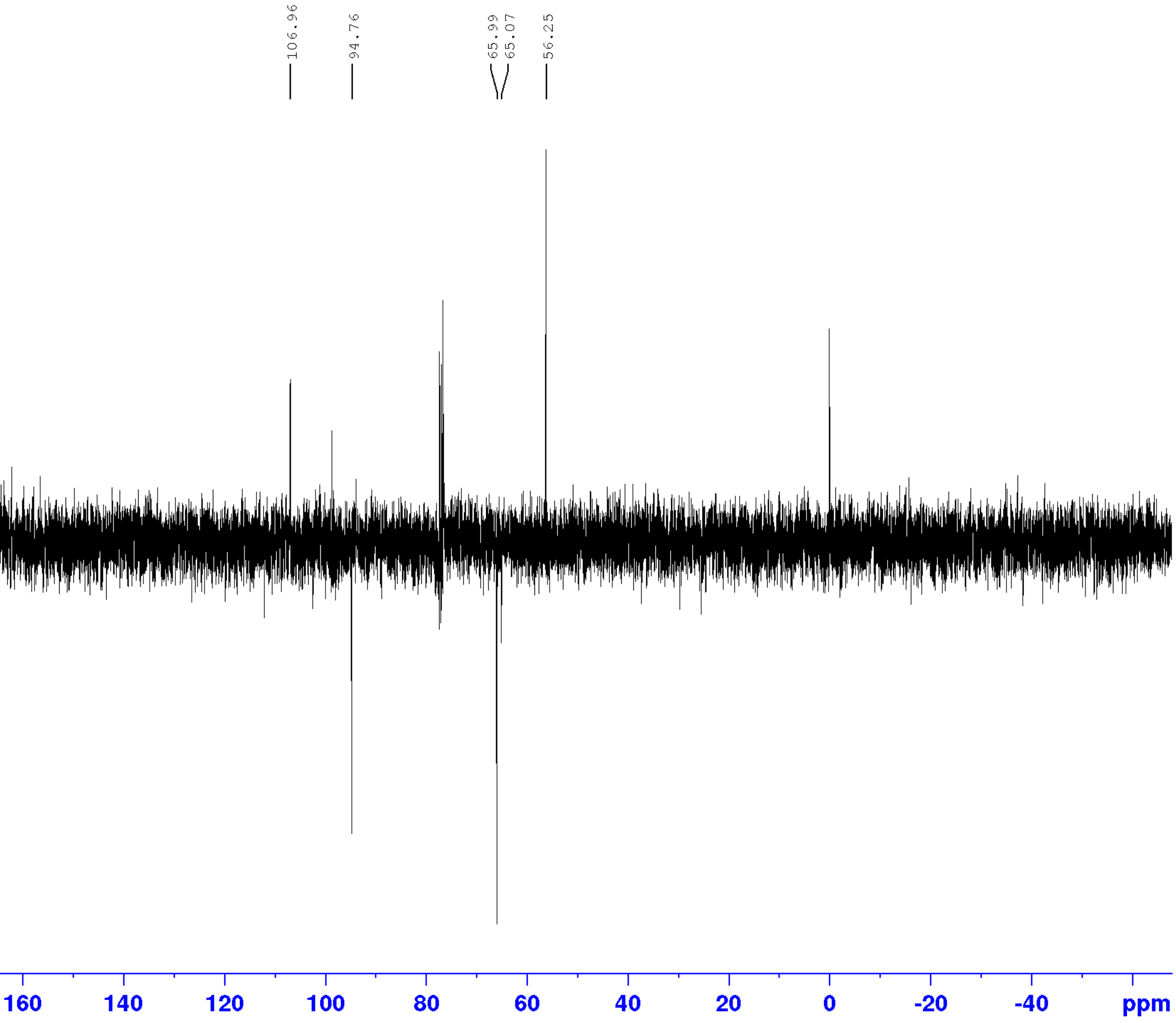

100 MHz DEPT-135 ^{13}C of **37** in $CDCl_3$

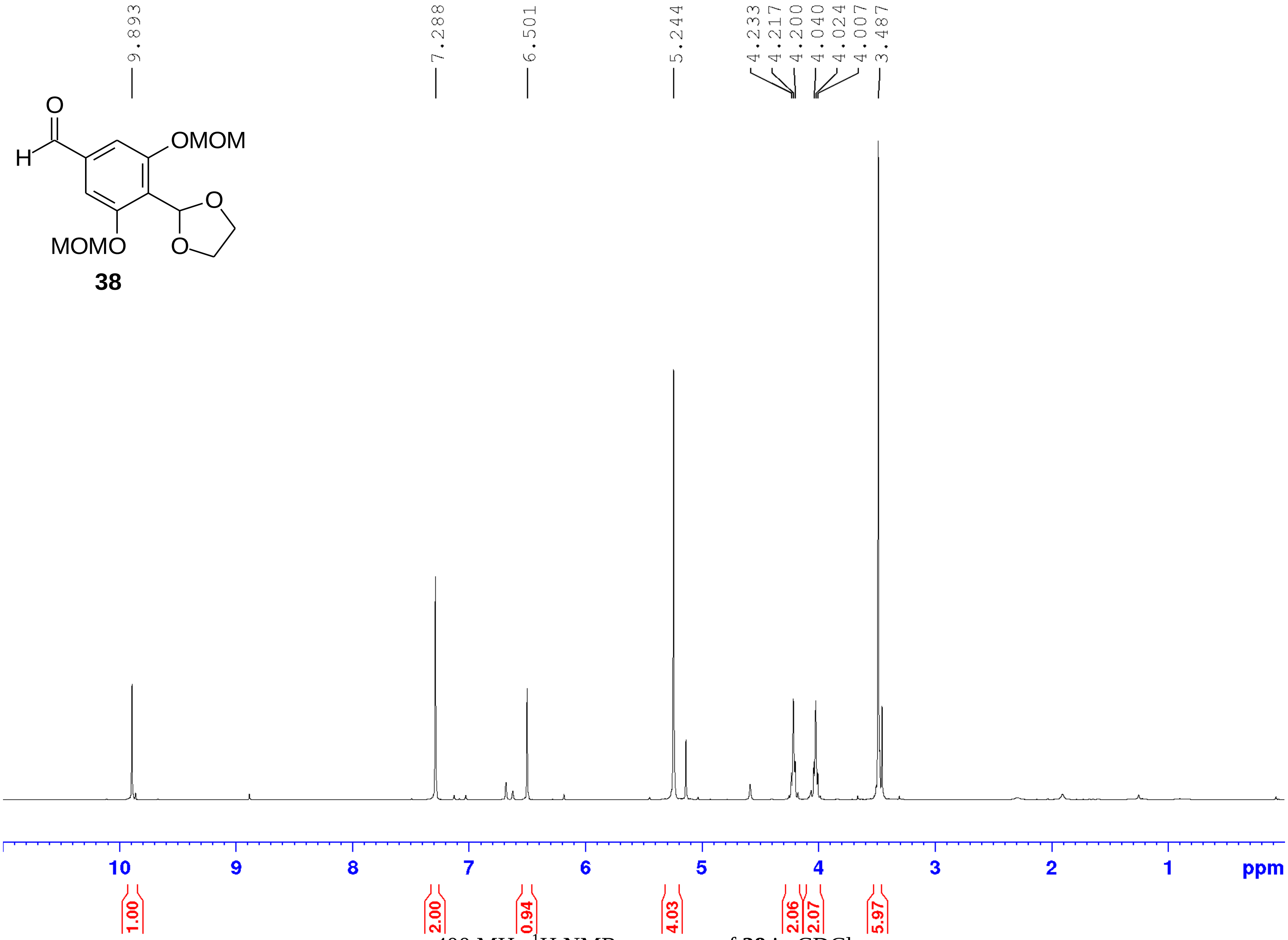

400 MHz ^{1}H NMR spectrum of **38** in $CDCl_3$

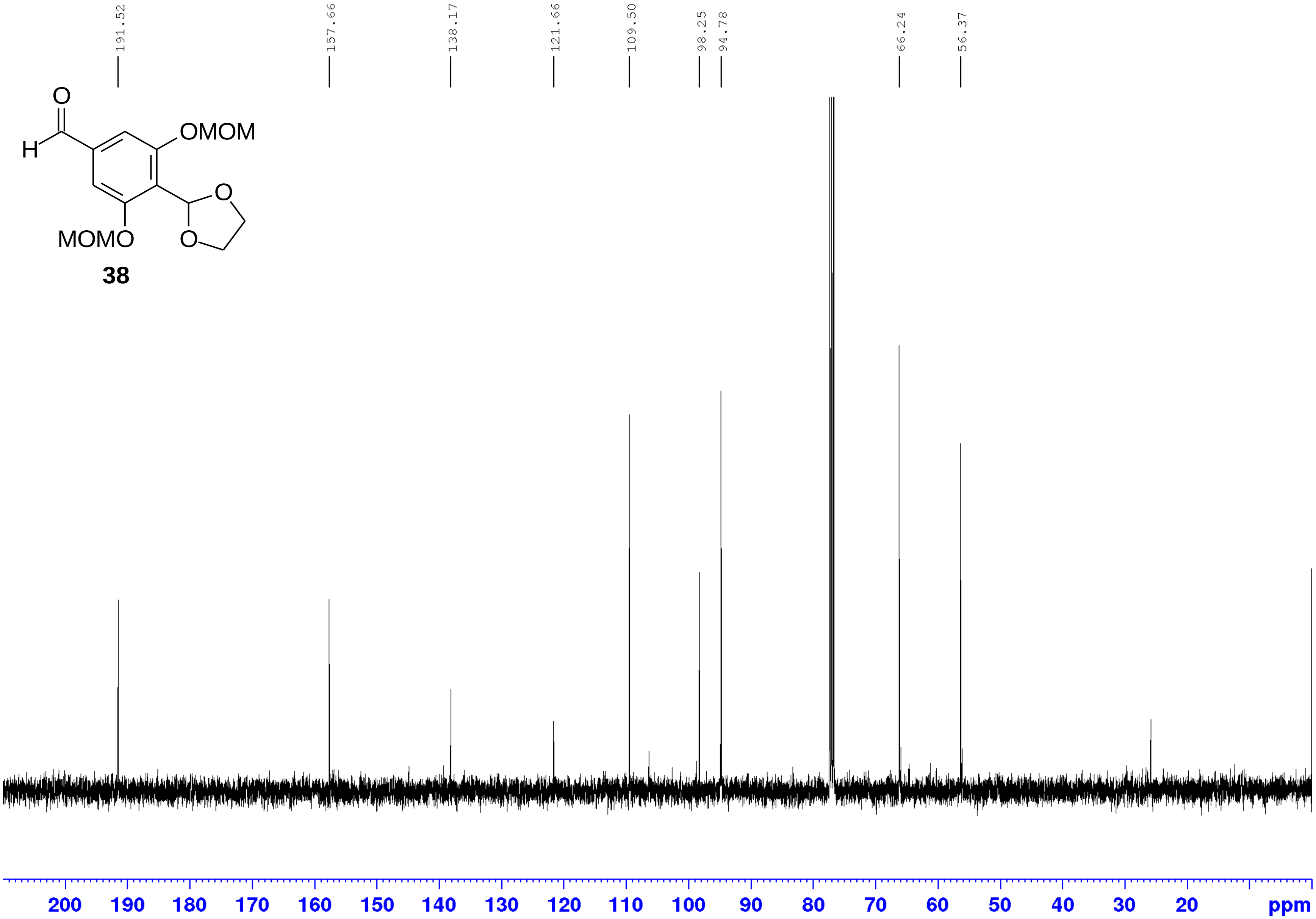

100 MHz $^{13}C\{^{1}H\}$ NMR spectrum of **38** in $CDCl_3$

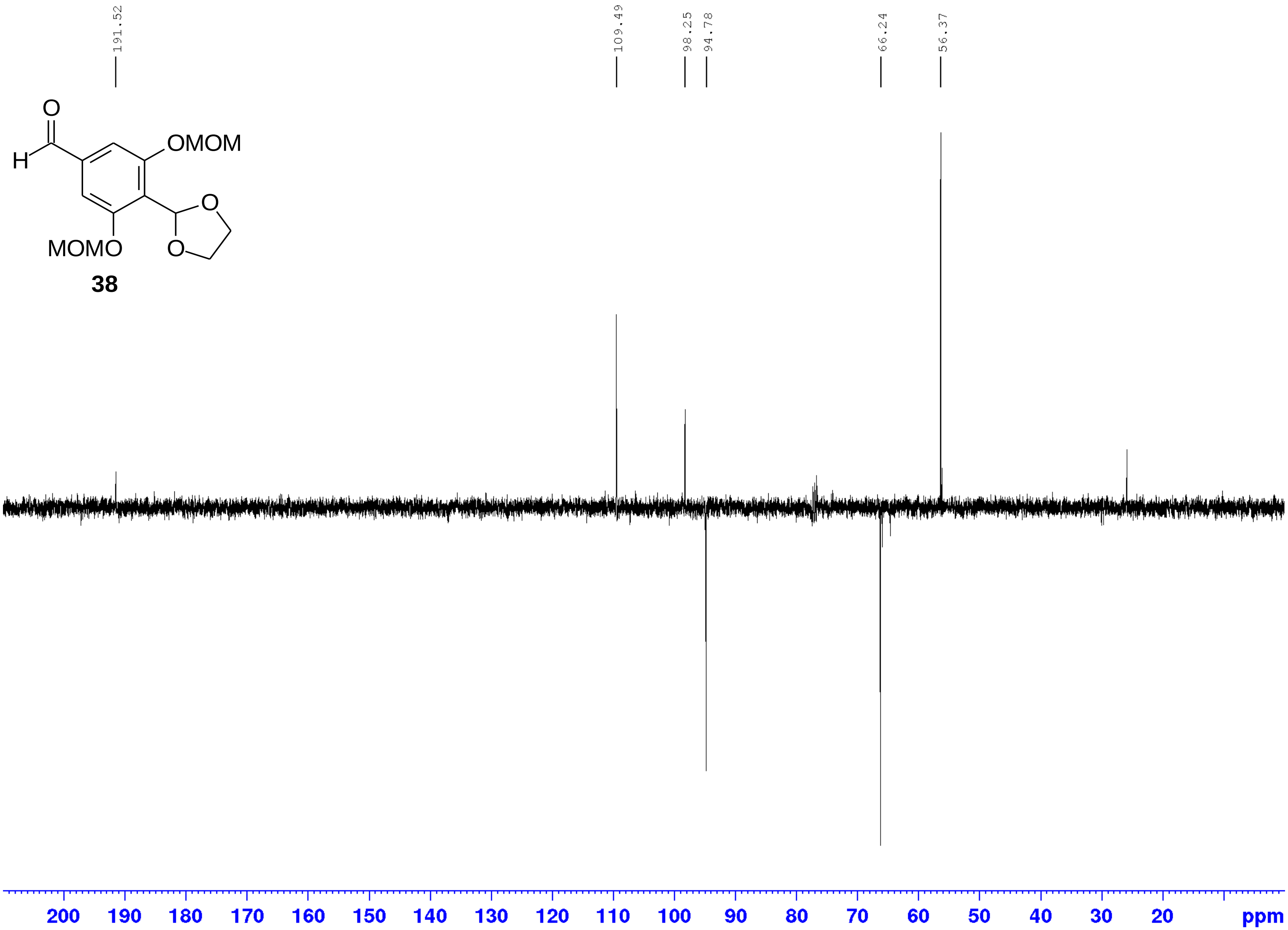

100 MHz DEPT-135 NMR spectrum of **38** in $CDCl_3$

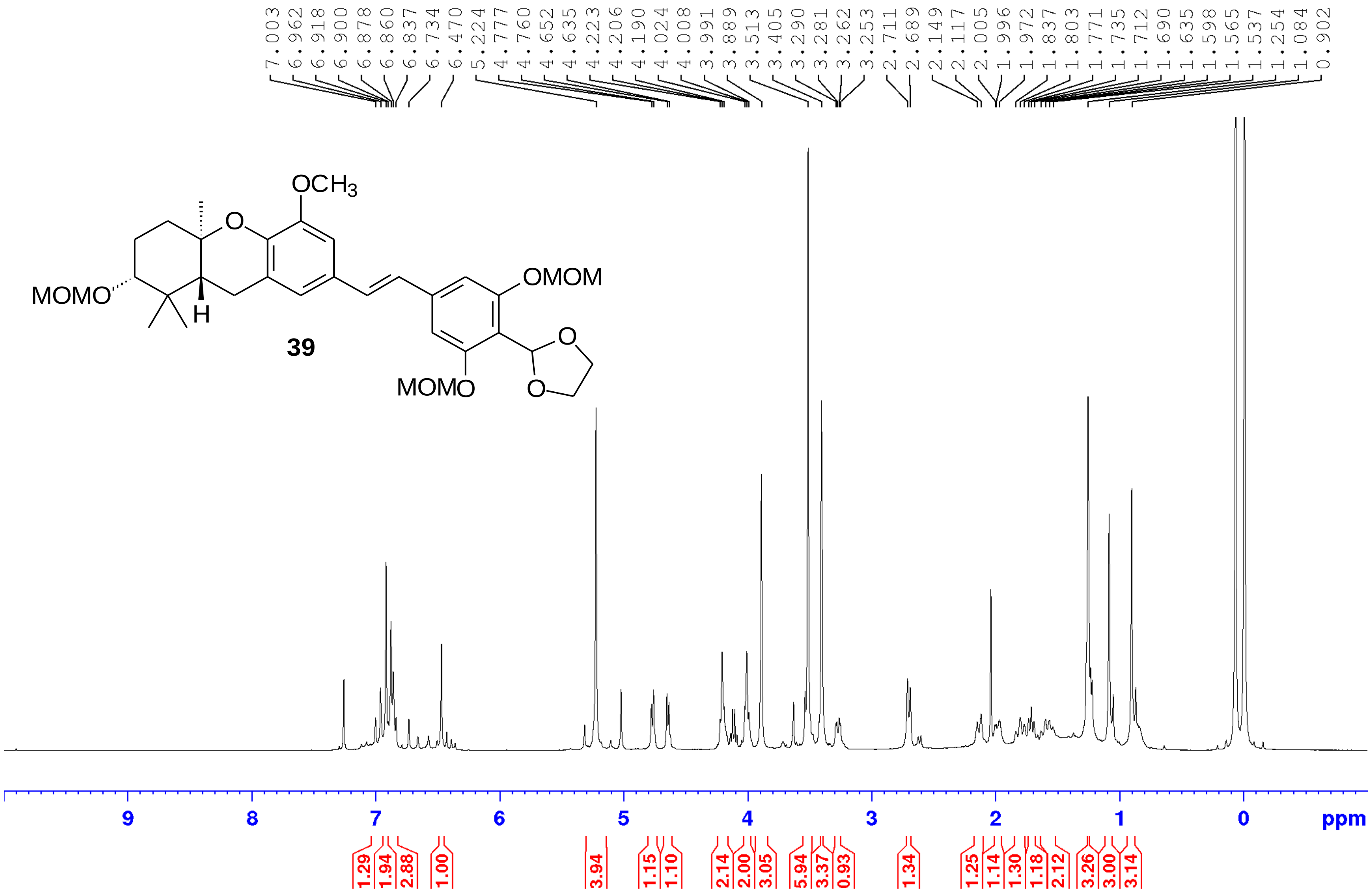

400 MHz ^1H NMR spectrum of **39** in $CDCl_3$

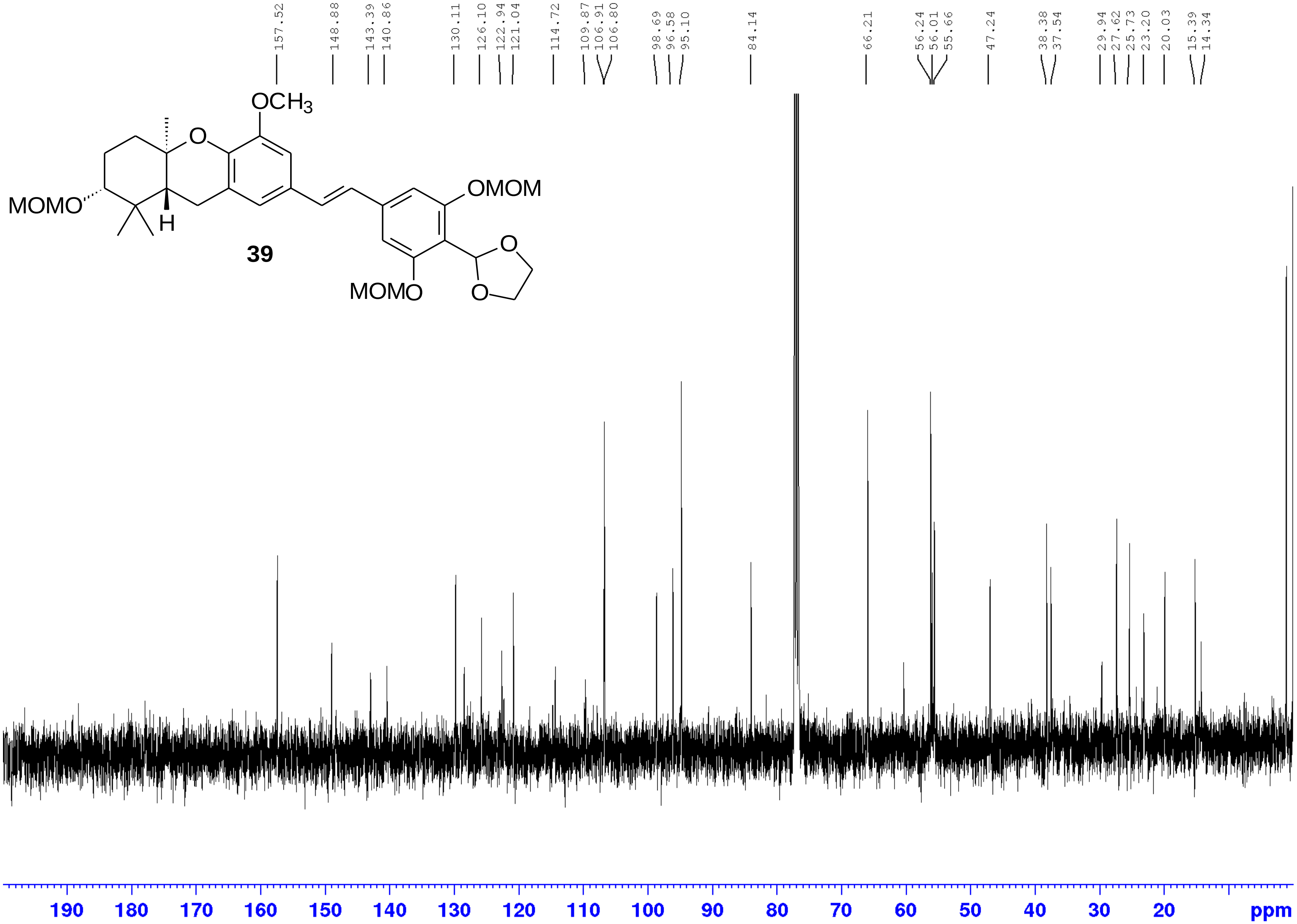

100 MHz 13C{^{1}H} NMR spectrum of **39** in $CDCl_3$

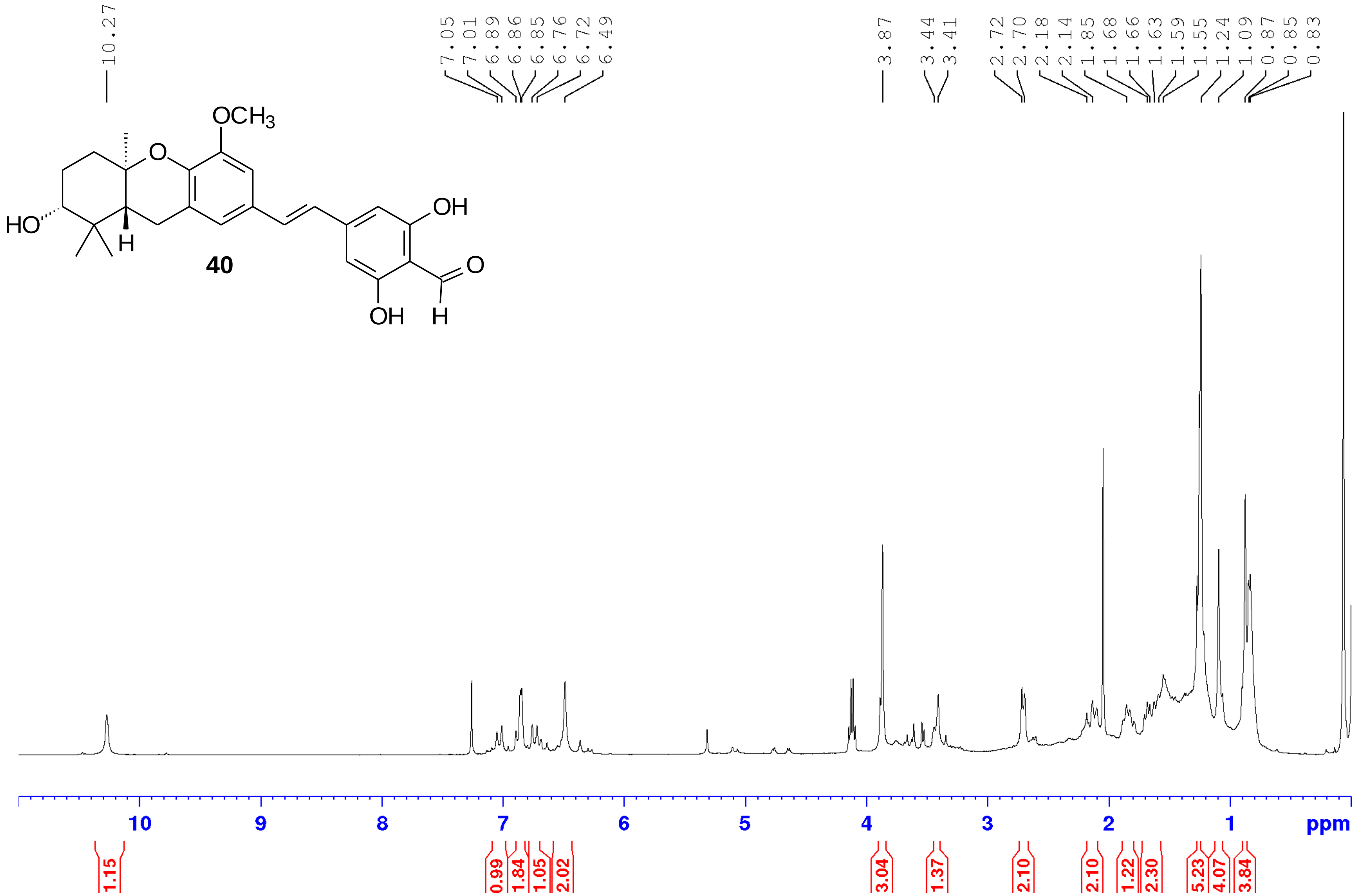

400 MHz ^{1}H NMR spectrum of **40** in $CDCl_3$

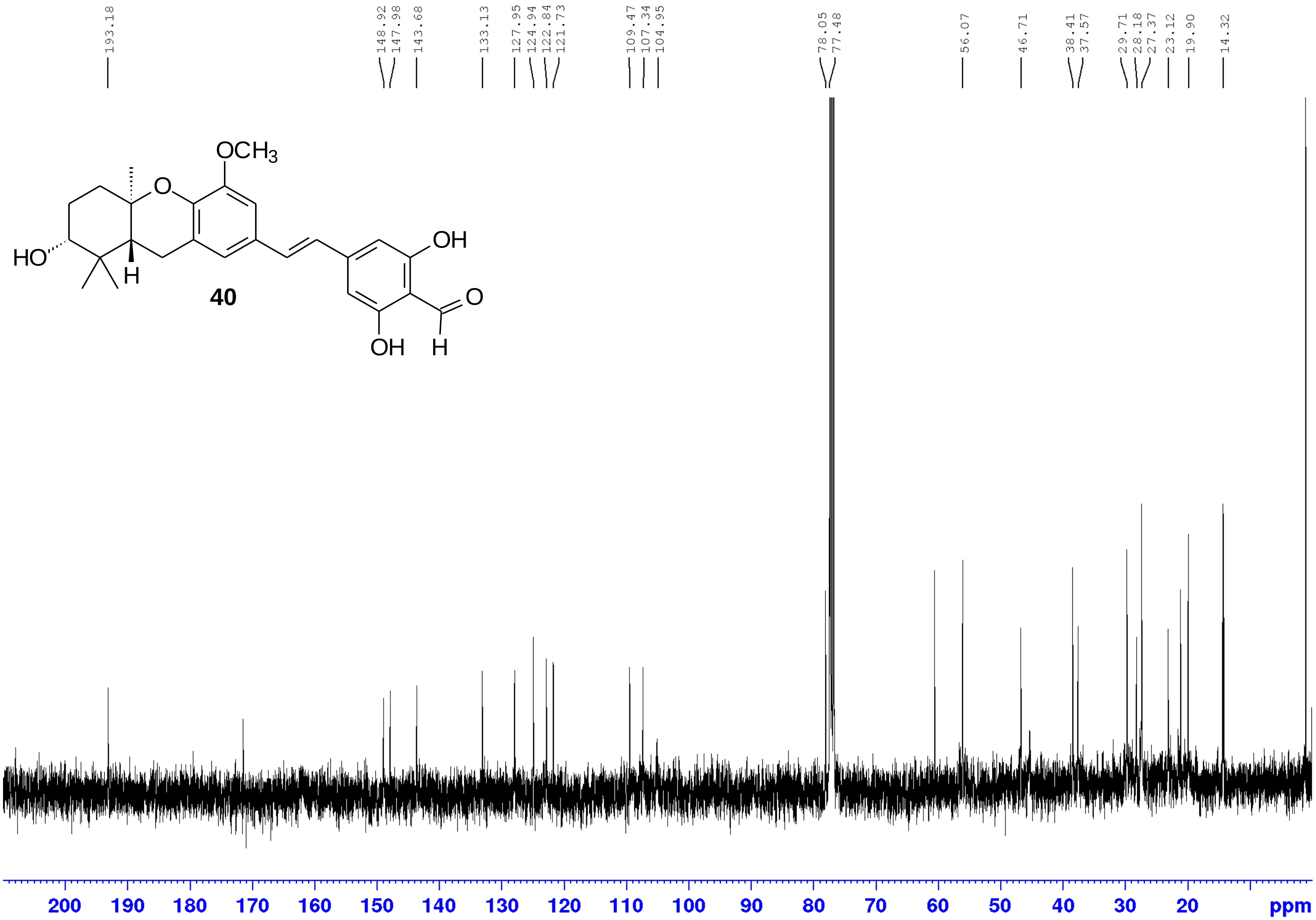

100 MHz $^{13}C\{^{1}H\}$ NMR spectrum of **40** in $CDCl_3$

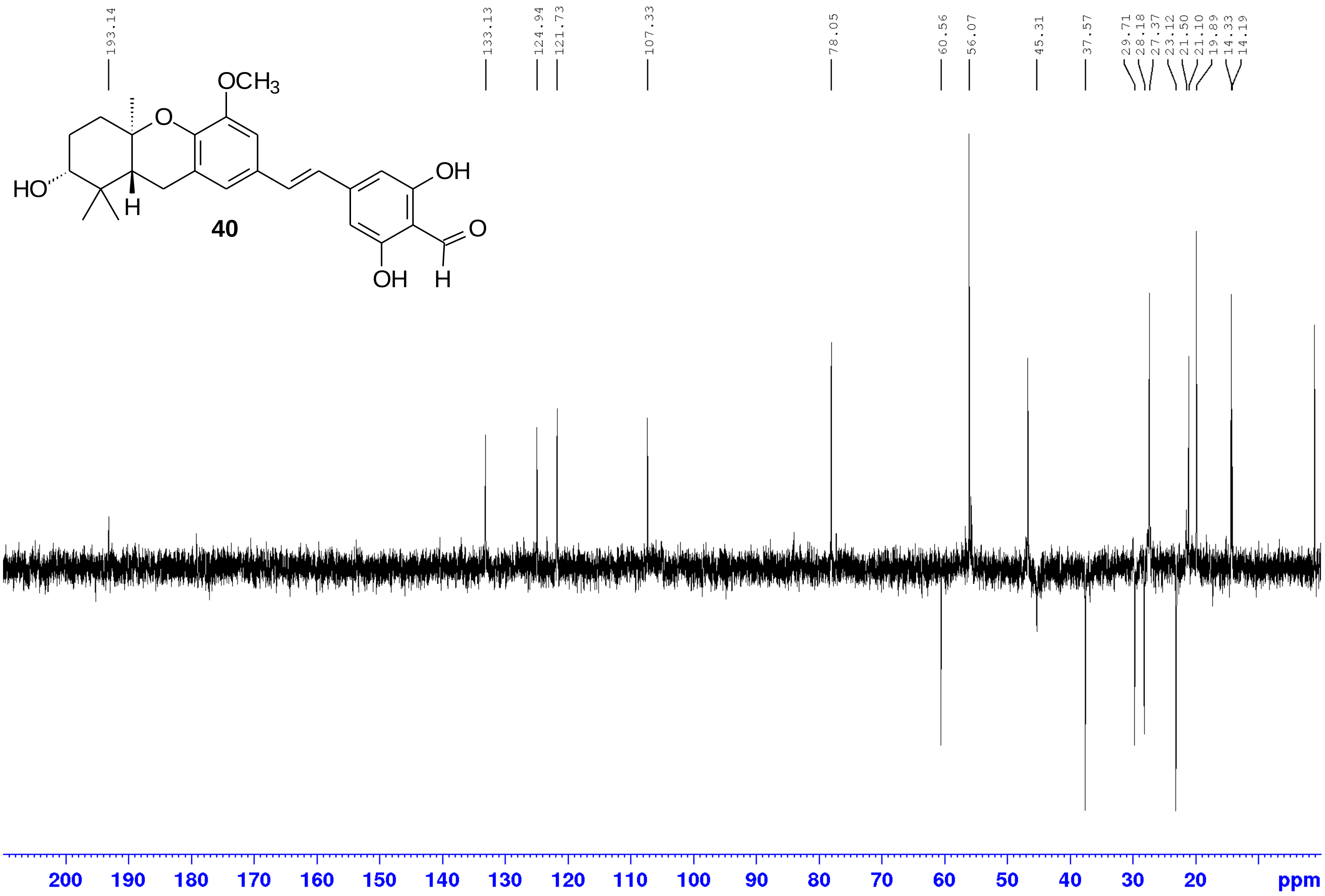

100 MHz DEPT-135 NMR spectrum of **40** in $CDCl_3$

National Cancer Institute Developmental Therapeutics Program
Dose Response Curves

NSC: D - 819974 / 1 | SSPL: 0GZS | EXP. ID: 1912NS94

Report Date: January 13, 2020 | Test Date: December 09, 2019

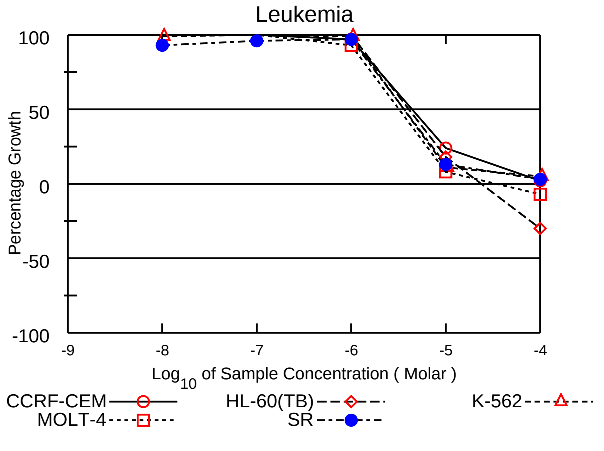

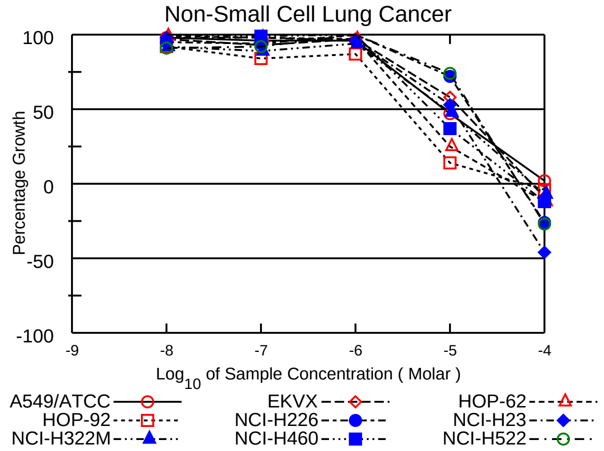

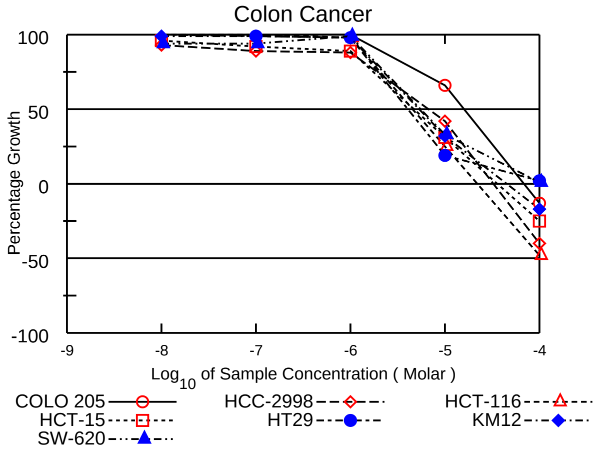

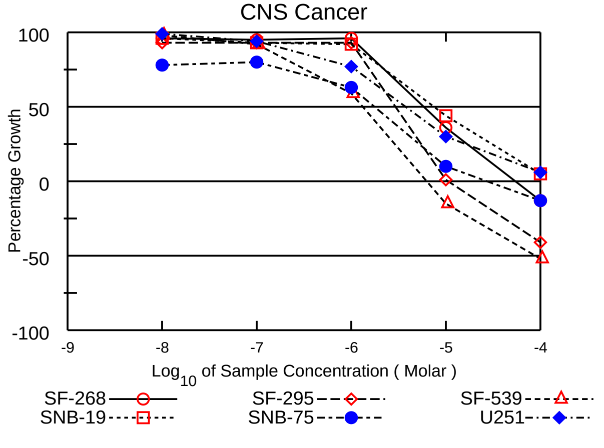

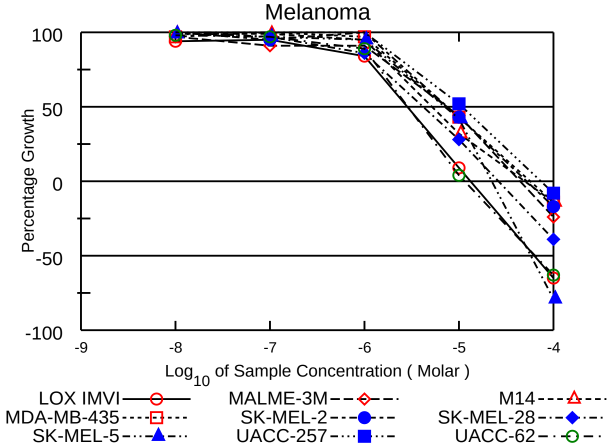

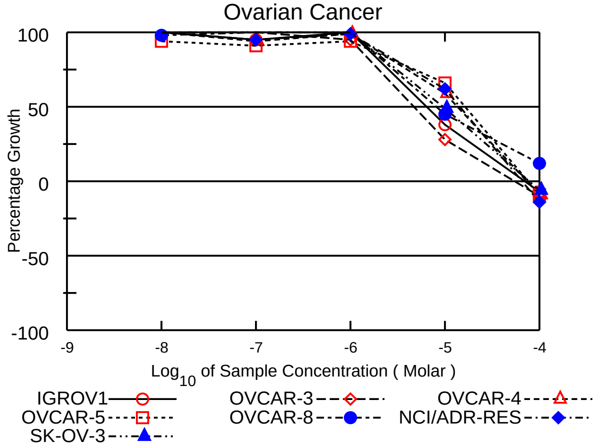

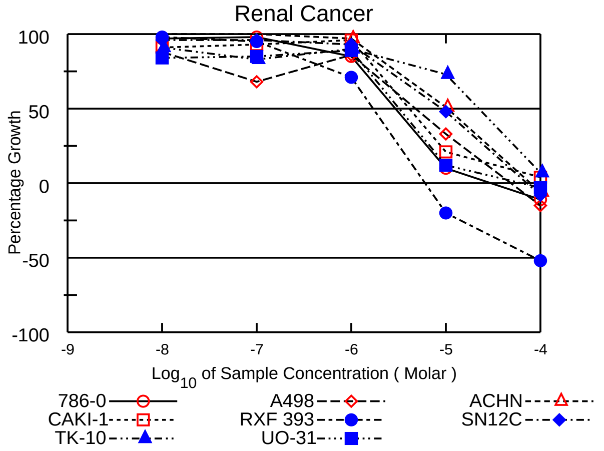

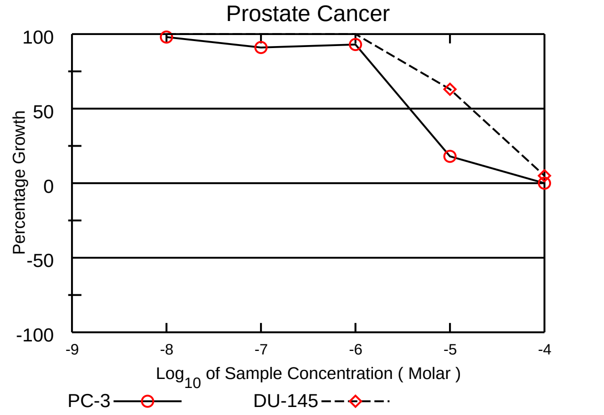

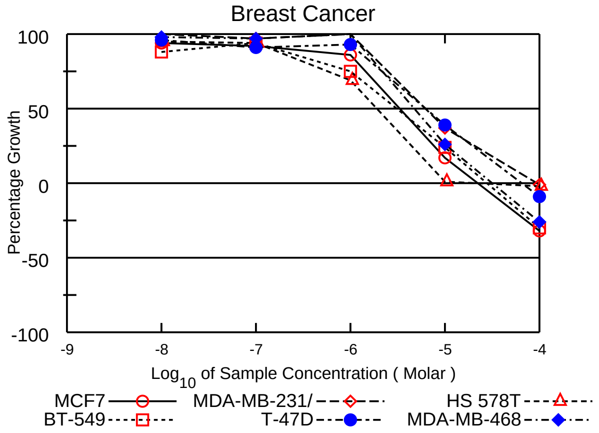

National Cancer Institute Developmental Therapeutics Program Mean Graphs	NSC : D - 819974/1	Units :Molar	SSPL :0GZS	EXP. ID :1912NS94
	Report Date :January 13, 2020		Test Date :December 09, 2019	

Panel/Cell Line	Log_{10}GI50	Log_{10}TGI	Log_{10}LC50
**Leukemia**			
CCRF-CEM	-5.36	> -4.00	> -4.00
HL-60(TB)	-5.38	-4.62	> -4.00
K-562	-5.44	> -4.00	> -4.00
MOLT-4	-5.49	-4.46	> -4.00
SR	-5.44	> -4.00	> -4.00
**Non-Small Cell Lung Cancer**			
A549/ATCC	-5.07	> -4.00	> -4.00
EKVX	-4.88	-4.14	> -4.00
HOP-62	-5.34	-4.32	> -4.00
HOP-92	-5.50	-4.21	> -4.00
NCI-H226	-4.77	-4.27	> -4.00
NCI-H23	-4.97	-4.46	> -4.00
NCI-H322M	-5.03	-4.13	> -4.00
NCI-H460	-5.20	-4.24	> -4.00
NCI-H522	-4.76	-4.27	> -4.00
**Colon Cancer**			
COLO 205	-4.79	-4.17	> -4.00
HCC-2998	-5.17	-4.49	> -4.00
HCT-116	-5.34	-4.66	> -4.00
HCT-15	-5.33	-4.45	> -4.00
HT29	-5.39	> -4.00	> -4.00
KM12	-5.27	-4.34	> -4.00
SW-620	-5.26	> -4.00	> -4.00
**CNS Cancer**			
SF-268	-5.23	-4.26	> -4.00
SF-295	-5.53	-4.98	> -4.00
SF-539	-5.88	-5.21	-4.06
SNB-19	-5.13	> -4.00	> -4.00
SNB-75	-5.75	-4.57	> -4.00
U251	-5.43	> -4.00	> -4.00
**Melanoma**			
LOX IMVI	-5.55	-4.88	-4.21
MALME-3M	-5.13	-4.35	> -4.00
M14	-5.29	-4.31	> -4.00
MDA-MB-435	-5.13	-4.24	> -4.00
SK-MEL-2	-5.10	-4.29	> -4.00
SK-MEL-28	-5.38	-4.58	> -4.00
SK-MEL-5	-5.15	-4.65	-4.24
UACC-257	-4.96	-4.14	> -4.00
UACC-62	-5.54	-4.94	-4.20
**Ovarian Cancer**			
IGROV1	-5.19	-4.16	> -4.00
OVCAR-3	-5.33	-4.27	> -4.00
OVCAR-4	-4.87	-4.13	> -4.00
OVCAR-5	-4.79	-4.12	> -4.00
OVCAR-8	-5.08	> -4.00	> -4.00
NCI/ADR-RES	-4.84	-4.18	> -4.00
SK-OV-3	-5.02	-4.10	> -4.00
**Renal Cancer**			
786-0	-5.54	-4.54	> -4.00
A498	-5.32	-4.32	> -4.00
ACHN	-4.98	-4.11	> -4.00
CAKI-1	-5.39	> -4.00	> -4.00
RXF 393	-5.77	-5.22	-4.07
SN12C	-5.04	-4.14	> -4.00
TK-10	-4.65	> -4.00	> -4.00
UO-31	-5.49	-4.18	> -4.00
**Prostate Cancer**			
PC-3	-5.43	> -4.00	> -4.00
DU-145	-4.78	> -4.00	> -4.00
**Breast Cancer**			
MCF7	-5.48	-4.65	> -4.00
MDA-MB-231/ATCC	-5.20	-4.03	> -4.00
HS 578T	-5.73	-4.79	> -4.00
BT-549	-5.52	-4.56	> -4.00
T-47D	-5.21	-4.19	> -4.00
MDA-MB-468	-5.32	-4.51	> -4.00
_MID	-5.24	-4.32	-4.01
Delta	0.64	0.9	0.23
Range	1.23	1.22	0.24

GI50

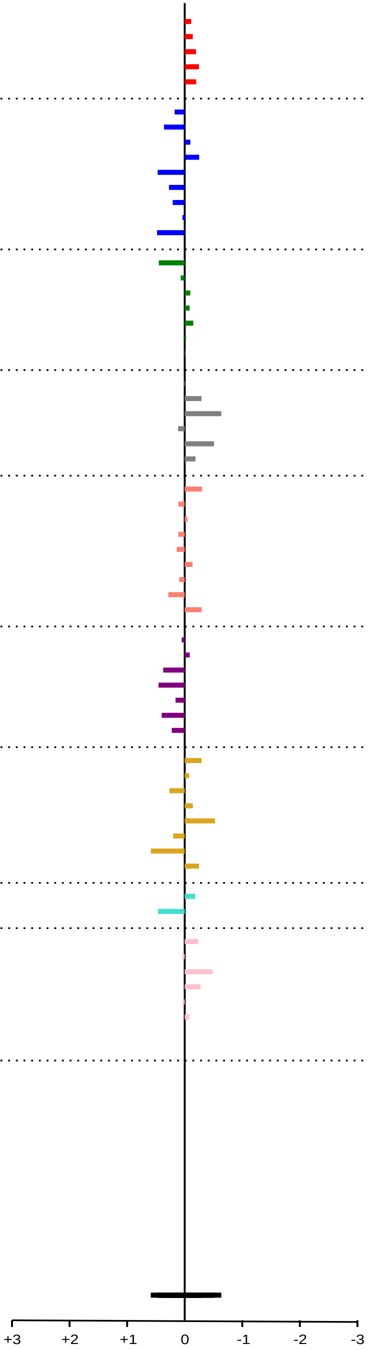

TGI

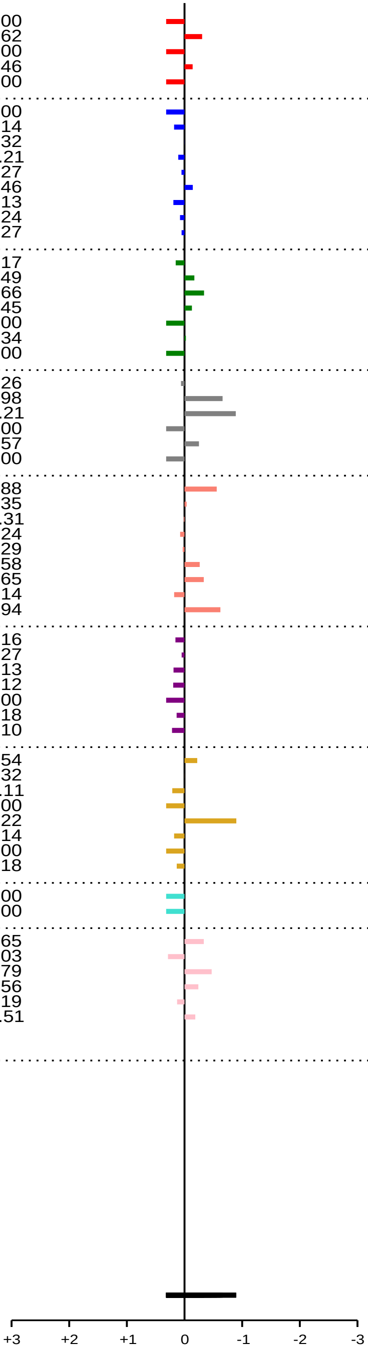

LC50

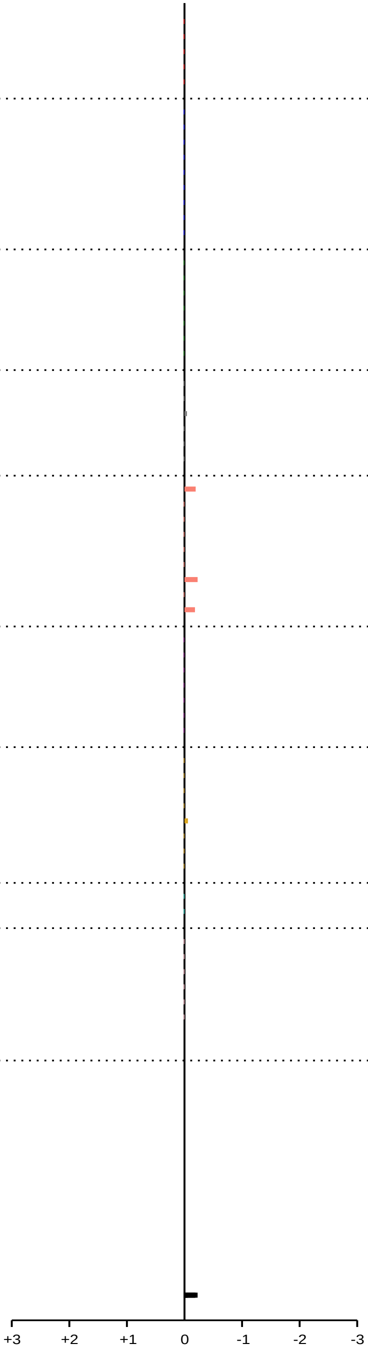

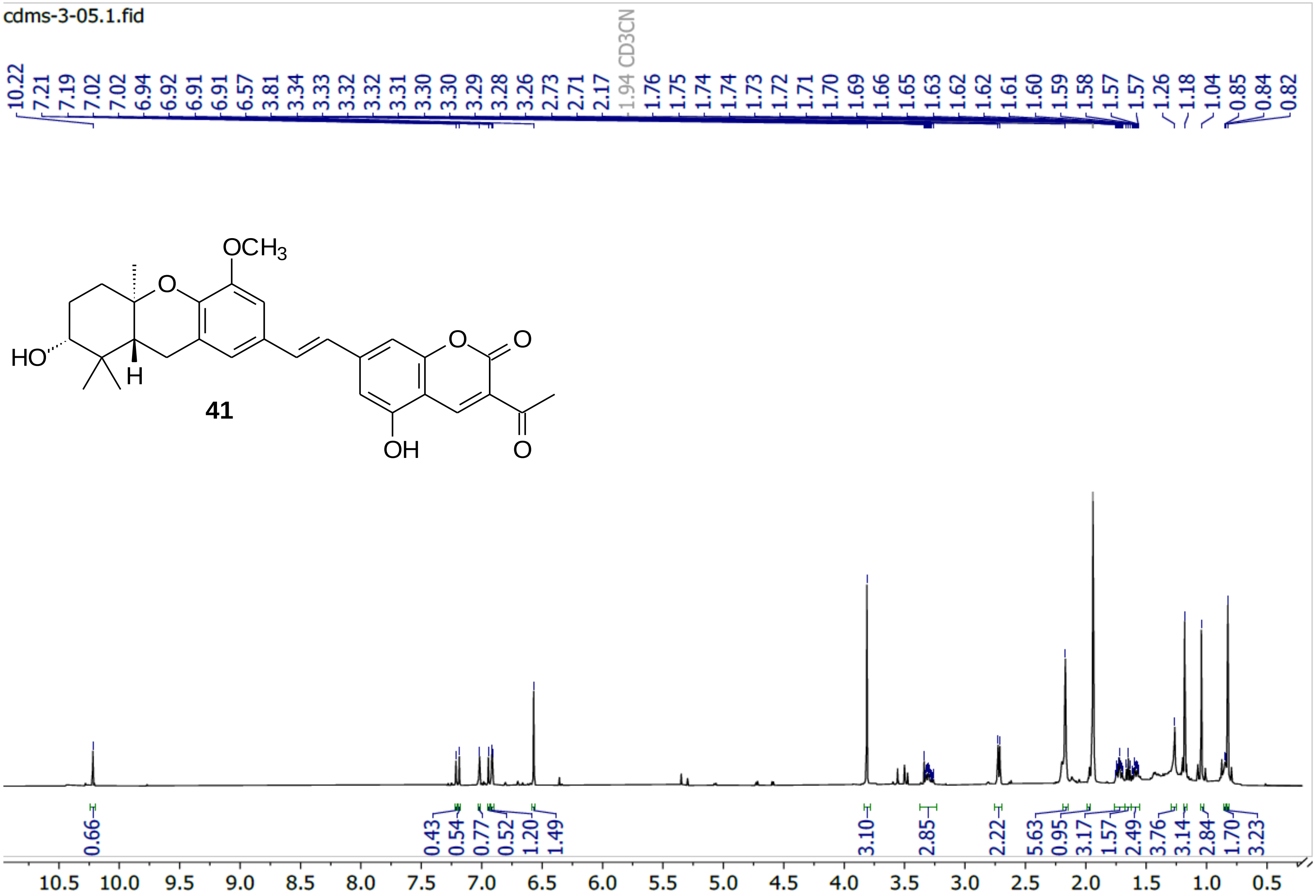

400 MHz ^{1}H NMR spectrum of **41** in CD_3CN

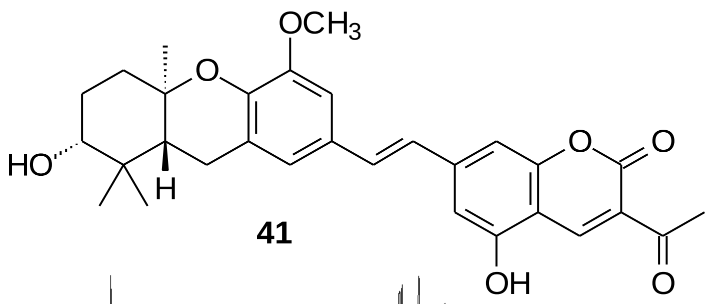

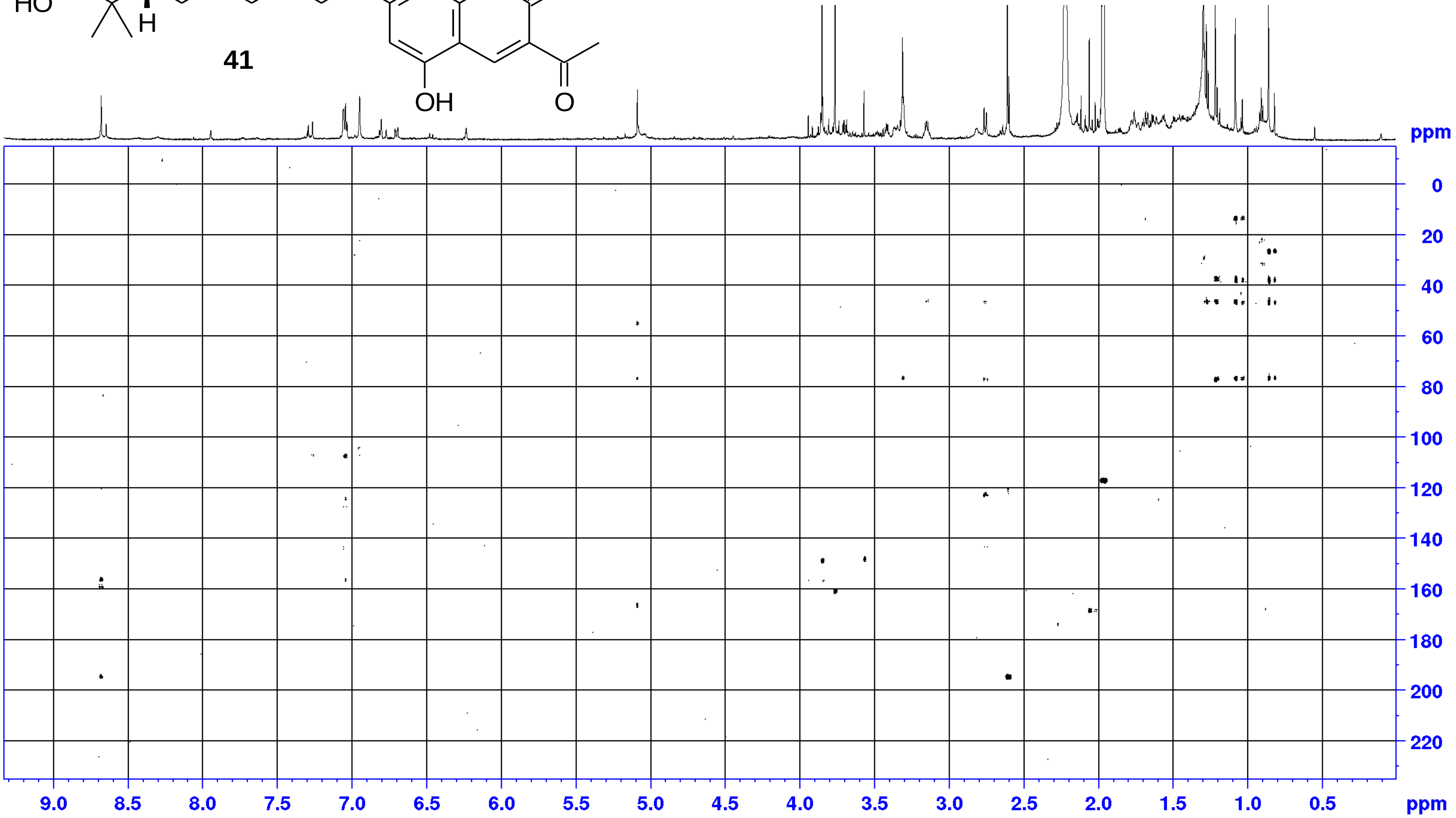

600 MHz 2D HMBC spectrum of **41** in CD_3CN

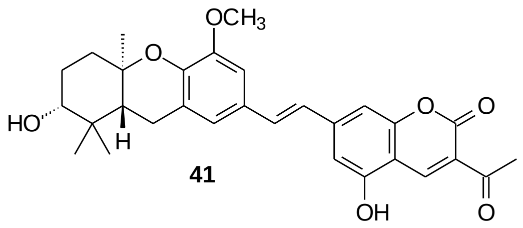

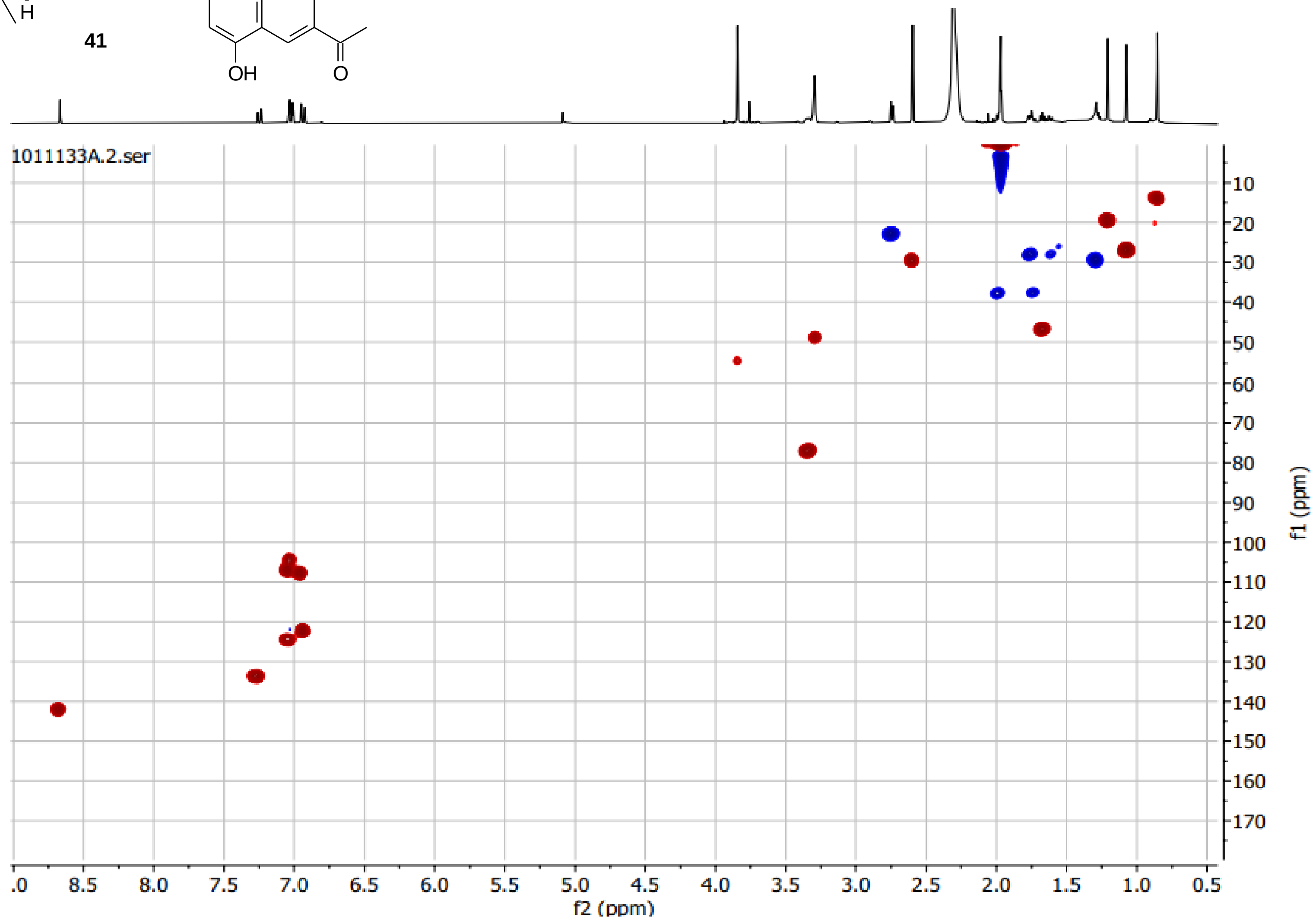

Phase sensitive HSQC spectrum of compound **41** in CD_3CN

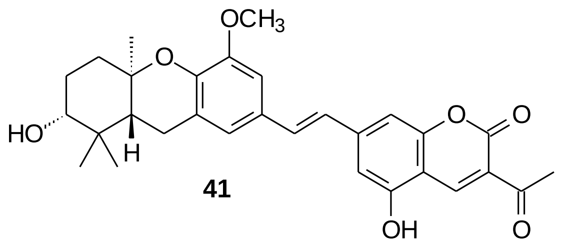

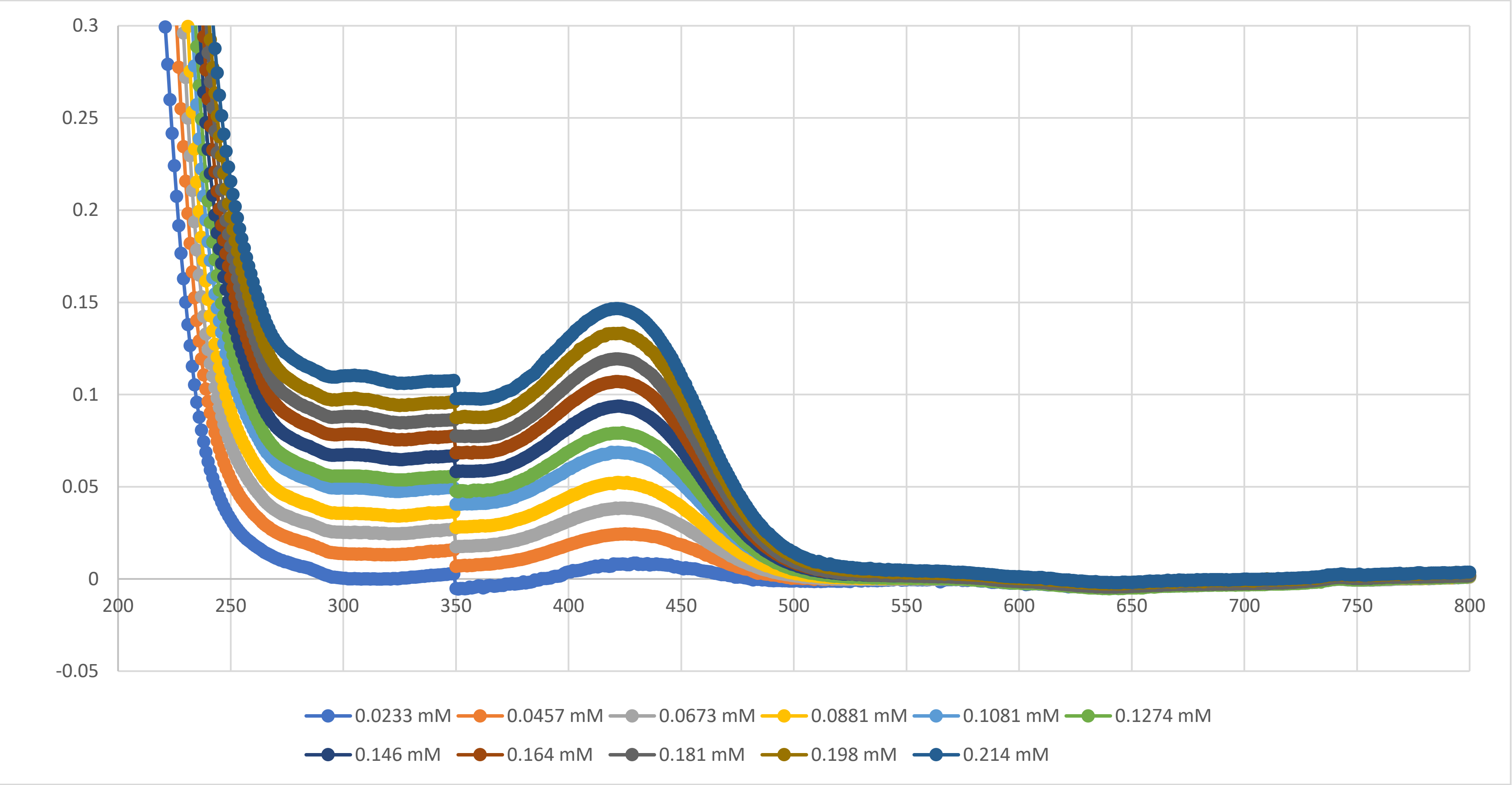

Absorption spectrum of compound **41** in EtOH

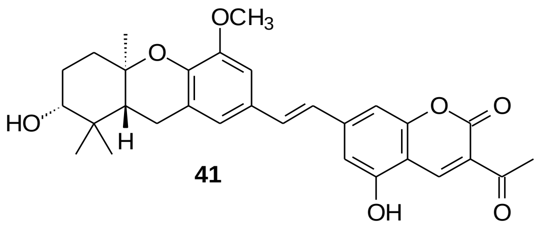

41

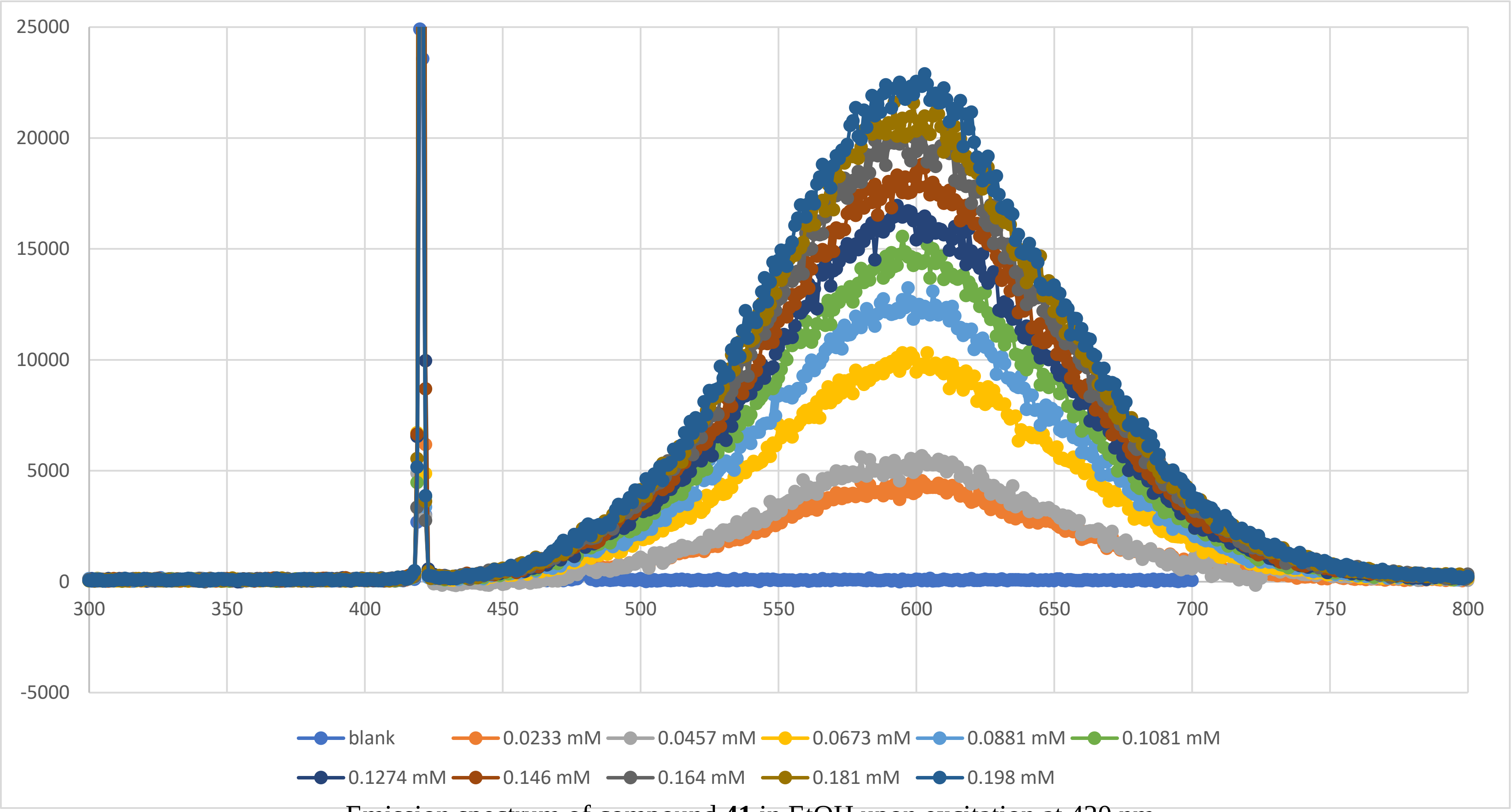

Emission spectrum of compound **41** in EtOH upon excitation at 420 nm

National Cancer Institute Developmental Therapeutics Program
Dose Response Curves

NSC: D - 823234 / 1	SSPL: 0GZS	EXP. ID: 2011RS62
Report Date: December 11, 2020	Test Date: November 16, 2020	

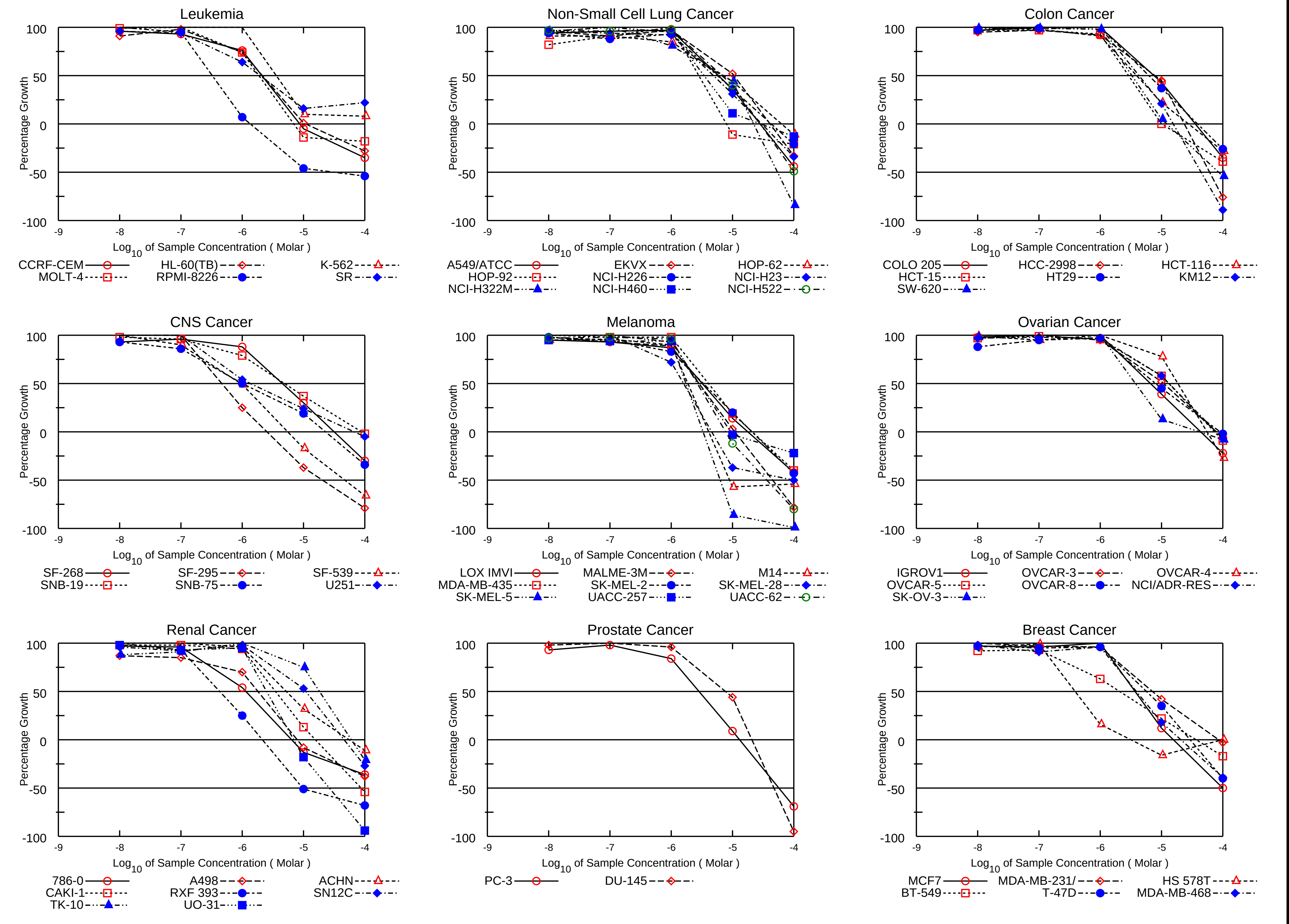

National Cancer Institute Developmental Therapeutics Program	NSC : D - 823234/1	Units :Molar	SSPL :0GZS	EXP. ID :2011RS62
Mean Graphs	Report Date :December 11, 2020		Test Date :November 16, 2020	

Panel/Cell Line	Log_{10}GI50	Log_{10}TGI	Log_{10}LC50
**Leukemia**			
CCRF-CEM	-5.68	-5.06	> -4.00
HL-60(TB)	-5.67	-4.97	> -4.00
K-562	-5.42	> -4.00	> -4.00
MOLT-4	-5.73	-5.16	> -4.00
RPMI-8226	-6.49	-5.86	-4.48
SR	-5.71	> -4.00	> -4.00
**Non-Small Cell Lung Cancer**			
A549/ATCC	-5.23	-4.55	> -4.00
EKVX	-4.98	-4.39	> -4.00
HOP-62	-5.14	-4.19	> -4.00
HOP-92	-5.52	-5.09	> -4.00
NCI-H226	-5.25	-4.38	> -4.00
NCI-H23	-5.31	-4.52	> -4.00
NCI-H322M	-5.15	-4.65	-4.26
NCI-H460	-5.47	-4.55	> -4.00
NCI-H522	-5.17	-4.55	> -4.00
**Colon Cancer**			
COLO 205	-5.12	-4.45	> -4.00
HCC-2998	-5.10	-4.62	-4.21
HCT-116	-5.41	-4.56	> -4.00
HCT-15	-5.54	-4.99	> -4.00
HT29	-5.21	-4.41	> -4.00
KM12	-5.36	-4.81	-4.35
SW-620	-5.48	-4.92	-4.07
**CNS Cancer**			
SF-268	-5.34	-4.50	> -4.00
SF-295	-6.29	-5.59	-4.69
SF-539	-6.01	-5.25	-4.33
SNB-19	-5.32	-4.04	> -4.00
SNB-75	-5.99	-4.64	> -4.00
U251	-5.87	-4.18	> -4.00
**Melanoma**			
LOX IMVI	-5.50	-4.75	> -4.00
MALME-3M	-5.54	-4.96	-4.35
M14	-5.73	-5.39	-5.05
MDA-MB-435	-5.40	-4.68	> -4.00
SK-MEL-2	-5.47	-4.68	> -4.00
SK-MEL-28	-5.80	-5.34	-4.03
SK-MEL-5	-5.76	-5.48	-5.20
UACC-257	-5.55	-5.03	> -4.00
UACC-62	-5.58	-5.11	-4.44
**Ovarian Cancer**			
IGROV1	-5.17	-4.36	> -4.00
OVCAR-3	-4.96	-4.10	> -4.00
OVCAR-4	-4.73	-4.26	> -4.00
OVCAR-5	-4.88	-4.13	> -4.00
OVCAR-8	-5.10	-4.04	> -4.00
NCI/ADR-RES	-4.88	-4.10	> -4.00
SK-OV-3	-5.37	-4.39	> -4.00
**Renal Cancer**			
786-0	-5.94	-5.19	> -4.00
A498	-5.74	-5.10	> -4.00
ACHN	-5.28	-4.26	> -4.00
CAKI-1	-5.46	-4.81	-4.06
RXF 393	-6.37	-5.67	-5.02
SN12C	-4.96	-4.33	> -4.00
TK-10	-4.74	-4.22	> -4.00
UO-31	-5.60	-5.16	-4.58
**Prostate Cancer**			
PC-3	-5.55	-4.89	-4.24
DU-145	-5.11	-4.68	-4.32
**Breast Cancer**			
MCF7	-5.44	-4.81	> -4.00
MDA-MB-231/ATCC	-5.15	-4.07	> -4.00
HS 578T	-6.41	-5.50	> -4.00
BT-549	-5.68	-4.43	> -4.00
T-47D	-5.25	-4.54	> -4.00
MDA-MB-468	-5.41	-4.69	> -4.00
_MID	-5.46	-4.7	-4.13
Delta	1.03	1.16	1.07
Range	1.76	1.86	1.2

GI50

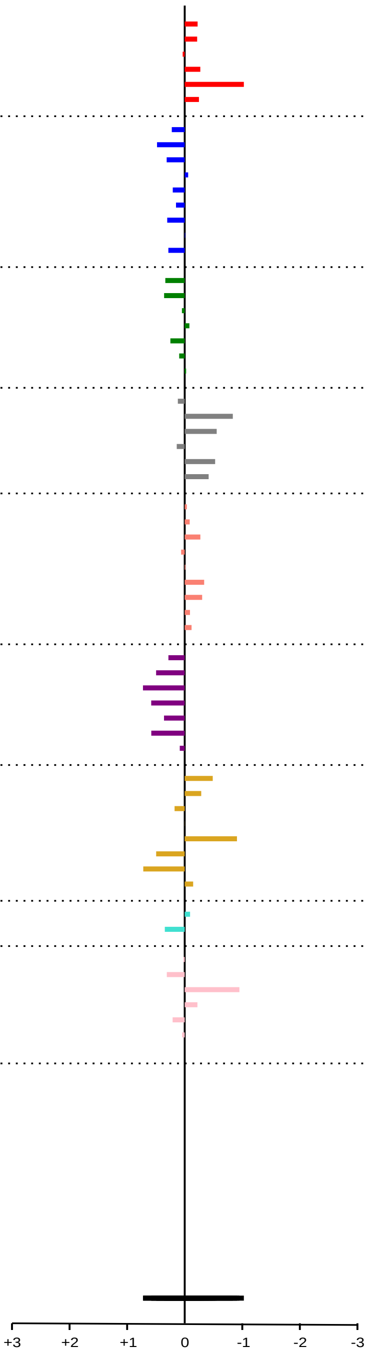

TGI

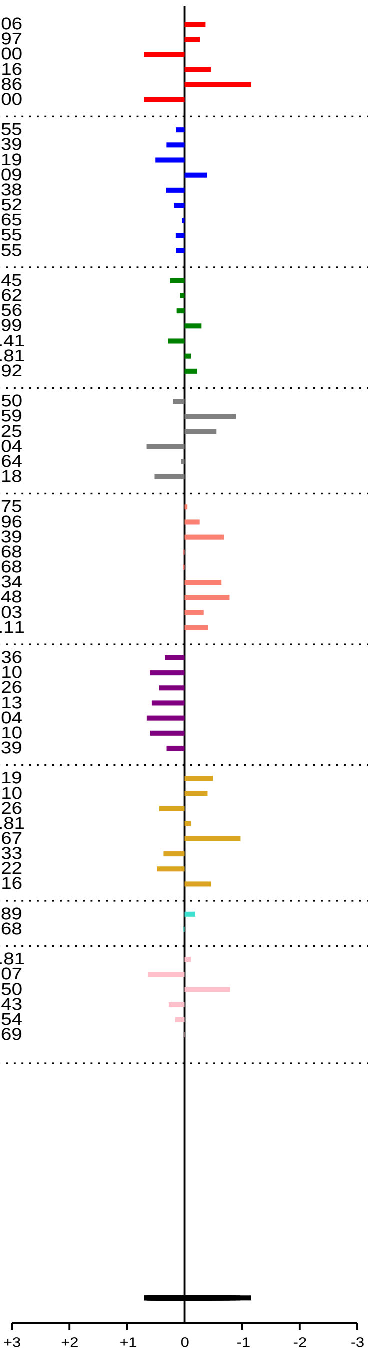

LC50

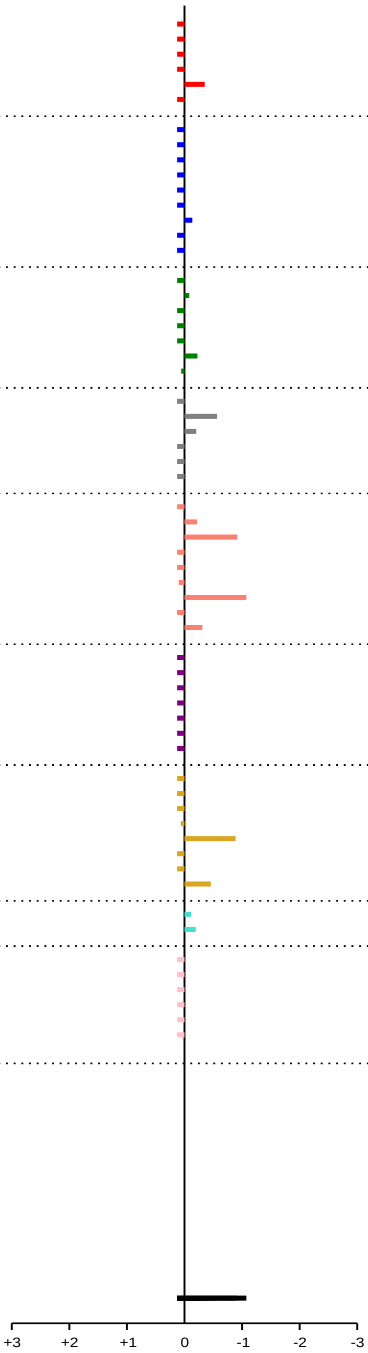

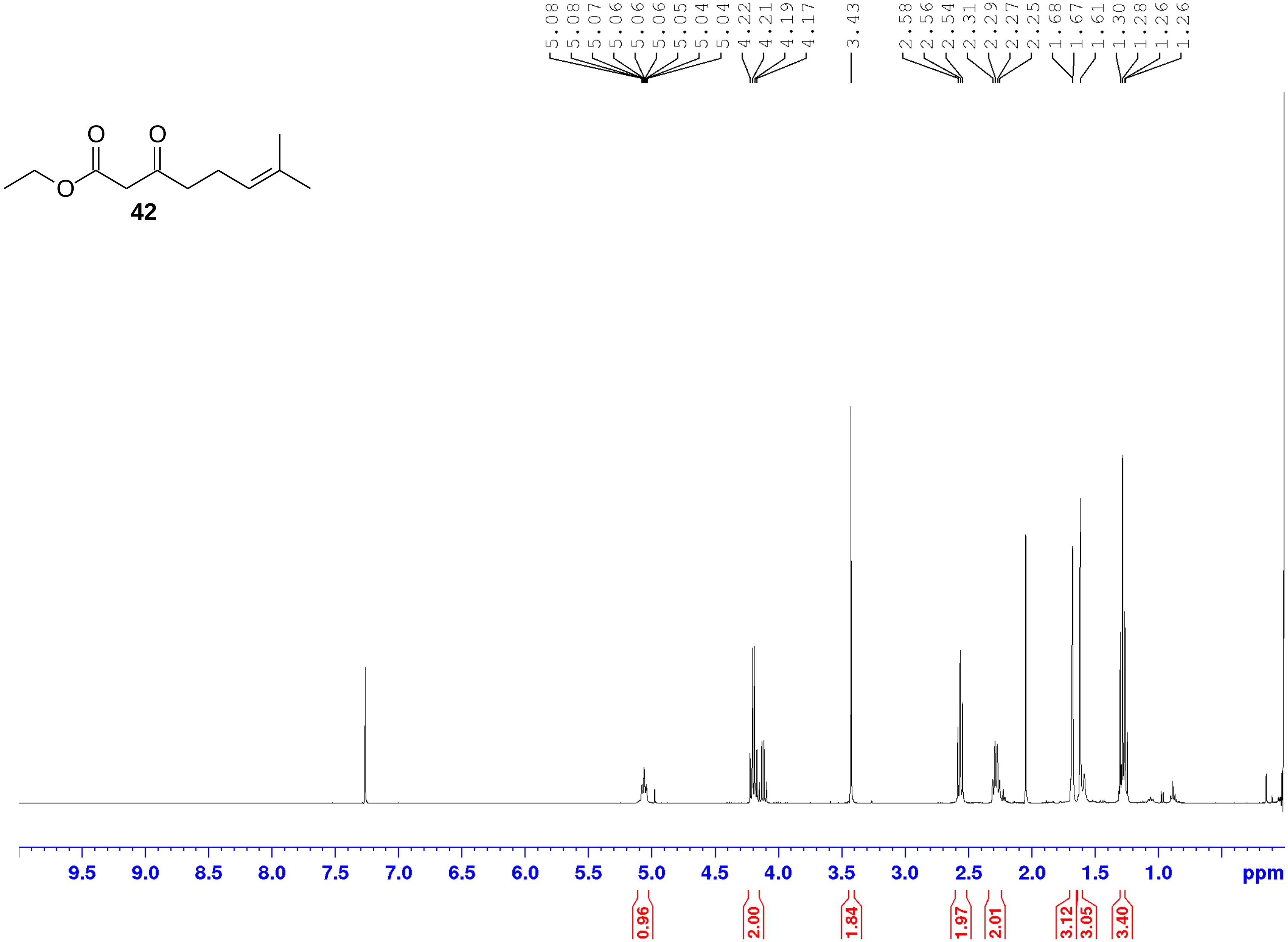

42

400 MHz ^{1}H NMR spectrum of **42** in $CDCl_3$

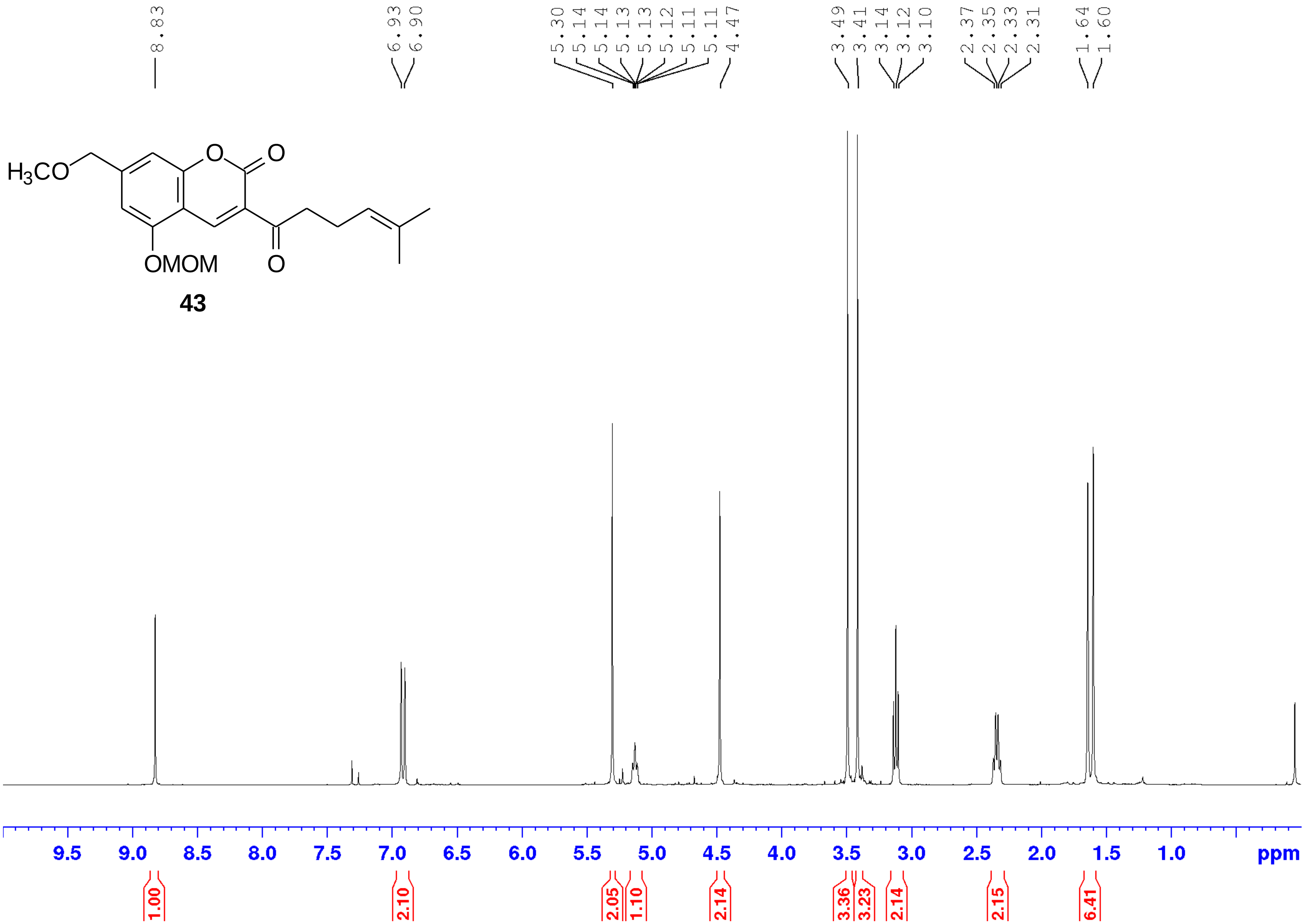

400 MHz ^{1}H NMR spectrum of **43** in $CDCl_3$

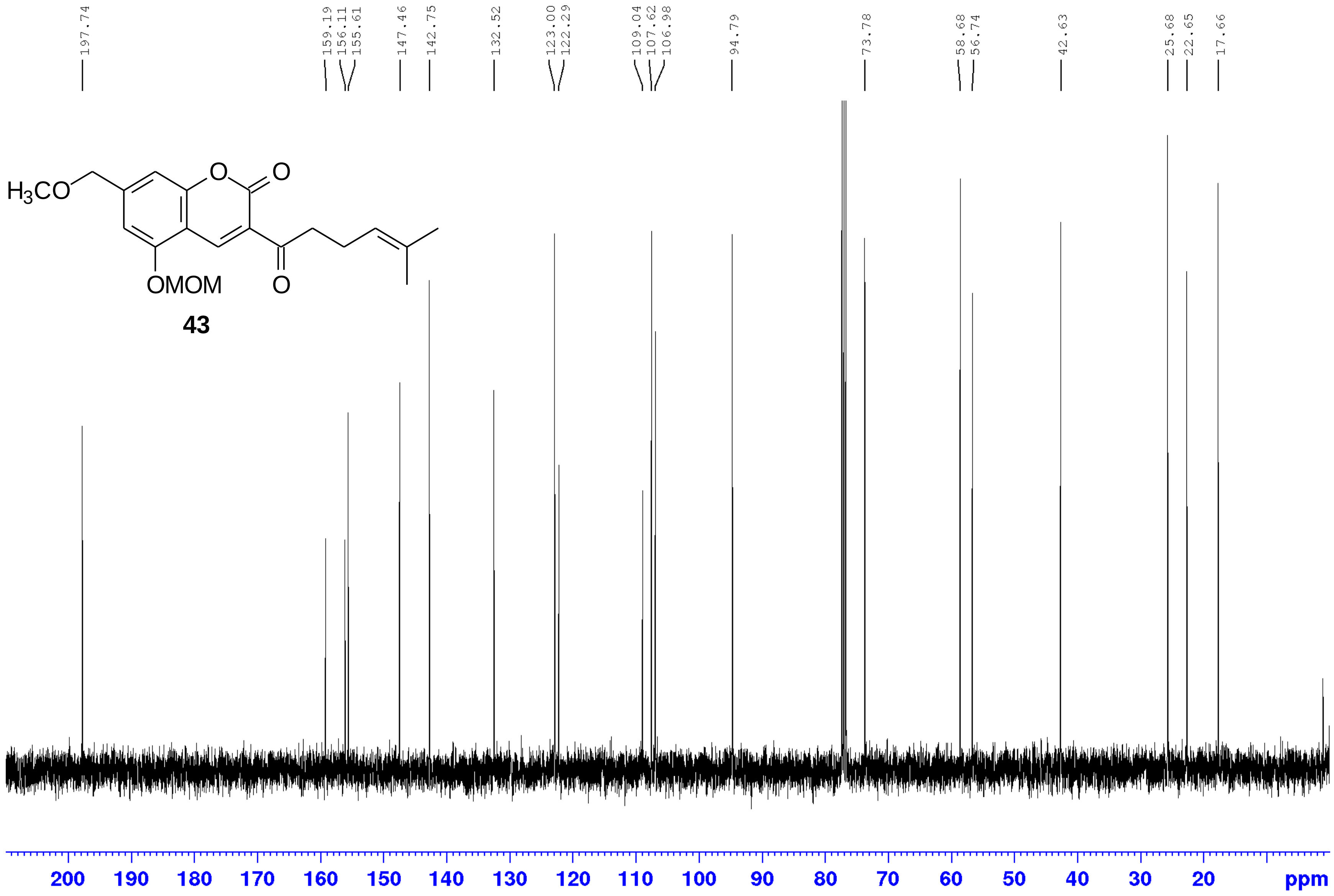

100 MHz $^{13}C\{^{1}H\}$ NMR spectrum of **43** in $CDCl_3$

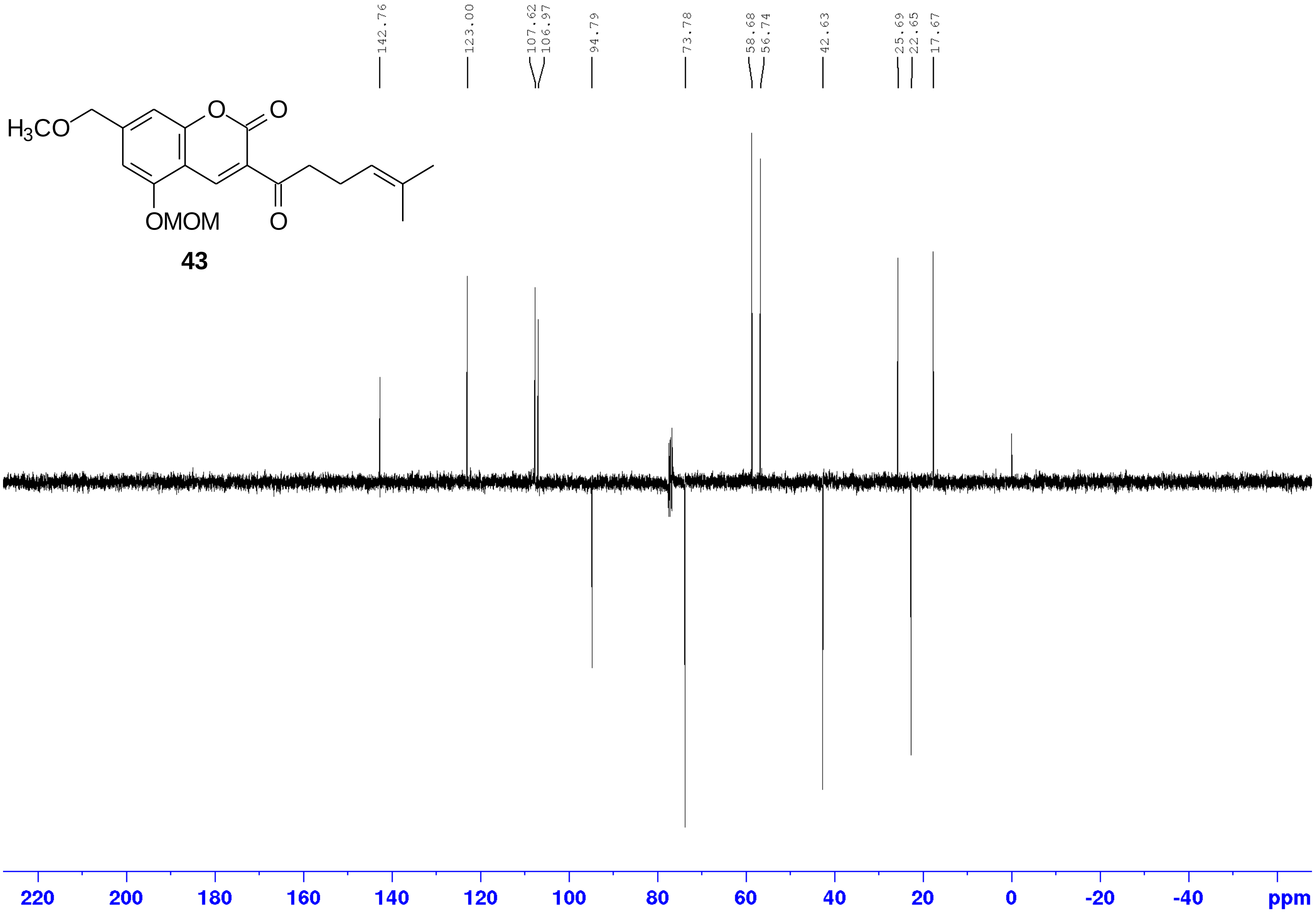

100 MHz DEPT-135 of **43** in $CDCl_3$

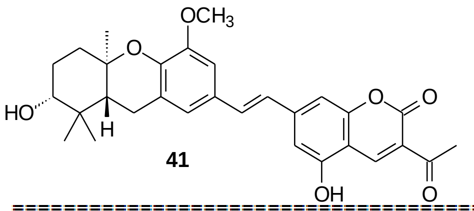

41

```
=====================================================================
Acq. Operator   : SYSTEM
Sample Operator : SYSTEM
Acq. Instrument : Shared 1220                    Location :   1
Injection Date  : 1/4/2020 11:38:42 AM                Inj :   1
                                               Inj Volume : No inj
Method          : C:\Users\Public\Documents\ChemStation\1\Methods\CMS-C18.M
Last changed    : 1/4/2020 11:44:21 AM by SYSTEM
                  (modified after loading)
=====================================================================
```

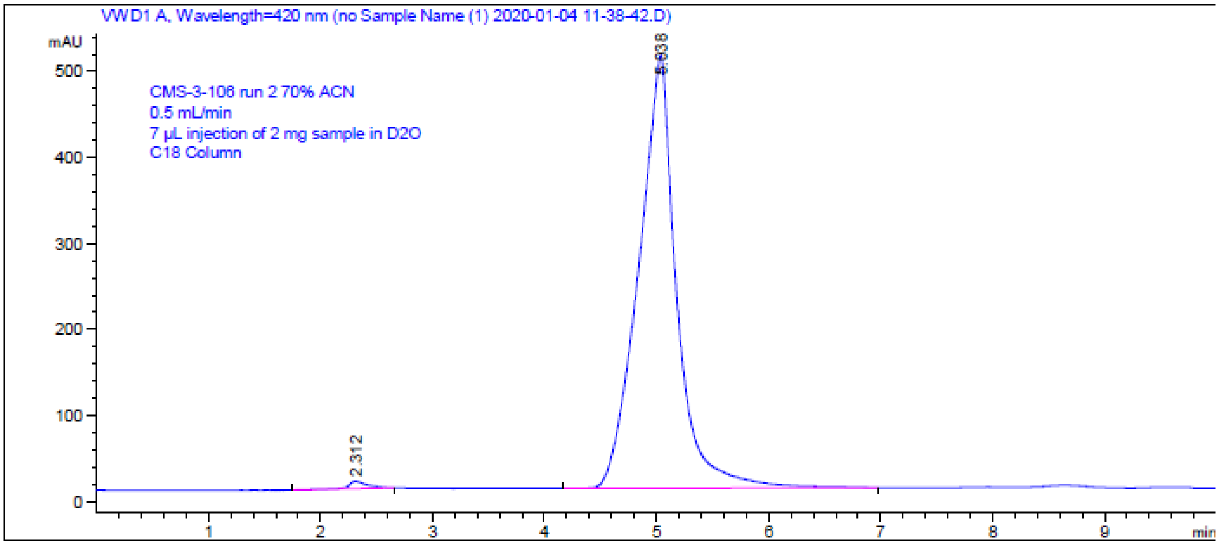


```
=====================================================================
                     Area Percent Report
=====================================================================

Sorted By             :      Signal
Multiplier            :      1.0000
Dilution              :      1.0000
Do not use Multiplier & Dilution Factor with ISTDs


Signal 1: VWD1 A, Wavelength=420 nm

Peak RetTime Type  Width     Area      Height     Area
  #   [min]        [min]   [mAU*s]     [mAU]        %
----|-------|----|-------|----------|----------|--------|
   1   2.312 BB    0.1650  105.00115    8.88003   0.8786
   2   5.038 BB    0.3136 1.18456e4  503.35434  99.1214

Totals :                  1.19506e4  512.23437
```

HPLC trace of compound **41**
